# Chirality transmission in macromolecular domains

**DOI:** 10.1038/s41467-021-27708-4

**Published:** 2022-01-10

**Authors:** Shankar Pandey, Shankar Mandal, Mathias Bogetoft Danielsen, Asha Brown, Changpeng Hu, Niels Johan Christensen, Alina Vitaliyivna Kulakova, Shixi Song, Tom Brown, Knud J. Jensen, Jesper Wengel, Chenguang Lou, Hanbin Mao

**Affiliations:** 1 Department of Chemistry and Biochemistry, Kent State University, Kent, OH 44242 USA; 2 Biomolecular Nanoscale Engineering Center, Department of Physics, Chemistry and Pharmacy, University of Southern Denmark, Campusvej 55, 5230 Odense M, Denmark; 3 ATDBio Ltd., Magdalen Centre, Oxford Science Park, 1 Robert Robinson Avenue, Oxford, OX4 4GA UK; 4 Department of Chemistry, University of Copenhagen, Thorvaldsensvej 40, 1871 Frederiksberg, Denmark; 5 Department of Chemistry, University of Copenhagen, Universitetsparken 5, 2100 Copenhagen, Denmark; 6 Department of Chemistry, University of Oxford, 12 Mansfield Road, Oxford, OX1 3TA UK

**Keywords:** Single-molecule biophysics, Bioconjugate chemistry, DNA, Peptides, Molecular self-assembly

## Abstract

Chiral communications exist in secondary structures of foldamers and copolymers via a network of noncovalent interactions within effective intermolecular force (IMF) range. It is not known whether long-range chiral communication exists between macromolecular tertiary structures such as peptide coiled-coils beyond the IMF distance. Harnessing the high sensitivity of single-molecule force spectroscopy, we investigate the chiral interaction between covalently linked DNA duplexes and peptide coiled-coils by evaluating the binding of a diastereomeric pair of three DNA-peptide conjugates. We find that right-handed DNA triple helices well accommodate peptide triple coiled-coils of the same handedness, but not with the left-handed coiled-coil stereoisomers. This chiral communication is effective in a range (<4.5 nm) far beyond canonical IMF distance. Small-angle X-ray scattering and molecular dynamics simulation indicate that the interdomain linkers are tightly packed via hydrophobic interactions, which likely sustains the chirality transmission between DNA and peptide domains. Our findings establish that long-range chiral transmission occurs in tertiary macromolecular domains, explaining the presence of homochiral pairing of superhelices in proteins.

## Abstract (web summary)

Chiral communication can propagate in secondary structures within the effective intermolecular force (IMF) range but it is not known whether long-range chiral communication exists between tertiary peptide structures. Here, the authors use single-molecule force spectroscopy to investigate chiral interaction between DNA duplexes/triplexes and peptide coiled-coils and demonstrate chiral communication beyond the IMF distance.

## Introduction

Chiral recognition is omnipresent in asymmetric reactions biased toward one of the two stereoisomers^1–3^. Beyond small molecules, chiral communications have been reported in secondary structures of foldamers and copolymers via a network of non-covalent interactions^4–7^. In copolymers, various modes of chiral communications have been found^8,9^. For foldamers and non-proteogenic peptides, chirality has been induced by different chiral modalities^10–18^, while screw-sense reversal has been observed^19–21^. In natural biomacromolecules, helical sense mismatch phenomena were noticed in the B-Z transition region of DNA double helices where the two opposite helical senses encountered, breaking one base pair while perturbing two adjacent bases to accommodate the torsional strain^22^.

However, all chiral communications demand close contact of neighbouring functional groups, including chiral inducers. In the contact interface, the interaction strength is determined by an ensemble set of intermolecular forces (IMF) between functional groups, which is effective on the length scale of Van der Waals radius^23^. Therefore, it remains elusive whether chiral-to-chiral communication is permitted beyond the IMF distance between higher-order macromolecular domains such as peptide tertiary structures. As one of the main protein tertiary structures^24,25^, peptide coiled-coil domains compose of multiple α-helices wrapped around one another to generate multimeric right- or left-handed helical structures^26^. The inter-strand helicity of these domains are mechanical in nature^27^. It is thus tantalizing to see whether chirality can be transmitted by mechanical interactions, which are long-range in nature.

**Fig. 1:**
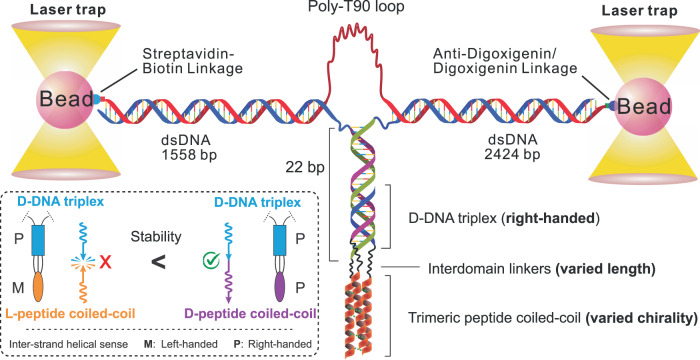
Schematic of optical-tweezers set up for high-throughput single-molecule assay. The inter-strand helical sense match/mismatch effects between the two macromolecular domains are shown in the bottom left inset in which the D-DNA triplex is marked in cyan rectangle, L-peptide coiled-coil in orange ellipse and D-peptide coiled-coil in purple ellipse. M and P stand for left-handed and right-handed inter-strand helical senses, respectively. The clash symbol depicts the helical sense mismatch state, which has unfavourable energy cost and thus is marked by a red-cross. The no clash symbol represents the helical sense match state, which should not influence the stability and thus is marked by a green check.

Compared to DNA helices, the peptide coiled-coil helices have much less inter-strand twisting in which α-helices wrap around one another^28,29^. As a result, the chiral communication between the inter-strand helical senses of two neighbouring protein coiled coils is difficult to detect by ensemble average approaches with low chiral sensitivities. Here we propose to employ single-molecule force spectroscopy to investigate whether chiral-to-chiral communication is permitted from enantiomeric trimeric peptide coiled coils to a DNA triplex (Fig. 1). The DNA triplex, composed of a triplex-forming oligonucleotide (TFO) binding to the major groove of a duplex DNA, has shown to facilitate peptide coiled-coil self-assembly^30–32^. To achieve this templating effect, each peptide strand was conjugated with a DNA strand, resulting in a peptide-oligonucleotide-conjugate (POC) in which the length of the interdomain linker can be varied. The chiral transmission was evaluated by the binding efficacy of the third POC to the underlying POC duplex.

Overall, we observed facile binding of the third POC strand to the POC duplex system, likely due to the templated binding effect in which conjugated peptide-oligonucleotide geometry significantly increased the effective concentration of either peptide or oligonucleotide. The sandwiched triple-stranded linker region (24–25 bonds) was previously found to be packed tightly for both L-peptide/D-DNA and D-peptide/L-DNA triple helices^30^, giving rise to a torsionally constrained intersection between two macromolecular domains. In analogy to the helical sense mismatch in the B-Z DNA junctions, when the two torsionally constrained, covalently conjugated macromolecular domains adopt opposite inter-strand helical senses, they may result in a less stable macromolecular state (Fig. 1, as exemplified by a hybrid DNA-peptide triple helix structure shown at the left in the inset). Relative to the case of helical sense match where DNA strands and peptide coiled coils have the same inter-strand helical sense (Fig. 1, right in the inset), this reduced stability would be manifested in compromised binding of the third POC strand to the POC duplex templates, which was confirmed by experiments. Small-angle X-ray scattering (SAXS) and molecular dynamics (MD) simulation revealed no direct electrostatic/hydrophobic contact between the two macromolecular domains. Instead, the three interdomain linkers adopted restricted conformations via hydrophobic interactions, which likely explained the chiral conduction between the trimeric peptide coiled-coil and the DNA triplex. These findings indicated that chiral communications are not only present in the secondary structures of copolymers and non-proteogenic peptides, but also exist between two biomacromolecular domains in a long-range manner (<4.5 nm), favouring the homochirality of neighbouring peptide coiled-coil domains in proteins.

## Results

### Design and synthesis of POC

**Fig. 2:**
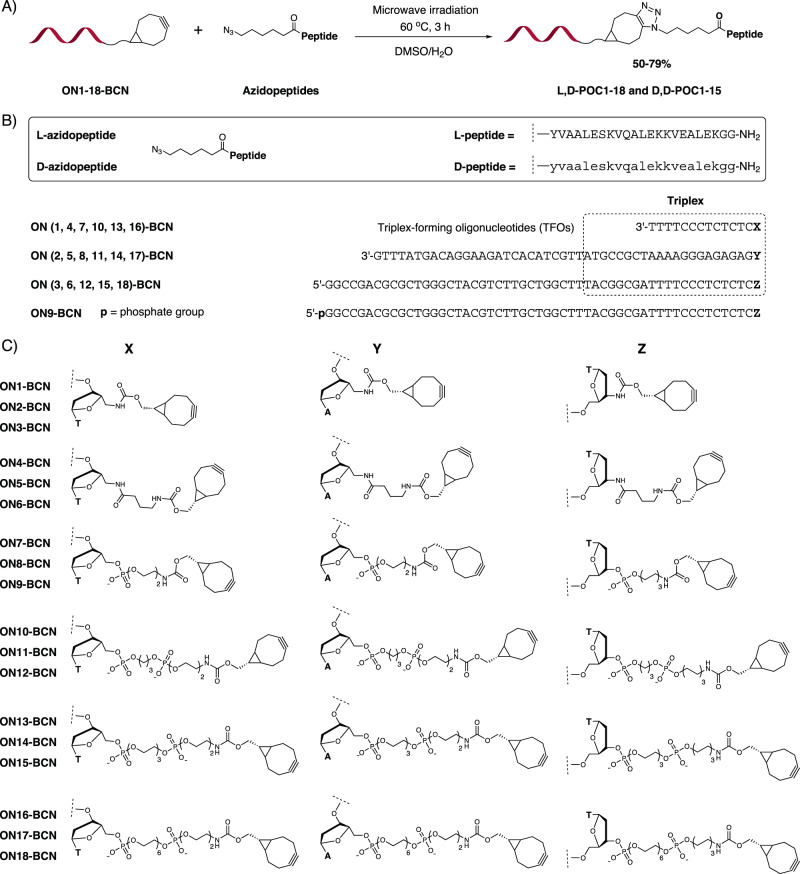
POC synthesis. **A** Conjugating two stereoisomeric azidopeptides to ON-BCNs via strain-promoted alkyne-azide cycloaddition to obtain **L,D-POC1-18** and **D,D-POC1-15**. **B** Sequence of **L-azidopeptide**, **D-azidopeptide** and ONs. **C** Different linker lengths were employed to furnish the BCN function either on the 5′-end or on the 3′-end of ONs. Natural amino acids are denoted in upper cases while unnatural ones are in lower cases. Thus, Y, V, L, E, S, K, Q, A, G are L-amino acids whereas y, v, l, e, s, k, q, a, g are D-amino acids. A, G, C, and T are natural DNA monomers. Key: The two-letter prefix before POCs: The first letter indicates the chirality of the peptide while the second letter indicates the chirality of the ON.

DNA helices were chosen as the first set of macromolecular helical domain, while two mirror-imaged coiled-coil peptide tertiary structures^30^ were employed as another set of macromolecular helical domains derived from coil-V_a_L_d_^33^. A series of linker lengths were introduced between the oligonucleotide triplex and the peptide tertiary structures, including 16 bonds, 21 bonds, 24–25 bonds, 30–31 bonds, 35–36 bonds and 44–45 bonds, each counting as the shortest path from 5′-/3′-position (5′-NH/3′-NH or 5′-O/3′-O) of the oligonucleotide (ON) triplex to the N-termini (NH of Tyr) of the peptide (Fig. 2).

The two peptides, **L-azidopeptide** and **D-azidopeptide**, were synthesized through solid-phase peptide synthesis^30^. For the ON triplex, automated DNA synthesis were carried out for eighteen ONs (see Supplementary Discussion). Phosphoramidite monomers **4**, **13**, **7**, **16** (Supplementary Figs. 1 and 2) and four commercially available monomers were incorporated to the 5′-end of ONs (**ON1**, **ON2**, **ON4**, **ON5**, **ON7**, **ON8**, **ON10**, **ON11**, **ON13**, **ON14**, **ON16** and **ON17**, Supplementary Fig. 10), while the synthesis of **ON3**, **ON6**, **ON9**, **ON12**, **ON15** and **ON18** (Supplementary Fig. 10) started from three special solid supports (3′-amino-dT CPG, **23** in Supplementary Fig. 3 and 3′-PT-amino-modifier C6 PS). After reverse-phase and/or ion-exchange HPLC purification, the composition and purity (>95%) of eighteen ONs were confirmed by ion-exchange HPLC, MALDI-MS and ESI-MS (Supplementary Figs. 4–9). A two-step synthetic strategy was used to prepare the POCs (Fig. 2 and Supplementary Figs. 10–44). A total of 33 POCs (Supplementary Fig. 45) were synthesized to assemble eleven designer POC triplexes with the interdomain linker length increasing from 16 bonds (**L,D-POC(1** + **2** + **3)**, **D,D-POC(1** + **2** + **3)**), 21 bonds (**L,D-POC(4** + **5** + **6)**, **D,D-POC(4** + **5** + **6)**), 24–25 bonds (**L,D-POC(7** + **8** + **9)**, **D,D-POC(7** + **8** + **9)**), 30–31 bonds (**L,D-POC(10** + **11** + **12)**, **D,D-POC(10** + **11** + **12)**), 35–36 bonds (**L,D-POC(13** + **14** + **15)**, **D,D-POC(13** + **14** + **15)**) and 44–45 bonds (**L,D-POC(16** + **17** + **18)**). Only L,D-POCs were synthesized for the longest linker with 44–45 bonds. The right-handed helical sense was always maintained for the DNA triplex, while the inter-strand screw sense of coiled coils varied from left-handedness to right-handedness when L-peptide coiled-coil was replaced with D-peptide coiled-coil (Fig. 1).

### Formation of trimeric coiled-coil peptides increases the mechanical stability of DNA triplex templates

We used a single-molecule mechanical platform in optical tweezers to investigate the repetitive formation and dissociation of D-peptide or L-peptide coiled coils assisted by the DNA templates (Supplementary Fig. 46). Each single-stranded DNA fragment of the assembled dimeric POC assembles was hybridized with a complementary DNA overhang at the end of a duplex DNA handle. The free end of one DNA handle was labelled with digoxigenin, while that of the other DNA handle was labelled with biotin. The digoxigenin and biotin ends were then bound to two optically trapped beads coated with digoxigenin antibody and streptavidin, respectively (Fig. 1). A polythymine (T90) linker was used to connect the two DNA handles from the non-labelled ends, so that the tethered POC strands can be kept in proximity^34^ to each other. This facilitated the reassembly of the POC complexes at the reduced force after they were disrupted mechanically at higher force.

**Fig. 3:**
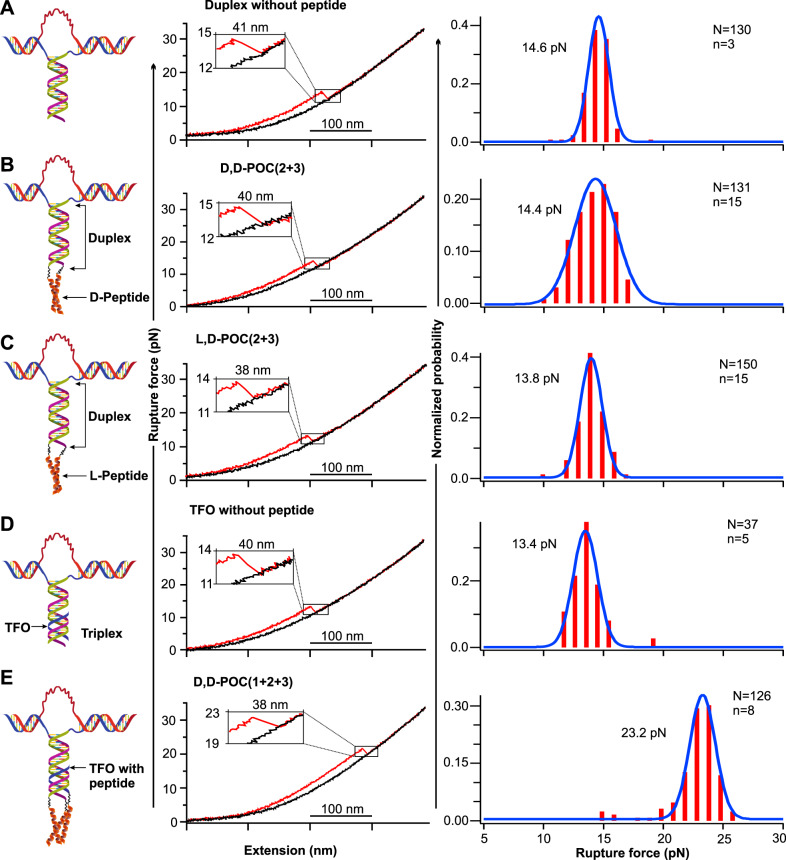
Effect of TFO or D,D-POC1 on the mechanical stability of the DNA duplex or the chimeric POC duplex D,D-POC(2 + 3). Schematic diagrams, typical force-extension curves and corresponding force histograms of (**A**) DNA duplex control, (**B**) **D,D-POC(2** + **3)**, (**C**) **L,D-POC(2** + **3)**, (**D**) DNA triplex control, (**E**) **D,D-POC(1** + **2** + **3)**. **TFO** = 3′-TTTTCCCTCTCTCT. Note: red and black traces in a force-extension curve represent stretching and relaxing events, respectively. *N* and *n* depict total numbers of features and distinct molecules, respectively.

Two POC fragments were brought closer with the help of a steerable mirror, which moved one of the optically trapped beads with respect to the other. The hybridized duplex DNA domain served as a template to assemble dimeric coiled-coil peptides in the POC duplex. Since acidic condition is required to form stable POC triple helices (protonation of N3 of cytosine to provide an extra H-bond in the ON triplex^35,36^), pH 5.5 was maintained throughout all the experiments. By moving optically trapped beads away from each other, the tension accumulated in the molecular construct was solely exerted on the dimeric POC complex. The increased tension eventually dissociated the two POC strands, which was recorded in real-time in the force-extension curves (Fig. 3B, middle). Using the DNA duplex as the control, experiments were firstly carried out for two dimeric POC complexes, **D,D-POC(2** + **3)** and **L,D-POC(2** + **3)**, all of which uniformly gave a rupture force at ~14 pN (Fig. 3A–C). Thus, dimeric POC complexes did not yield differentiating signals to probe the proposed long-range chirality effect on the topology of higher-order macromolecular structures. The mechanical stability of the dimeric **L,D-POC(2** + **3)** complex (13.8 pN, Fig. 3C) was found to be slightly lower than the corresponding **D,D-POC(2** + **3)** complex (14.4 pN, Fig. 3B).

We then flowed the **TFO/D,D-POC1** (1 µM) into the chamber at the same pH (Fig. 1A, bottom), which resulted in the formation of the triplex by the Watson-Crick and Hoogsteen base-pairing (Fig. 3D)^37,38^. When **TFO** (Supplementary Fig. 47) bound to the single-molecule DNA duplex target, mechanical unfolding revealed that the 13.6 pN as the unzipping force for the DNA triplex, indicating a weaker apparent mechanical stability compared to the DNA duplex (14.6 pN, Fig. 3A), which was consistent with literature observation^39^. The reduced mechanical stability was interpreted as a minority of DNA duplex formation being perturbed as the result of the **TFO** injection. In the next experiment, we replaced the DNA triplex template with three POC strands to assemble the chimeric POC triplex where the formation of the DNA triple helix, and the trimeric peptide coiled-coil were cooperative^30^. This chimeric supramolecular complex **D,D-POC(1** + **2** + **3)** showed much stronger mechanical stability (23.2 pN, Fig. 3E), indicating that the synergy between the peptide domain and the nucleic acid template significantly improved the mechanical stability of the underlying DNA triplex scaffold. These results validate that trimeric POC complexes may serve as ideal macromolecular tools to determine the helical sense mismatch effect for macromolecular topologies since the synergistic formation of the chimeric POC triplex can be unequivocally distinguished by the 23 pN unfolding force populations.

### Long-range chirality transmission between natural nucleic acid domain and peptide domain in a distance-dependent manner

**Fig. 4:**
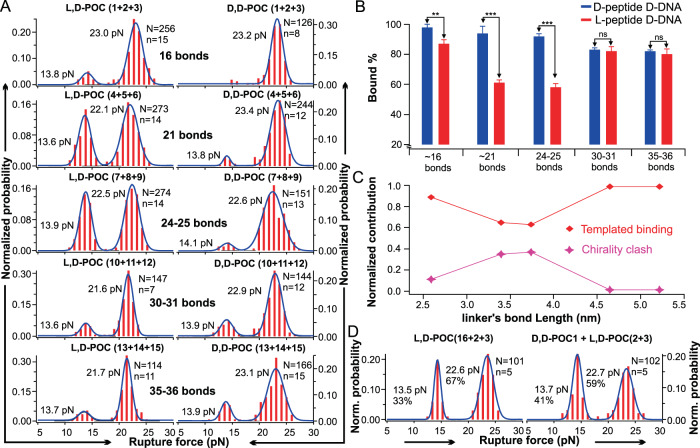
Comparative study of bound % for the L- and D-peptide with different linker lengths. **A** Rupture force histograms for the L-peptide D-DNA (left) and D-peptide D-DNA (right) with varying linker length (in number of bonds). **B** Comparison of bound % for D-peptide D-DNA and L-peptide D-DNA. Data are presented as mean ± SD for *n* = 4 independent experiments. **P = 0.0085, ***P = 3.9 × 10^−5^ for the 21 bonds and *P* = 2.4 × 10^−5^ for the 24–25 bonds, ns: nonsignificant (two tailed unpaired *t*-test). **C** Percentage contribution of chirality clash and templated binding effects for the trimeric L-peptide D-DNA with varied linker length (see Methods for calculation). **D** Rupture force histograms for the L-peptide D-DNA (**L,D-POC(2** + **3)**, 16 bonds) bound with a TFO conjugated with L-peptide (**L,D-POC16**, 44–45 bonds, left) or with D-peptide (**D,D-POC1**, 16 bonds, right). *N* and *n* depict total numbers of features and distinct molecules, respectively.

When we compared the 23 pN population between the two **D,D-POC(1** + **2** + **3)** and **L,D-POC(1** + **2** + **3)** trimeric coiled-coil diastereomers, the latter species showed a lower formation percentage (98% vs 87%, at 99.99% confidence level, Fig. 4A). Given that the DNA triplex and the D-peptide coiled-coil shared the same inter-strand screw sense, whereas the DNA triplex and L-peptide coiled-coil had the opposite helicities, we reasoned that the inter-strand screw-sense mismatch in the DNA-peptide interface may disfavour the synergistic formation of the chimeric POC triplex. The lower force populations (13 pN, 13%) for **L,D-POC(1** + **2** + **3)** were presumed to originate from the dissociation of the POC duplex, where there was no binding for **L,D-POC1**. These observations encouraged us to pursue more confirmative evidence for the chirality communication between two macromolecular domains.

Thus, as a continued step we varied the length of the linker bridging the DNA and the peptide domains, which was extended to 21 bonds (**L,D-POC(4** + **5** + **6)**, **D,D-POC(4** + **5** + **6)**), 24–25 bonds (**L,D-POC(7** + **8** + **9)**, **D,D-POC(7** + **8** + **9)**), 30–31 bonds (**L,D-POC(10** + **11** + **12)**, **D,D-POC(10** + **11** + **12)**), 35–36 bonds (**L,D-POC(13** + **14** + **15)**, **D,D-POC(13** + **14** + **15)**) and 44–45 bonds (**L,D-POC(16** + **17** + **18)**) (Supplementary Fig. 45). For each linker, we determined the unfolding force and percentage formation of the chimeric POC triplex (Fig. 4A, B). Two general trends were observed: (1) The mechanical stability of either lower- or higher- force population in homochiral D-peptide/D-DNA complexes always appeared higher than the corresponding species in heterochiral L-peptide/D-DNA complexes, congruent with the results obtained from **L,D-POC(2** + **3)** and **D,D-POC(2** + **3)**, as well as from **L,D-POC(1** + **2** + **3)** and **D,D-POC(1** + **2** + **3)**. While the DNA triple helix held the same right-handedness, considering the opposite mechanical chirality in the two mirror-imaged trimeric peptide coiled coils, these results clearly supported our hypothesis that the unmatched inter-strand screw-sense reduced the mechanical stability of the dimeric and trimeric POC assemblies. (2) By comparing the population percentage of the POC triple helix, the homochiral complexes (D-peptides and D-DNA) were more favoured than the heterochiral complexes (L-peptide and D-DNA) at each linker length. This further validated the presence of the postulated remote chirality transmission between the employed nucleic acid and peptide structures.

Interestingly, when we plot out the populations of the trimeric coiled coils vs linker length (Fig. 4B), we found that the D-peptide/D-DNA system had a monotonic decrease in population, whereas the L-peptide/D-DNA had a minimum triplex peptide population when the linker length was 24–25 bonds in **L,D-(POC7** + **POC8** + **POC9)**. For the D-peptide/D-DNA system, the decrease in the chimeric triplex population over the linker distance was probably due to the weakened templated binding effect (which gradually reduced when the interdomain linker was elongated). In marked contrast, for the L-peptide/D-DNA, the templated binding effect and the helical sense mismatch effect had the opposite momentum, thus resulting in a fluctuated population landscape for the chimeric triple helix. An exceptional example was when the linker became relatively short (16 bonds in **L,D-POC(1** + **2** + **3)** and **D,D-POC(1** + **2** + **3)**), due to the predominating templated binding effect, high chimeric trimer populations were observed in both cases irrespective of D- or L-peptide. When the interdomain linker was elongated from 16 bonds (2.6 nm) to 21 bonds (3.4 nm), the helical sense mismatch effect became significantly more pronounced. When it was further extended to 24–25 bonds (3.7 nm), the helical sense mismatch effect culminated, inhibiting the chimeric triplex population to a minimum level for the L-peptide/D-DNA system. This trend confirmed the long-range chirality transmission between the two macromolecular domains through three long interdomain linkers. Though each linker has more than half of the bonds with rotational freedom, we envisioned that packing three linkers between the two macromolecular domains may convert them to a superhelical conductor^22^ by forcing them to adopt restricted conformation(s) (vide infra). At even longer linker length (30–31 bonds and beyond), the helical sense mismatch effect sharply faded away close to zero level.

To quantify the relative contribution of the templated binding effect and the helical sense mismatch effect, we determined the percentage of the helical sense mismatch contribution for L-peptide/D-DNA (Fig. 4C). Assuming there was no helical sense mismatch effect in the D-peptide/D-DNA system, the maximum templated binding effect (100%) was determined by the percentage population of the D-peptide/D-DNA triplex at each linker length. On the other hand, when there was 100% helical sense mismatch, the L-peptide/D-DNA triplex population would be reduced to zero. By comparing the percentage formation of the two diastereoisomeric POC triplexes against these two extreme conditions (see Methods section), the percentage contribution of the helical sense mismatch effect for the L-peptide/D-DNA was determined at each linker length (Fig. 4C). From this analysis, a clear trend was noticed that the helical sense mismatch effect was long-range, reaching a maximum at 3.7 nm (contour length) before quickly diminishing to zero beyond 4.5 nm (contour length). The templated binding effect was somehow more profound. For instance, at 6.6 nm (linker with 44–45 bonds, Supplementary Fig. 45) the templated binding effect still persisted to facilitate the formation of the trimeric coiled coils (**L,D-POC(16** + **17** + **18)**, Supplementary Fig. 48A). All investigated POC duplexes/triplexes gave almost identical values in change-in-contour-length (Supplementary Fig. 49), confirming that the unfolding of POC triplexes took place when the underlying POC duplexes were ruptured.

To provide further evidence for the helical sense mismatch effect and the templated binding effect, we varied the interdomain linker length as well as the chirality of the peptide in the third POC, which was added through microflow injection. First, we used **L,D-POC(2** + **3)** (linker length, 16 bonds) and flowed **L,D-POC16** (linker length, 44–45 bonds). We observed that the percentage formation of the homochiral chimeric triplex was decreased to 67% (Fig. 4D, compared to 87% in Fig. 4A, top left panel). Similarly, when we tested the binding of **L,D-POC(17** + **18)** (linker length, 44–45 bonds) in the presence of **L,D-POC1** (linker length, 16 bonds), we again observed decreased homochiral triplex formation (from 80% [**L,D-POC (16** + **17** + **18)**] to 52% [**L,D-POC (1** + **17** + **18)**] (Supplementary Fig. 48). In both cases, the inter-strand screw-sense mismatch was preserved between the DNA triplex and the L-peptide coiled-coil trimer. Thus, the reduced triplex formation could be ascribed to the entropic penalty that originated from mismatched linker lengths between 16 bonds and 44–45 bonds. Therefore, these two experiments proved that the templated binding effect mediated by the same or similar linker length is important for the trimeric coiled-coil formation. In another control, we investigated the binding of **D,D-POC1** (linker length, 16 bonds) to the **L,D-POC(2** + **3)** (linker length, 16 bonds) single-molecule target, both of which had the same linker length but opposite chirality in the peptide domain. We found that the percentage formation of the trimeric POC ensemble was decreased to 59% (Fig. 4D, compared to 87% in Fig. 4A, top left panel). Such a result demonstrated that a perfect chirality match in different domains was indispensable to achieve the desired stabilizing synergy between the trimeric peptide coiled-coil and the DNA triplex scaffold. Finally, to explore the possible kinetic influence, we performed the single-molecule unfolding experiment for the L,D-POC system with 24–25 bonds linker length by decreasing the time window for POC triplex formation (incubation time at 0 pN after each unfolding event) from 60 to 15 s (Supplementary Fig. 50). We found percent formation of **L,D-POC (7** + **8** + **9)** trimeric complex was 61%, which was very close to the 58% formation at 60-second incubation time, indicating binding of the third POC strand reached equilibrium in our single-molecule experiments.

CD spectra were also recorded on all eleven POC triplex assemblies with the two ssDNA regions fully paired with their DNA complements. After subtracting the signatures contributed by the ON triplex (a DNA triplex control was used) to isolate the signal from the peptide structures, strong α-helicity was observed for all eleven POC triplex ensembles (both L-peptide and D-peptide, Supplementary Fig. 51), indicating that the formation of the coiled-coil structure was not significantly perturbed when the interdomain linker was elongated from 16 to 44–45 bonds. These experiments supported the premise that high force populations in mechanical unfolding were indeed from trimeric POC assemblies.

In addition, non-denaturing polyacrylamide gel electrophoresis was carried out for 15 µM of **TFO alone**, **L,D-POC7** and **D,D-POC7**, to eliminate the possibility that different intramolecular/intermolecular complex formations of the third POC strands accounted for the obtained single-molecule experimental results (Supplementary Fig. 52). Both **L,D-POC7** and **D,D-POC7** gave their bands near the 35-mer DNA marker, located very close to the monomeric state of the **L,D-POC(8** + 9)*^31^ but far away from the **TFO alone** control. This indicated that both **L,D-POC7** and **D,D-POC7** likely adopted fully extended conformations. Self-dimerization close to 75-mer DNA marker was also noticed (~8% for **D,D-POC7** and <5% for **L,D-POC7**, quantified in ImageJ), probably via intermolecular peptide interactions. However, considering the lower concentration (1 µM) used in the single-molecule experiments, which disfavoured the intermolecular interactions, we expect intermolecular interaction of the third POC strands had little influence on the bound fraction of the POC triplexes for **L,D-POC(7** + **8** + **9)** and **D,D-POC(7** + **8** + **9)**.

### SAXS analysis and MD simulations

To investigate the molecular mechanism of the chiral transmission between trimeric peptide coiled coils and DNA triplex, we performed SAXS and molecular modelling studies on the diastereoisomeric pair of **D,D-POC(7** + **8** + **9)** and **L,D-POC(7** + **8** + **9)** since they represent the highest change in the triplex population (96% vs 58%). To ease the experimental work, the two ssDNA regions extended from the underlying POC duplex were truncated, leading to the corresponding two diastereoisomers **D,D-POC(7** + **8** + 9)* and **L,D-POC(7** + **8** + 9)* (Supplementary Fig. 53). The SAXS characterization of **D,D-POC(7** + **8** + 9)* (Supplementary Fig. 54) showed the signals strongly resembled its diastereoisomer **L,D-POC(7** + **8** + **9**)* (Supplementary Fig. 55)^30^, suggesting formation of well-defined structures. The long-tailed real-space distance distribution function shown in Supplementary Fig. 54c confirmed that the conformation(s) of **D,D-POC(7** + **8** + 9)* were extended in the given buffer solution^40^. MD simulations indeed demonstrated more distinct and compact conformations after ~100 ns compared to the extended initial structure (Supplementary Figs. 56 and 57). While no direct electrostatic/hydrophobic contact between the DNA triplex and the trimeric peptide coiled-coil was detected in the MD ensembles (Supplementary Movie 1), the best SAXS fit from a single MD snapshot (representing a significantly populated group of compact conformations) depicted a bend in the linker region (Supplementary Fig. 58).

**Fig. 5:**
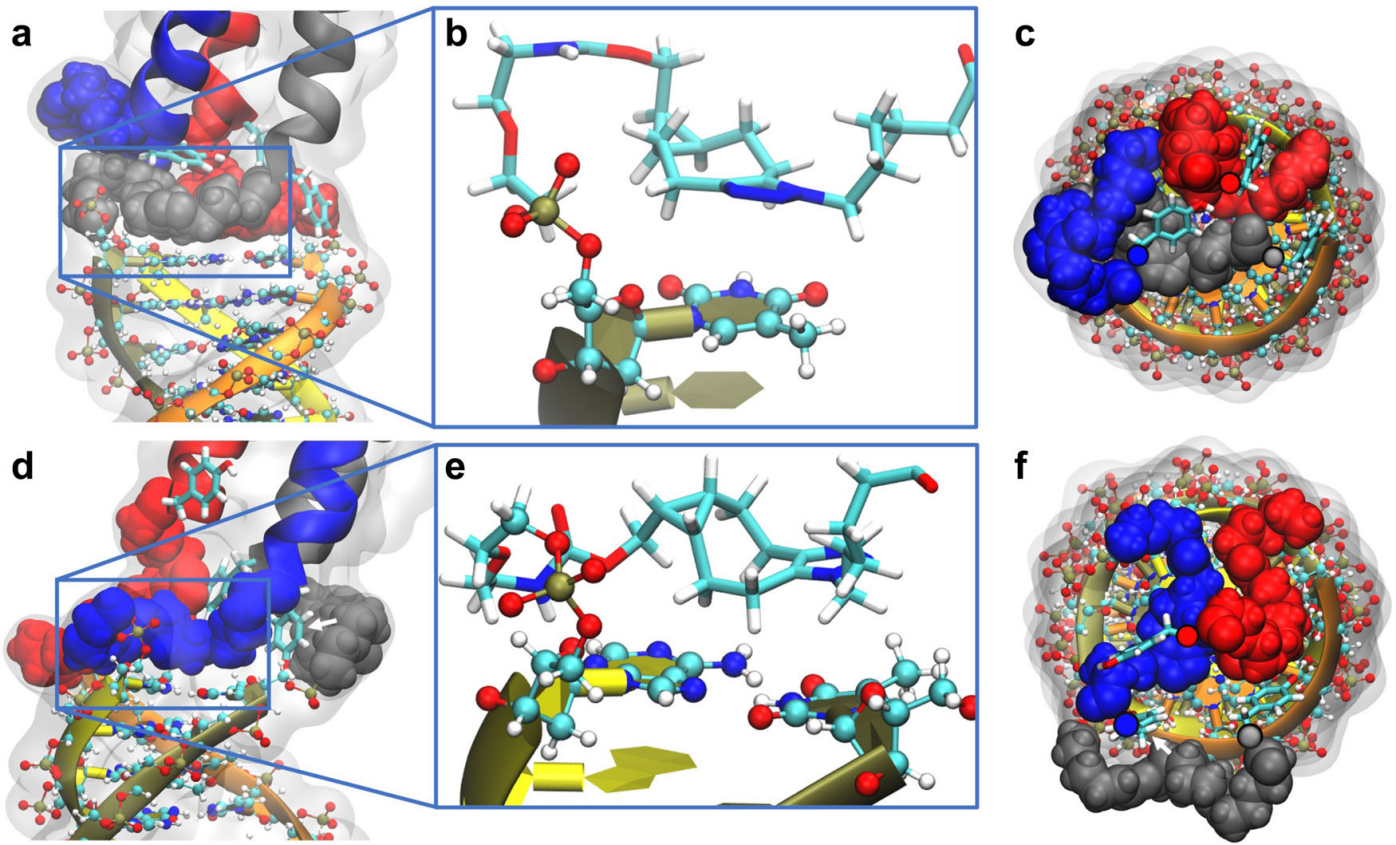
MD simulation. Packing of the linker region in the MD snapshots used for the SAXS prediction for **D,D-POC(7** + **8** + 9)*(**a**–**c**) and for **L,D-POC(7** + **8** + 9)* of our previous work^30^ (**d**–**f**). **a**, **d** Side views show dynamic stacking interaction between a linker triazole moiety (boxed grey in (**a**), boxed blue in (**d**)) and DNA triplex terminal bases. Terminal tyrosines are shown in stick representation. DNA atoms are shown in ball and stick representation. **b**, **e** Isolated views of the linker/base stacking interactions where the linker is shown in stick representation. **c**, **f** Top views along coiled-coil axis showing packing of the linkers and tyrosine side chains. Except for the tyrosines, coiled-coil atoms are not shown but collectively represented by the C-alpha atoms of the terminal tyrosines (red, blue, grey spheres with black outlines). White arrows indicated the tyrosine side chain which transiently separated one linker strand (grey) from the other two hydrophobically packed linker strands (blue and red) in the chosen snapshot.

The single MD snapshot containing representative features of the entire **D,D-POC(7** + **8** + 9)* further showed that the linkers packed against each other and against the oligonucleotide and peptide termini (Fig. 5a–c). These interactions were formed during the first half of the MD simulation and caused the simulation structure to bend and become more compact with time. In the last half of the simulation, an intramolecular stacking interaction persisted between a linker triazole moiety and a terminal thymine, as illustrated in Fig. 5a, b. The two other linkers associated closely with each other, as seen from the side (Fig. 5a) and top views along the coiled-coil axis (Fig. 5C). Similar interactions were present in the MD simulation of **L,D-POC(7** + **8** + 9)*^30^ (Fig. 5d–f), where two linkers associated by hydrophobic interactions, while the third linker was transiently separated from the two other linkers by the insertion of a tyrosine side chain (white arrow in Fig. 5d, f). Transient hydrogen bonds were also noticed between the N-terminal tyrosine phenolic OH groups and terminal oligonucleotide backbone phosphates. Overall, the linker regions of both **D,D-POC(7** + **8** + 9)* and its diastereomer **L,D-POC(7** + **8** + 9)*^30^ became compact due to the linker-nucleotide stacking, hydrophobic linker-linker interactions and linker-tyrosine interactions. Such a compact linker region limits the rotational freedom of individual linkers, allowing chiral communication between the DNA triplex and the trimeric peptide coiled-coil.

## Discussion

**Fig. 6:**
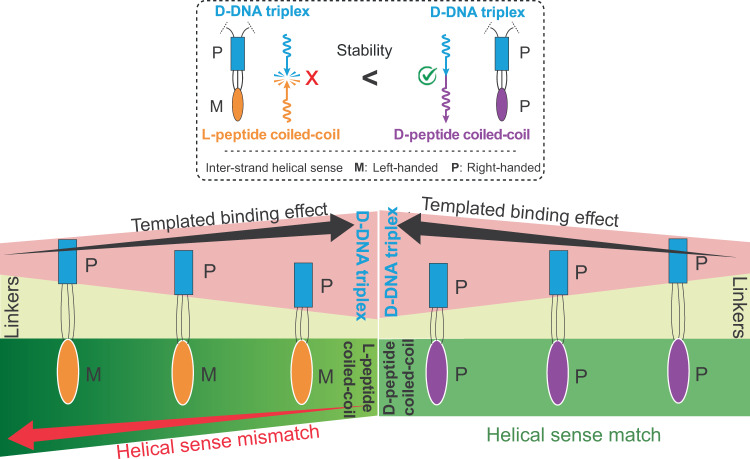
The stability of POC triple helices is influenced by the helical sense mismatch/match and the templated binding effect. D-DNA triplex is marked in cyan rectangle, L-peptide coiled-coil in orange ellipse and D-peptide coiled-coil in purple ellipse. M and P stand for left-handed and right-handed inter-strand helical senses, respectively. (Top: The clash symbol means the helical sense mismatch state, which may result in unfavourable energy cost and thus is marked in a red-cross. The no clash symbol represents the helical sense match state, which should not influence the stability and thus is marked in a green check).

All obtained results coherently supported our hypothesis that the chirality of supercoils can be transmitted between peptide coiled coils and DNA helices in a long-range manner. From SAXS and MD simulation, it is clear that no direct interaction exists between the trimeric peptide coiled-coil and DNA triplex domains. In addition, the three linkers have shown constrained conformations packed between two domains. Therefore, the chiral communication is very likely transmitted through these spatially constrained linkers in a manner similar to the transfer of superhelicity in torsionally constrained duplex DNA templates^41^. To the best of our knowledge, this is the first example of a chiral-to-chiral communication propagated between two biomacromolecular domains (Fig. 6). Our findings may explain the observation that opposite inter-strand helical senses rarely exist in proximity inside the same macromolecule due to unfavourable energy cost^22^.

Natural peptides can either assume left-handed or right-handed helices, which intertwine one another to form right-handed^42,43^ or left-handed screw sense^44–49^, respectively. Intriguingly, opposite coiled-coil helicities are only observed in proteins with the aid of either transition motifs (stutters) and/or the binding of other proteins^50^. For example, one research study demonstrated that a left-handed and a right-handed trimeric coiled-coil could simultaneously exist in the crystal structure of an immunoglobulin-binding protein D from *Escherichia coli*, where a 22-mer antiparallel β-sheet saddle (ca.15 nm) was inserted in ~120° rotation between the two coiled coils to circumvent the possible chirality clash^51^, which is far beyond the 4.5 nm limit found here. Another work on a mutated version of *Yersinia* YadA stalk domain (S299–L362) also showed that a 26-mer motif acted as the transition element between the opposite superhelices^52^. The helical sense mismatch effect unveiled in the present work lends support to the emergence of screw-sense homopairing (or homohelicity) between neighbouring macromolecular domains.

## Methods

### Synthesis of DNA constructs for single-molecule investigations

A polythymine (T90) containing oligonucleotide was taken and annealed with two POCs at the 5′- and 3′-end (Fig. 1). The annealed construct was phosphorylated to insert phosphate groups at the 5′-end of a POC. Then, it was ligated with two non-palindromic (1558 bp and 2424 bp in length) double-stranded DNA handles. The 1558 bp DNA handle was synthesized from PCR strategy by using a pBR322 plasmid as template, and 5′-biotin-TEG-GCA TTA GGA AGC CCA GTA GG and 5′-AAA CCA TAG AGG CTA CAC TAG AAG GAC AGT ATT TG-3′ as primers. BsaI-HFv2 enzyme was used to digest the purified PCR product to generate a sticky end compatible with the annealed fragment prepared above. For the 2424 bp dsDNA handles, we first prepared 2391 bp double-stranded DNA handles by PCR using the λ-DNA template with two primers, 5′-AAA AAA AAG AGC TCC TGA CGC TGG CAT TCG CAT CAA AG and 5′-AAA AAA AAG GTC TCG CCT GGT TGC GAG GCT TTG TGC TTC TC. Then, the purified PCR product was digested by SacI followed by labelling of digoxigenin-dUTP at the 3′-end SacI overhang by using terminal transferase enzyme for 8 h at 37 °C. To generate a sticky end, a BsaI enzyme was used to digest the purified digoxigenin labelled product. The purified BsaI digested handle and a 33 bp dsDNA adaptor were ligated by T4 DNA ligase to obtain the 2424 bp dsDNA handle with a sticky end that is compatible with the polyT containing fragment. Finally, three-piece ligation reactions were carried out between the polyT containing fragment and two non-palindromic handles by using T4 DNA ligase, and the final ligated DNA construct was stored in the −20 °C.

### Single-molecule force ramping experiments

A dual-beam laser-tweezers instrument was used to perform the single-molecule experiments^53^. All the experiments were performed at 23 °C in 10 mM MES buffer containing 100 mM KCl (pH 5.5). First, the DNA construct was incubated with digoxigenin-antibody-coated polystyrene beads (diameter 2.1 µm). Then, DNA constructs immobilized beads and streptavidin-coated (diameter 1.87 µm) beads were separately captured by two laser traps. Affinity linkages of biotin/streptavidin and digoxigenin-antibody/digoxigenin were used to tether the DNA construct between two beads by bringing two beads in contact with each other. One of the laser foci was fixed while the other was moveable by controlling the direction of the laser beam. When the two beads were moved apart, the DNA tether was stretched to generate tension on the coiled-coil peptide complex. The tension produced on the coiled-coil peptide complex was recorded in the force-extension (F-X) curve through LabView program (National Instruments, Austin, TX) at 1 KHz with loading rate of 5.5 pN/s (in the 10–30 pN range). Whether the tethered molecule was single or not was confirmed by the ~65 pN plateau in the F-X curve due to the melting of duplex DNA handles. The Savitzky-Golay function was used to filter the collected data at a time constant of 10 ms in the Matlab program (The MathWorks, Nattick, MA). The unfolding forces revealed in the F-X curves were used to determine the mechanical stability and the percentage of formation of coiled-coil peptide complexes.

### Change-in-contour-length (Δ*L*)

During the unfolding of coiled-coil peptide, the expected Δ*L* was calculated by the general Eq. (1),

1 $$\Delta L=L-x$$

where *L* is the contour length of the (T)_90_ fragment which can be determined as, *L* = *L*_nt_ × 90 nt = ~40.5 nm, here *L*_nt_ is the contour length of a single nucleotide, 0.40–0.45 nm/nt^54–56^, and *x* is the end-to-end distance, which is the sum of the diameter of dsDNA (~2 nm^57^) and length of the flanking (T)_2_ at each side of the (T)_90_ fragment (0.9 × 2 nm = 1.8 nm), i.e., 2 nm + 1.8 nm = 3.8 nm.

Therefore, the expected change-in-contour-length for a coiled-coil peptide complex is ~40.5 –3.8 = ~36.7 nm.

### Data analysis

At a force (*F*), the change in extension (Δ*x*) was calculated by the extension difference between the stretching and the relaxing traces at that force. Then, by using wormlike-chain (WLC) model (Eq. (2))^58,59^, the resulting Δ*x* value at this force was converted to the change-in-contour-length (Δ*L*) as follows,

2 $$\Delta x/\Delta L=1-1/2{({k}_{{{{{{\rm{B}}}}}}}T/FP)}^{1/2}+(F/S)$$

where *k*_B_ is the Boltzmann constant, *T* is absolute temperature, *P* is the persistent length of dsDNA (50.8 nm), and *S* is the stretching modulus (1243 pN)^60^.

### Calculation for relative contributions of chirality clash effect and templated binding effect

We assumed that the chirality clash effect led to the D-peptide/D-DNA system being favoured with respect to the L-peptide/D-DNA system. In the maximum templated effect for the L-peptide/D-DNA (defined as 100%), the percentage of triplex formation should be equivalent to that of the D-peptide/D-DNA system at each linker length. If there was a 100% chirality clash effect in the L-peptide/D-DNA, then its triplex population would be reduced to zero. By comparing the percentage formations of the two diastereoisomeric POC triplexes, the normalized chirality clash contribution for the L-peptide/D-DNA system at each linker length was determined by using Eq. (3).

3 $${{{{{\rm{Normalized}}}}}}\,{{{{{\rm{chirality}}}}}}\,{{{{{\rm{clash}}}}}}\,{{{{{\rm{contribution}}}}}}=(x-y)/x$$

where *x* and *y* are the triplex formation percentages of the D-peptide/D-DNA and L-peptide/D-DNA systems, respectively, at a particular length of the interdomain linker.

Then, the normalized templated binding contribution was determined as follows (Eq. (4)),

4 $${{{{{\rm{Normalized}}}}}}\,{{{{{\rm{templated}}}}}}\,{{{{{\rm{binding}}}}}}\,{{{{{\rm{contribution}}}}}}=1-{{{{{\rm{Normalized}}}}}}\,{{{{{\rm{chirality}}}}}}\,{{{{{\rm{clash}}}}}}\,{{{{{\rm{contribution}}}}}}$$

### Statistical data analysis

Data shown in Fig. 4B was obtained from different individual molecules. The data were presented as mean ± SD for 4 sets of independent samples. The statistical data analysis was performed by using two-sided unpaired *t*-tests.

### Reporting summary

Further information on research design is available in the Nature Research Reporting Summary linked to this article.

## Supplementary information

Supplementary Information

Description of Additional Supplementary Files

Supplementary Movie 1

Reporting Summary

## Data Availability

All data are available in the main text or the Supplementary Materials.
