# Supplementary material for: Chirality transmission in macromolecular domains
Source: Nat Commun. 2022 Jan 10;13:76. doi: 10.1038/s41467-021-27708-4 (PMC8748818; doi:10.1038/s41467-021-27708-4)
Supplement: Supplementary file 1 — Supplementary Information [file 41467_2021_27708_MOESM1_ESM.pdf]

S1 Supplementary Discussion.

For **ON1**-**ON2**, following the literature work[1], phosphoramidite monomers of 5′-deoxy-5′-amino-thymidine (**4**, four steps) and 5′-deoxy-5′-amino-adenosine (**13**, eight steps) were synthesized in our lab from thymidine and adenosine with minor alterations, respectively (Supplementary Fig. 1-2). A special solid support, 3′-amino-dT CPG (Glen Research), was used for the synthesis of **ON3**. γ-aminobutyric acid was conjugated to the 5′-deoxy-5′-amino-thymidine and 5′-deoxy-5′-amino-thymidine intermediates, which were then converted into the corresponding phosphoramidite monomers (**7**, four steps and **16**, four steps) for the synthesis of **ON4** and **ON5** (Supplementary Fig. 1-2). A new solid support (**23**) was developed in our lab for the synthesis of **ON6**, starting from 3′-deoxy-3′-azidothymidine in eight steps, providing a loading amount of 14.7 μmol/g (Supplementary Fig. 3). Four commercially available phosphoramidite monomers and one solid support, spacer phosphoramidite C3, spacer phosphoramidite 9, spacer phosphoramidite 18, 5′-amino-modifier 5 and 3'-PT-amino-modifier C6 PS, were used for the synthesis of remaining eleven ONs (**ON7**-**ON18**, see details in S3, Supplementary Information). 5′-Phosphate group of **ON9** was initially designed to facilitate ligation between the corresponding **POC9** with the DNA handle but was found to be problematic in ON synthesis (partial cleavage in ammonia treatment for **ON9** in the preparation of **D,D-POC9**). Therefore, no phosphate group was introduced on 5′-end for other ONs (**ON3**, **ON6**, **ON12**, **ON15** and **ON18**). Instead, a kinase[2] was employed to furnish a phosphate group to 5′-end of the corresponding POCs before ligation to the DNA handle.

S2 General Experimental

All basic chemical reagents used were purchased from Sigma-Aldrich and used without purification. Dichloromethane (DCM), *N*,*N*-diisopropylethylamine (DIPEA) and *N,N*-dimethylformamide (DMF) were dried over activated molecular sieve (3 Å, 8-12 mesh) and their dryness was determined on Karl Fischer titrator (< 15 ppm). All reactions were carried out under nitrogen or argon atmosphere using glassware that had been dried at 120 ˚C overnight. Column chromatography was carried out under pressure using Merck Millipore silica gel 60 (0.040-0.063 mm). Thin layer chromatography (TLC) was performed using Merck Kieselgel 60 F_{254} (0.22 mm thickness, aluminium backed). Compounds were visualized at 254 nm or stained with 10 % sulfuric acid in EtOH. IR spectra were measured on a Perkin Elmer Spectrum 65 FT-IR Spectrometer. ^{1}H-NMR spectra were measured at 400 MHz on a Bruker AVANCE III 400 spectrometer. ^{13}C-NMR spectra were measured at 101 MHz

on the same spectrometer. Chemical shifts are given in ppm and *J* values are given in Hz. All assignments for ^{1}H-NMR and ^{13}C-NMR have been confirmed by H-H COSY, HSQC and HMBC. ^{31}P-NMR spectra were recorded on a Bruker AVANCE III 400 spectrometer at 162 MHz. $CDCl_3$ and DMSO-d_6 were used as solvents. High resolution mass spectra were recorded in acetonitrile or methanol using the electrospray technique on a Bruker APEX III FT-ICR mass spectrometer. HPLC grade CH_3CN or methanol were used as the solvent. DNA phosphoramidite monomers, chemical phosphorylation Reagent II and solid chemical phosphorylation reagent II, 3'-PT-amino-modifier C6 PS, spacer phosphoramidite C3, spacer phosphoramidite 9, spacer phosphoramidite 18, 5'-amino-modifier 5, standard solid supports, 3'-Amino-dT-CPG, LCAA-CPG and additional reagents were purchased from Sigma-Aldrich, GE Healthcare, Glen Research or Link Technologies. BCN *N*-hydroxysuccinimide ester I was purchased from Berry&Associates. For all experiments, concentrations of ONs, ON-BCNs, and POCs were determined using theoretical values for ε_{260nm} of $1.11 \cdot 10^5$ $M^{-1} \cdot cm^{-1}$, $4.95 \cdot 10^5$ $M^{-1} \cdot cm^{-1}$ and $4.67 \cdot 10^5$ $M^{-1} \cdot cm^{-1}$ for **ON1/ON4/ON7/ON10/ON13/ON16 /ON1-BCN/ON4-BCN/ON7-BCN/ON10-BCN/ON13-BCN/ON16-BCN/POC1/POC4/POC7/POC10/POC13/POC16**, **ON2/ON5/ON8/ON11/ ON14/ON17/ON2-BCN/ON5-BCN/ON8-BCN/ON11-BCN/ON14-BCN/ON17-BCN/POC2/POC5/ POC8/POC11/POC14/POC17**, and **ON3/ON6/ON9/ON12/ ON15/ON18/ON3-BCN/ON6-BCN/ON9-BCN/ON12-BCN/ON15-BCN/ON18-BCN/POC3/POC6/ POC9/POC12/POC15/POC18**, respectively. A theoretical ε_{280nm} of $1.49 \cdot 10^3$ $M^{-1} \cdot cm^{-1}$ was used to establish the concentration of **L-azidopeptide** and **D-azidopeptide**.

For the preparation of DNA constructs in single-molecule force spectroscopy, DNA oligonucleotides were purchased from the Integrated DNA Technologies (IDT, IA) and enzymes were purchased from New England Biolab (NEB, England). Streptavidin and anti-digoxigenin coated polystyrene beads were purchased from Spherotech (Lake Forest, IL). If nothing else reported, all chemicals and solvents were purchased from Sigma Aldrich or Fisher Scientific and used without further purification.

S3 Synthesis of nucleoside phosphoramidite monomers and one solid support

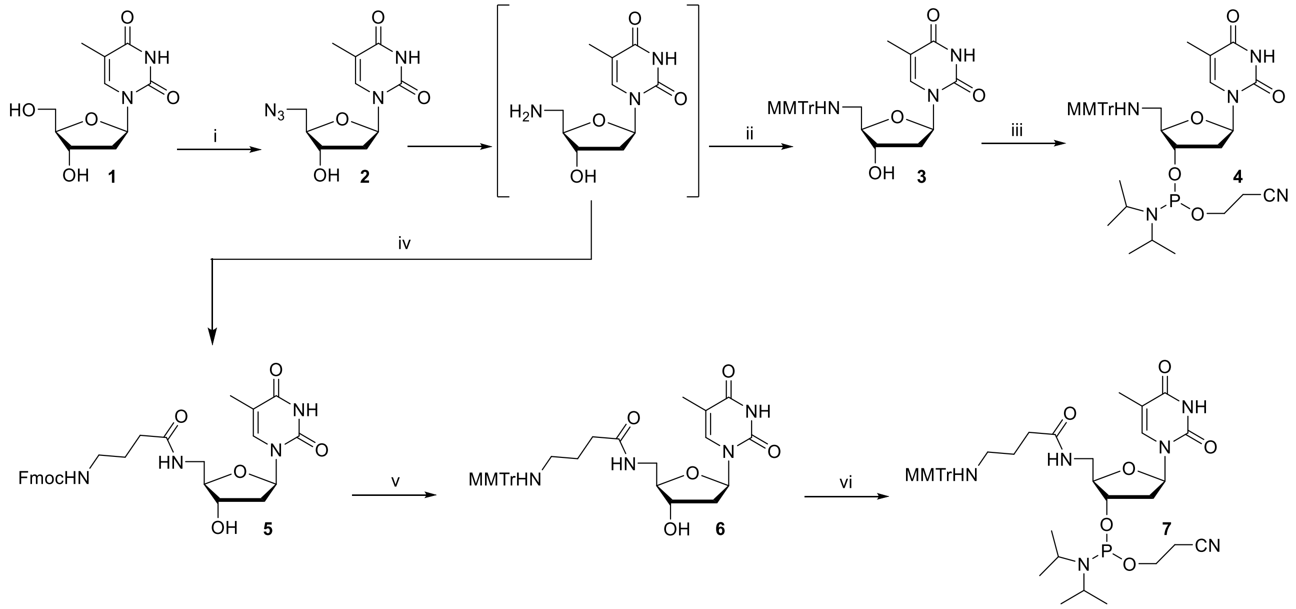

Supplementary Fig. 1. Synthesis of phosphoramidite **4** and **7**; i) PPh_3, NaN_3, CBr_4, DMF, rt, 24 h, 76%; ii) a) PPh_3, H_2O, pyridine, rt, 4h, b) 4-methoxytriphenyl chloride, rt, 1 h, 75% for two steps; iii) 2-cyanoethyl-*N*,*N*-diisopropylchlorophosphoramidite, DIPEA, CH_2Cl_2, rt, 1 h, 75%; iv) a) PPh_3, H_2O, pyridine, rt, 4 h, b) HATU, DIPEA, Fmoc-GABA-OH, DMF, rt, 2 h, 81% for two steps; v) a) piperidine, DMF, rt, 1 h, b) 4-methoxytriphenyl chloride, pyridine, rt, 5 h, 76% for two steps; vi) 2-cyanoethyl-*N*,*N*-diisopropylchlorophosphoramidite, DIPEA, CH_2Cl_2, rt, 2 h, 65%.

5'-Azido-5'-deoxythymidine (2)

PPh_3 (1.30 g, 4.91 mmol), NaN_3 (0.800 g, 12.3 mmol) and CBr_4 (1.62 g, 4.91 mmol) was added to a solution of thymidine (1.00 g, 4.15 mmol) in anhydrous DMF (25 mL). The reaction mixture was stirred at rt for 24 h. To this a $NaHCO_3$ solution (sat. aq., 50 mL) was added, and the resulting aqueous phase were extracted with CH_2Cl_2 (3x50 mL). The organic layer was combined and washed with H_2O (100 mL) and brine (2x100 mL). After drying with (Na_2SO_4) the solution was evaporated and the residue was purified by flash column chromatography on silica gel eluting with a stepwise gradient of MeOH in CH_2Cl_2 (0-5%) to afford a white foam (0.850 g, 76%). *Rf* = 0.32 (10% MeOH in CH_2Cl_2). V_{max} (KBr):2093 cm^{-1}. ^{1}H NMR (400 MHz, DMSO-d_6) δ ppm 11.33 (s, 1H, NH), 7.50 (d, *J* = 1.2 Hz, 1H, H6), 6.21 (t, *J* = 7.0 Hz, 1H, H1'), 5.41 (d, *J* = 4.4 Hz, 1H, 3'-OH), 4.26 – 4.15 (m, 1H, H3'), 3.89 – 3.80 (m, 1H, H4'), 3.56 (d, *J* = 5.3 Hz, 2H, H5', H5''), 2.30 – 2.05 (m, 2H, H2', H2''), 1.80 (s, 1H, CH_3). ^{13}C NMR (101 MHz, DMSO-d_6) δ ppm 163.60 (s, C4), 150.43 (s, C2), 136.01 (s, C6), 109.76 (s, C5), 84.51 (s, C4'), 83.86 (s, C1'), 70.70 (s, C3'), 51.69 (s, C5'), 38.08 (s, C2'), 12.03 (s, CH_3). Data is consistent with published NMR data.[3] HRMS (ESI) m/z calc. for $C_{10}H_{14}N_5O_4Na$ $[M+Na]^+$ 290.0859, found 290.0867; m/z calc. for $C_{10}H_{14}N_5O_4$ $[M+H]^+$ 268.1040, found 268.1048.

5'-*N*-(4-Methoxytrityl)amino-5'-deoxythymidine (3)

PPh_3 (600 mg, 2.25 mmol) was added to a solution of **2** (400 mg, 1.49 mmol) in anhydrous pyridine (6 mL). The reaction mixture was stirred at rt for 2 h. H_2O (2 mL) was added and the resulting solution was further stirred at rt for 2 h. The mixture was diluted with H_2O (20 mL), the precipitates were filtered off (PPh_3 and $Ph_3P{=}O$) and the filtrate was rinsed with EtOAc (3x15 mL). The aqueous solution was evaporated to afford a white solid, which was used for the next step without further purification. The white powder was dissolved in anhydrous pyridine (10 mL) under argon atmosphere before 4-methoxytriphenylmethyl chloride (630 mg, 2.05 mmol) was added. The reaction mixture was stirred at rt for 19 h before the reaction was quenched with EtOH (3 mL). The solvents were removed under reduced pressure and the residue was dissolved in CH_2Cl_2 (40 mL) and washed with H_2O (2x50 mL). The organic layer was evaporated, and the residue was purified by flash column chromatography on silica gel eluting with a stepwise gradient of MeOH in CH_2Cl_2 (0-3%) to furnish the final product as a white foam (570 mg, 75%). *Rf* = 0.23 (5% MeOH in CH_2Cl_2). ^{1}H NMR (400 MHz, DMSO-d_6) δ ppm 11.31 (s, 1H, NH), 7.44 – 7.39 (m, 5H, MMTr), 7.32 – 7.24 (m, 6H, MMTr), 7.18 (t, *J* = 7.3 Hz, 2H, MMTr), 6.85 (d, *J* = 9.0 Hz, 2H, Ortho to OCH_3), 6.15 (t, *J* = 6.8 Hz, 1H, H1'), 5.23 (d, *J* = 4.7 Hz, 1H, 3'-OH), 4.24 – 4.15 (m, 1H, H3'), 3.85 – 3.79 (m, 1H, H4'), 3.73 (s, 3H, OCH_3), 2.65 (t, *J* = 8.1 Hz, 1H, 5'-NH), 2.36 – 2.03 (m, 4H, H2', H2'', H5', H5''), 1.69 (d, *J* = 0.9 Hz, 3H, CH_3). ^{13}C NMR (101 MHz, DMSO-d_6) δ ppm 163.63 (s, C4), 157.38 (s, MMTr), 150.37 (s, C2), 146.23 (s, MMTr), 146.21 (s, MMTr), 137.73 (s, MMTr), 135.90 (s, C6), 129.58 (s, MMTr), 128.25 (s, MMTr), 127.66 (s, MMTr), 126.01 (s, MMTr), 113.02 (s, MMTr), 109.53 (s, MMTr), 85.74 (s, C4'), 83.44 (s, C1'), 71.11 (s, MMTr), 69.74 (s, C3'), 54.89 (s, MMTr [OCH_3]), 46.07 (s, C5'), 38.81 (s, C2'), 12.08 (s, CH_3). Data is consistent with published NMR data.[3] HRMS (ESI) m/z calc. for $C_{30}H_{32}N_3O_5Na$ $[M+Na]^+$ 536.2155, found 536.2134; m/z calc. for $C_{30}H_{32}N_3O_5$ $[M+H]^+$ 514.2336, found 514.2321.

5'-*N*-(4-Methoxytrityl)amino-5'-deoxythymidine-3'-*O*-(2-cyanoethyl-*N,N*-diisopropylamino)-phosphite (4)

To a stirred solution of **3** (530 mg, 1.03 mmol) in anhydrous CH_2Cl_3 (15 mL) was added DIPEA (0.276 mL, 1.55 mmol) and 2-Cyanoethyl *N*,*N*-diisopropylchlorophosphoramidite (0.276 mL, 1.24 mmol). The reaction mixture was stirred at rt for 1 h under argon atmosphere before a KCl solution (sat. aq., 6 mL, purged with argon) was added. The resulting mixture was further stirred for 10 minutes before the organic layer was separated and evaporated *in vacuo*. The residue was purified on flash column chromatography on silica gel (neutralized by 2% Et_3N) eluting with EtOAc/Petroleum ether (1:2 V/V, purged with argon). The residue was taken up in a minimum CH_2Cl_2 and precipitated into

cold n-hexane. The white precipitate was collected and co-evaporated with anhydrous CH_2Cl_2 to furnish the final product as a white foam (560 mg, 75%). *Rf* = 0.65 (5% MeOH IN CH_2Cl_2). ^{31}P NMR (162 MHz, $CDCl_3$) δ ppm 149.14 (s), 148.89 (s). Data is consistent with published NMR data.[3] HRMS (ESI) m/z calc. for $C_{39}H_{48}N_5O_6PNa$ $[M+Na]^+$ 736.3234, found 736.3248; m/z calc. for $C_{39}H_{49}N_5O_6P$ $[M+H]^+$ 714.3415, found 714.3419.

5'-*N*-(4-Fmoc-amino-1-oxobutyl)amino-5'-deoxythymidine (5)

To a stirred solution of **2** (520 mg, 1.94 mmol) and anhydrous pyridine (6 mL) was added PPh_3 (763 mg, 2.91 mmol). The reaction mixture was stirred at rt for 2 h. H_2O (3 mL) was added and the resulting solution was further stirred at rt for 2 h. The reaction mixture was diluted with H_2O (24 mL), the precipitates were filtered off (PPh_3 and Ph_3P=O), and the filtrate was rinsed with EtOAc (3x20 mL). The aqueous solution was evaporated to afford a white solid, which was used for the next step without further purification. The white solid was dissolved in anhydrous DMF (10 mL), which was then added into a solution containing Fmoc-GABA-OH (890 mg, 2.74 mmol), DIPEA (1.80 mL, 10.3 mmol) and HATU (999 mg, 2.74 mmol) in anhydrous DMF (15 mL). The reaction mixture was stirred at rt for 2 h before CH_2Cl_2 (100 mL) and H_2O (100 mL) were added. The organic layer was separated, and the aqueous layer was further extracted with CH_2Cl_2 (2x50 mL). All the organic phases were combined and washed with H_2O (50 mL) and brine (50 mL). White precipitate started to appear in the organic phase, to which celite was added to load the crude product. The suspension was evaporated *in vacuo* to remove solvents, the solid residue was added onto the top of the silica gel column and purified by flash column chromatography eluting with a stepwise gradient of MeOH in CH_2Cl_2 (0-5%) to furnish the final product as a white solid (860 mg, 81%). *Rf* = 0.35 (10% MeOH in CH_2Cl_2). ^1H NMR (400 MHz, DMSO-d_6) δ ppm 11.31 (s, 1H, NH), 8.11 (t, *J* = 5.7 Hz, 1H, NH), 7.88 (d, *J* = 7.5 Hz, 2H, Fmoc-Ar), 7.68 (d, *J* = 7.4 Hz, 2H, Fmoc-Ar), 7.50 (d, *J* = 1.0 Hz, 1H, H6), 7.43 – 7.37 (m, 2H, Fmoc-Ar), 7.37 – 7.29 (m, 3H, Fmoc-Ar, NH), 6.13 (t, *J* = 7.0 Hz, 1H, H1'), 5.38 (d, *J* = 4.1 Hz, 1H, 3'-OH), 4.32 – 4.24 (m, 2H, CH_2 [Fmoc]), 4.24 – 4.12 (m, 2H, H3', CH-[Fmoc]), 3.74 (t, *J* = 7.3 Hz, 1H, H4'), 3.35 – 3.19 (m, 2H, H5', H5''), 3.01 – 2.90 (m, 2H, CH_2), 2.15 – 2.01 (m, 4H, H2', H2'', CH_2), 1.80 (s, 3H, CH_3), 1.67 – 1.59 (m, 2H, CH_2). ^{13}C NMR (101 MHz, DMSO-d_6) δ ppm 172.06 (s, C=O), 163.70 (s, C4), 156.06 (s, C=O), 150.42 (s, C2), 143.90 (s), 140.69 (s, Fmoc-Ar), 136.08 (s, C6), 127.55 (s, Fmoc-Ar), 127.01 (s, Fmoc-Ar), 125.12 (s, Fmoc-Ar), 120.06 (s, Fmoc-Ar), 109.66 (s, C5), 84.93 (s, C4'), 83.83 (s, C1'), 71.21 (s, C3'), 65.21 (s, CH_2 [Fmoc]), 46.74 (s, CH [Fmoc]), 40.86 (s, C5'), 40.15 (s, CH_2), 38.39 (s, C2'), 32.68 (s, CH_2), 25.68 (s, CH_2), 11.99 (s, CH_3). HRMS (ESI) m/z calc. for

$C_{29}H_{32}N_4O_7Na$ $[M+Na]^+$ 571.2163, found 571.2189; m/z calc. for $C_{29}H_{33}N_4O_7$ $[M+H]^+$ 549.2343, found 549.2378.

5'-*N*-(4-Methoxytrityl-amino-1-oxobutyl)amino-5'-deoxythymidine (6)

Piperidine (1.50 mL, 15.2 mmol) was added into a solution of **5** (600 mg, 1.09 mmol) in anhydrous DMF (10 mL). The reaction mixture was stirred at rt for 1 h. After all the solvents were removed *in vacuo*, the residue was partitioned between diethyl ether/H_2O (80 mL/80 mL). The aqueous phase was separated and concentrated under nitrogen atmosphere. The residue was co-evaporated with anhydrous acetonitrile for three times to give an off-white solid that was kept under vacuo overnight. The solid was dissolved in anhydrous pyridine (15 mL) and stirred under argon atmosphere before 4-methoxytriphenylmethyl chloride (650 mg, 2.11 mmol) was added. The resulting solution was stirred at rt for 5 h before EtOH (5 mL) was added to quench the reaction. After the solvents were evaporated *in vacuo*, the residue was redissolved in MeOH. To this celite was added to load the crude product. The suspension was evaporated *in vacuo* to remove solvents, the solid residue was added onto the top of the silica gel column and purified by flash column chromatography on silica gel eluting with a stepwise gradient of MeOH in CH_2Cl_2 (0-10%) to furnish the final product as a white foam (500 mg, 76%). *Rf* = 0.30 (10% MeOH in CH_2Cl_2). ^{1}H NMR (400 MHz, $CDCl_3$) δ ppm 7.48 – 7.38 (m, 4H, NH, MMTr-Ar), 7.36 – 7.28 (m, 2H, MMTr-Ar), 7.26 – 7.18 (m, 4H, MMTr-Ar), 7.18 – 7.10 (m, 2H, MMTr-Ar), 7.04 (d, 1H, H6), 6.88 – 6.80 (m, 1H, NH), 6.81 – 6.74 (m, 2H, MMTr-Ar), 6.13 – 5.98 (m, 1H, H1'), 4.26 – 4.16 (m, 1H, H3'), 3.96 – 3.84 (m, 1H, H4'), 3.74 (s, 3H, OCH_3), 3.63 – 3.41 (m, 2H, H5', H5''), 2.34 – 2.24 (m, 4H, CH_2, H2', H2''), 2.19 – 2.03 (m, 4H, CH_3, 3'-OH), 1.86 – 1.75 (m, 5H, CH_2, CH_2, NH). ^{13}C NMR (101 MHz, $CDCl_3$) δ ppm 174.38 (s, C=O), 163.64 (s, C4), 157.86 (s, C=O), 150.49 (s, C2), 146.31 (s, MMTr-Ar), 138.21 (s, MMTr-Ar), 136.65 (s, C6), 129.80 (s, MMTr-Ar), 128.53 (s, MMTr-Ar), 127.78 (s, MMTr-Ar), 126.24 (s, MMTr-Ar), 113.13 (s, MMTr-Ar), 111.37 (s, C5), 86.35 (s, C1'), 85.15 (s, C4'), 71.67 (s, C3'), 70.39 (s, MMTr), 55.21 (s, MMTr [OCH_3]), 43.09 (s, CH_2), 41.02 (s, C5'), 38.77 (s, C2'), 34.48 (s, CH_2),26.64 (s, CH_2), 12.45 (s, CH_3). HRMS (ESI) m/z calc. for $C_{34}H_{39}N_4O_6$ $[M+H]^+$ 599.2864, found 599.2894.

5'-*N*-(4-Methoxytrityl-amino-1-oxobutyl)amino-5'-deoxythymidine-3'-*O*-(2-cyanoethyl-*N*,*N*-diisopropyl-amino)phosphite (7)

2-Cyanoethyl *N*,*N*-diisopropylchlorophosphoramidite (0.170 mL, 0.761 mmol) was added to a solution of **6** (360 mg, 0.600 mmol) and DIPEA (0.170 mL, 0.951 mmol) in anhydrous CH_2Cl_2 (15 mL).

The reaction mixture was stirred at rt for 1 h under argon atmosphere before KCl solution (sat. aq., 8 mL, purged with argon) was added. The organic phase was separated, and the solvents were removed *in vacuo*. The residue was purified by flash column chromatography on silica gel eluting with MeOH in CH_2Cl_2 (0-1.5%, solvents purged with argon) to yield the diastereomeric product, which was further precipitated in CH_2Cl_2/*n*-hexane system twice to furnish the final product as a white foam (310 mg, 65%). *Rf* = 0.68 (10% MeOH in CH_2Cl_2). ^{31}P NMR (162 MHz, $CDCl_3$) δ ppm 149.44 (s), 148.76 (s). HRMS (ESI) m/z calc. for $C_{43}H_{56}N_6O_7P$ $[M+H]^+$ 799.3942, found 799.3911.

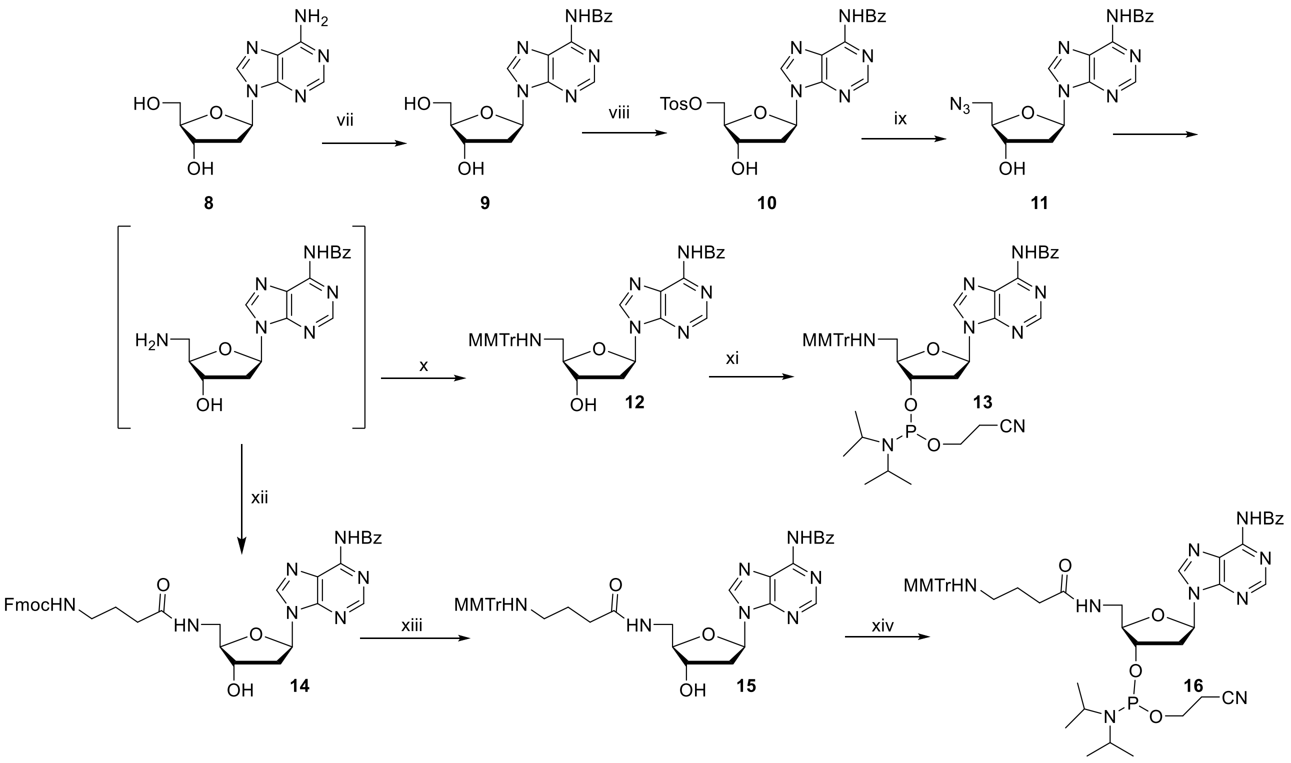

Supplementary Fig. 2. Synthesis of phosphoramidite **13** and **16**; vii) a) chlorotrimethylsilane, pyridine, 0 °C, 0.5 h, b) benzoyl chloride, rt, 2 h, c) 28% aq. ammonia, rt, 0.5 h, 94% for three steps; viii) 4-methylbenzenesulfonylchloride, pyridine, rt, 0.75 h, 77%; ix) NaN_3, DMF, 88 °C, 4 h, 82%; x) a) PPh_3, pyridine, H_2O, rt, 3.5 h, b) 4-methoxytriphenyl chloride, rt, 19 h, 83% for two steps; xi) 2-cyanoethyl *N*,*N*-diisopropylchlorophosphoramidite, DIPEA, CH_2Cl_2, rt, 1 h, 78%; xii) PPh_3, pyridine, H_2O, rt, 4 h, b) HATU, DIPEA, Fmoc-GABA-OH, DMF, rt, 2 h, 74% for two steps; xiii) a) piperidine, DMF, rt, b) 4-methoxytriphenyl chloride, pyridine, rt, 7 h, 83% for two steps; xiv) 2-cyanoethyl *N*,*N*-diisopropylchloro-phosphoramidite, DIPEA, CH_2Cl_2, rt, 1 h, 97%.

*N^6^*-Benzoyl-2′-deoxyadenosine (9)

Chlorotrimethylsilane (2.80 mL, 21.9 mmol) was added dropwise to a solution of 2′-deoxyadenosine (1.10 g, 4.37 mmol) in anhydrous pyridine (20 mL) at 0 °C under argon atmosphere. The resulting solution was stirred at 0 °C for 30 min. To this benzoyl chloride (2.41 mL, 20.75 mmol) was added. The reaction mixture was increased to rt and stirred for additional 2 h. The mixture was again cooled to 0 °C before H_2O (8 mL) was added. The resulting solution was stirred for 5 min and aq. ammonia

(12 mL, 28%) was added. The reaction mixture was warmed to rt and stirred for 30 minutes. The solvents were removed under reduced pressure and the residue was dissolved in H_2O (60 mL). The aqueous phase was washed with EtOAc (2x40 mL) and put under N_2-flow until white precipitate started to form. The N_2-flow was stopped, and the aqueous phase was kept at 4 ^oC for 2 h before the precipitate was filtered off and dried under reduced pressure to furnish the final product as a white solid (1.46g, 94%). *Rf* = 0.27 (10% MeOH in CH_2Cl_2). ^1H NMR (400 MHz, DMSO-d_6) δ ppm 10.05 (s, 1H, NH), 8.74 (s, 1H, H2), 8.68 (s, 1H, H8), 8.07 – 8.01 (m, 2H, Bz-Ar), 7.67 – 7.60 (m, 1H, Bz-Ar), 7.59 – 7.50 (m, 2H, Bz-Ar), 6.48 (t, *J* = 6.8 Hz, 1H, H1'), 5.37 (s, 1H, 3'-OH), 5.02 (s, 1H, 5'-OH), 4.46 (s, 1H, H3'), 3.94 – 3.88 (m, 1H, H4'), 3.68 – 3.50 (m, 2H, H5'), 2.85 – 2.75 (m, 1H, H2''), 2.41 – 2.31 (m, 1H, H2'). ^{13}C NMR (101 MHz, DMSO-d_6) δ ppm 165.68 (s, C=O), 151.87 (s, C2), 151.43 (s, C6), 150.30 (s, C4), 143.00 (s, C8), 133.40 (s, Bz-Ar), 132.37 (s, Bz-Ar), 128.42 (s, Bz-Ar), 125.86 (s, C5), 87.97 (s, C4'), 83.72 (s, C1'), 70.67 (s, C3'), 61.59 (s, C5'), 40.15 (s, C2'). Data is consistent with published NMR data.[4] HRMS (ESI) m/z calc. for $C_{18}H_{18}N_5O_4$ $[M+H]^+$ 356.1353, found 356.1337.

5'-*O*-(4-Methylbenzenesulfonyl)-*N*^{6}-benzoyl-2'-deoxyadenosine (10)

4-Methylbenzenesulfonylchloride (2.30 g, 12.1 mmol) was added to a solution of **9** (1.40 g, 4.05 mmol) in anhydrous pyridine (15 mL). The reaction mixture was stirred at rt for 45 min under argon atmosphere. The reaction mixture was cooled to 0 ^oC before H_2O (5.5 mL) was added to quench the reaction. The resulting solution was stirred for 15 min before the solvent was removed under reduced pressure. The residue was dissolved in EtOAc (150 mL) and washed with 5% NaHCO3 sol. (2x80 mL), H2O (50 mL) and Brine (2x80 mL), dried (Na2SO4) and evaporated. The residue was purified on flash column chromatography on silica gel eluting with a stepwise gradient of MeOH in CH_2Cl_2 (0-6%) to furnish the final product as a white foam (1.54 g, 77%). *Rf* = 0.32 (10% MeOH in CH_2Cl_2). ^1H NMR (400 MHz, DMSO-d_6) δ ppm 11.23 (s, 1H, NH), 8.68 (s, 1H, H2), 8.55 (s, 1H, H8), 8.12 – 8.03 (m, 2H, Bz-Ar), 7.73 – 7.51 (m, 5H, Bz-Ar, Tos-Ar), 7.31 (d, *J* = 8.0 Hz, 2H, Tos-Ar), 6.45 (t, *J* = 6.7 Hz, 1H, H1'), 5.58 (d, *J* = 4.1 Hz, 1H, 3'-OH), 4.56 – 4.44 (m, 1H, H3'), 4.37 – 4.20 (m, 2H, H5', H5''), 4.07 – 3.99 (m, 1H, H4'), 2.90-2.84 (m, 1H, H2'), 2.42 – 2.35 (m, 1H, H2''), 2.33 (s, 3H, CH_3 [Tos]). ^{13}C NMR (101 MHz, DMSO-d_6) δ ppm 165.59 (s, C=O), 151.70 (s, C2), 151.45 (s, C6), 150.37 (s, C4), 144.91 (s, C8), 143.20 (s, Tos-Ar), 133.37 (s, Bz-Ar), 132.40 (s, Bz-Ar), 131.99 (s, Tos-Ar), 129.88 (s, Tos-Ar), 128.46 (s, Bz-Ar), 128.43 (s, Tos-Ar), 127.45 (s, Tos-Ar), 125.91 (s, C5), 83.97 (s, C4'), 83.79 (s, C1'), 70.24 (s, C5'), 70.18 (s, C3'), 38.11 (s, C2'), 20.99 (s, CH_3 [Tos]). Data is consistent with published NMR data.[3] HRMS (ESI) m/z calc. for $C_{24}H_{23}N_5O_6S$ Na $[M+Na]^+$ 532.1261, found 532.1240; m/z calc. for $C_{24}H_{24}N_5O_6S$ $[M+H]^+$ 510.1441, found 510.1422.

5'-Azido-*N*[6]-benzoyl-2',5'-dideoxyadenosine (11)

NaN_3 (0.958 g, 14.7 mmol) was added to a stirred solution containing **10** (1.50 g, 2.94 mmol) in anhydrous DMF (20 mL). The reaction mixture was heated to 88 ^oC and stirred at the same temperature for 4 hours under argon atmosphere. After cooling to room temperature, the reaction mixture was diluted with CH_2Cl_2 (80 mL) and the organic layer was washed with H_2O (3x20 mL), dried (Na_2SO_4) and evaporated. The residue was purified by flash column chromatography on silica gel eluting with a stepwise gradient of MeOH in CH_2Cl_2 (0-10%) to furnish the final product as a white foam (0.910 g, 82%). *Rf* = 0.41 (10% MeOH in CH_2Cl_2). V_{max} (KBr): 2096 cm^{-1}. ^1H NMR (400 MHz, DMSO-d_6) δ ppm 11.19 (s, 1H, NH), 8.77 (s, 1H, H2), 8.69 (s, 1H, H8), 8.10 – 7.97 (m, 2H, Bz-Ar), 7.70 – 7.61 (m, 1H, Bz-Ar), 7.59 – 7.46 (m, 2H, Bz-Ar), 6.53 (t, *J* = 6.8 Hz, 1H, H1'), 5.54 (d, *J* = 4.2 Hz, 1H, 3'-OH), 4.54 – 4.43 (m, 1H, H3'), 4.07 – 3.96 (m, 1H, H4'), 3.74 – 3.46 (m, 2H, H5'), 3.05 – 2.91 (m, 1H, H2'), 2.46 – 2.36 (m, 1H, H2''). ^{13}C NMR (101 MHz, DMSO-d_6) δ ppm 165.54 (s, C=O), 151.92 (s, C2), 151.58 (s, C6), 150.34 (s, C4), 143.31 (s, C8), 133.35 (s, Bz-Ar), 132.37 (s, Bz-Ar), 128.41 (s, Bz-Ar), 125.93 (s, C5), 85.52 (s, C4'), 83.70 (s, C1'), 71.05 (s, C3'), 51.66 (s, C5'), 38.16 (s, C2'). Data is consistent with published NMR data.[3] HRMS (ESI) m/z calc. for $C_{17}H_{16}N_8O_3Na$ $[M+Na]^+$ 403.1237, found 403.1226; m/z calc. for $C_{17}H_{17}N_8O_3$ $[M+H]^+$ 381.1418, found 381.1418.

5'-*N*-(4-Methoxytrityl)amino-*N*[6]-benzoy-2',5'-dideoxyadenosine (12)

PPh_3 (315 mg, 1.20 mmol) was added to a solution of **11** (310 mg, 0.800 mmol) in anhydrous pyridine (8 mL). The reaction mixture was stirred at rt for 1.5 h before H_2O (4 mL) was added. The resulting solution was further stirred at rt for 2 h before all solvents were removed. The residue was partitioned between EtOAc/H_2O (50 mL/50 mL). The aqueous phase was separated, and the organic layer was further extracted with H_2O (20 mL). The aqueous phases were combined and washed with CH_2Cl_2 (50 mL) before the aqueous phase was concentrated under N_2-flow. The residue was co-evaporated with anhydrous acetonitrile for three times to give a white foam that was kept under vacuo overnight. The white foam was dissolved in anhydrous pyridine (12 mL), to which 4-methoxytriphenylmethyl chloride (650 mg, 2.10 mmol) was added. The reaction mixture was stirred at rt for 19 h before EtOH (1.5 mL) was added to quench the reaction. To this CH_2Cl_2 (80 mL) was added. The organic phase was washed with H_2O (2x30 mL) and evaporated *in vacuo*. The residue was purified on flash column chromatography on silica gel eluting with MeOH in CH_2Cl_2 (0-6%) to furnish the final product as a white foam (440 mg, 83%). *Rf* = 0.5 (10% MeOH in CH_2Cl_2).^1H NMR

(400 MHz, DMSO-d_6) δ ppm 11.17 (s, 1H, NH), 8.57 (s, 1H, H2), 8.18 (s, 1H, H8), 8.05 (d, *J* = 7.3 Hz, 2H, Bz-Ar), 7.69 – 7.61 (m, 1H, Bz-Ar), 7.59 – 7.52 (m, 2H, Bz-Ar), 7.40 (d, *J* = 7.9 Hz, 4H, MMTr-Ar), 7.32 – 7.22 (m, 6H, MMTr-Ar), 7.21 – 7.11 (m, 2H, MMTr-Ar), 6.82 (d, *J* = 8.9 Hz, 2H, MMTr-Ar), 6.42 (t, *J* = 6.8 Hz, 1H, H1'), 5.40 (d, *J* = 4.1 Hz, 1H, 3'-OH), 4.73 – 4.61 (m, 1H, H3'), 4.11 – 3.98 (m, 1H, H4'), 3.71 (s, 3H, OCH_3), 3.14 – 2.97 (m, 2H, H2', NH), 2.40 – 2.22 (m, 3H, H2', H5', H5'').^{13}C NMR (101 MHz, DMSO-d_6) δ ppm 165.52 (s, C=O), 157.34 (s, MMTr-Ar), 151.70 (s, C2), 151.05 (s, C6), 150.37 (s, C4), 146.25 (s, MMTr-Ar), 146.16 (s, MMTr-Ar), 143.80 (s, C8), 137.72 (s, MMTr-Ar), 133.37 (s, Bz-Ar), 132.37 (s, Bz-Ar), 129.56 (s, MMTr-Ar), 128.41 (s, Bz-Ar), 128.26 (s, MMTr-Ar), 127.63 (s, MMTr-Ar), 126.20 (s, MMTr-Ar), 125.96 (s, C5), 113.00 (s, MMTr-Ar), 86.89 (s, C4'), 84.31 (s, C1'), 71.61 (s, C3'), 69.68 (s, MMTr), 54.91 (s, MMTr [OCH_3]), 45.73 (s, C5'), 38.72 (s, C2'). Data is consistent with published NMR data.[3] HRMS (ESI) m/z calc. for $C_{37}H_{34}N_6O_4Na$ $[M+Na]^+$ 649.2533, found 649.2506; m/z calc. for $C_{37}H_{35}N_6O_4$ $[M+H]^+$ 627.2714, found 627.2686.

5'-*N*-(4-Methoxytrityl)amino-*N*^{6}-benzoy-2',5'-dideoxyadenosine-3'-*O*-(2-cyanoethyl,*N*,*N*-di-isopropylamino)phosphite (13)

2-Cyanoethyl *N*,*N*-diisopropylchlorophosphoramidite (0.135 mL, 0.622 mmol) was added to a solution of **12** (300 mg, 0.479 mmol) and DIPEA (0.135 mL, 0.777 mmol) in anhydrous CH_2Cl_2 (12 mL). The reaction was stirred at rt for 1 h under argon atmosphere. To this KCl solution (sat. aq., 6 mL, purged with argon) was added. The organic phase was separated and washed with H_2O (6 mL, purged with argon), dried (Na_2SO_4) and evaporated. The residue was precipitated in CH_2Cl_2/*n*-hexane twice to furnish the final product as a white foam (300 mg, 78%). *Rf* = 0.3 (EtOAc:petroleum ether; 2:1). ^{31}P NMR (162 MHz, $CDCl_3$) δ ppm 148.70 (s), 148.50 (s). Data is consistent with published NMR data.[3] HRMS (ESI) m/z calc. for $C_{46}H_{51}N_8O_5Na$ $[M+Na]^+$ 849.3612, found 849.3582; m/z calc. for $C_{46}H_{52}N_8O_5$ $[M+H]^+$ 827.3792, found 827.3774.

5'-*N*-(4-Fmoc-amino-1-oxobutyl)amino-*N*^{6}-benzoyl-2',5'-dideoxyadenosine (14)

PPh_3 (770 mg, 2.92 mmol) was added to a solution of **11** (700 mg, 1.84 mmol) in anhydrous pyridine (6 mL). The resulting solution was stirred at rt for 2 h. After adding H_2O (4 mL), the reaction mixture was further stirred at rt for 2 h. To this H_2O (26 mL) was added, the resulting precipitate was filtered off (PPh_3 and Ph_3P=O) and the filtrate was rinsed with EtOAc (3x25 mL). The aqueous solution was evaporated to afford a white solid. The white solid was dissolved in anhydrous DMF (12 mL), which was added dropwise to a solution of Fmoc-GABA-OH (890 mg, 2.74 mmol), DIPEA (1.80 mL, 10.3 mmol) and HATU (999 mg, 2.74 mmol) in anhydrous DMF (15 mL). The reaction mixture was stirred

at rt for 2 h before CH_2Cl_2 (120 mL) and H_2O (120 mL) were added. The organic phase was separated, and the aqueous layer was further extracted with CH_2Cl_2 (2x60 mL). All the organic phases were combined and washed with brine (60 mL), H_2O (60 mL) and concentrated under reduced pressure. The residue was dissolved in CH_2Cl_2/MeOH (40 mL/10 mL) and Celite powder was added to the solution. The solvents were removed under reduced pressure and the resulting crude solid was loaded onto the silica column. The product was then isolated by flash column chromatography on silica gel eluting with a stepwise gradient of MeOH in CH_2Cl_2 (0-7%) to furnish the final product as a white solid (900 mg, 74%). *Rf* = 0.33 (10% MeOH in CH_2Cl_2). ^1H NMR (400 MHz, DMSO-d_6) δ ppm 11.21 (s, 1H, NH), 8.77 (s, 1H, H2), 8.69 (s, 1H, H8), 8.09 – 7.98 (m, 3H, Bz-Ar, NH), 7.92 – 7.82 (m, 2H, NH, Fmoc-Ar), 7.72 – 7.60 (m, 3H, Bz-Ar, Fmoc-Ar, NH), 7.59 – 7.51 (m, 2H, Bz-Ar), 7.45 – 7.36 (m, 2H, Fmoc-Ar), 7.36 – 7.26 (m, 3H, Fmoc-Ar), 6.52 – 6.41 (m, 1H, H1'), 5.42 (d, *J* = 3.9 Hz, 1H, 3'-OH), 4.44 – 4.34 (m, 1H, H3'), 4.34 – 4.24 (m, 2H, CH_2[Fmoc]), 4.25 – 4.15 (m, 1H, CH[Fmoc]), 3.97 – 3.85 (m, 1H, H4'), 3.51 – 3.22 (m, 2H, H5', H5''), 3.05 – 2.94 (m, 2H, CH_2), 2.94 – 2.83 (m, 1H, H2'), 2.41 – 2.30 (m, 1H, H2''), 2.17 – 2.02 (m, 2H, CH_2), 1.70 – 1.54 (m, 2H, CH_2). ^{13}C NMR (101 MHz, DMSO-d_6) δ ppm 174.14 (s, C=O), 171.98 (s, C=O), 165.72 (s, C=O), 156.07 (s, C2), 151.89 (s, C6), 151.48 (s, C4), 150.39 (s, Fmoc-Ar), 143.89 (s, Fmoc-Ar), 143.44 (s, C8), 140.69 (s, Fmoc-Ar), 133.38 (s, Bz-Ar), 132.39 (s, Bz-Ar), 128.44 (s-Fmoc-Ar), 128.42 (Bz-Ar, Fmoc-Ar), 127.53 (s, Fmoc-Ar), 127.00 (s, Fmoc-Ar), 126.09 (s, C5), 125.09 (s, Fmoc-Ar), 120.04 (s, Fmoc-Ar), 85.86 (s, C4'), 83.84 (s, C1'), 71.57 (s, C3'), 65.18 (s, CH_2 [Fmoc]), 46.74 (s, CH [Fmoc]), 41.11 (s, C5'), 39.93 (s, CH_2), 38.45 (s, C2'), 32.63 (s, CH_2), 25.64 (s, CH_2). HRMS (ESI) m/z calc. for $C_{36}H_{35}N_7O_6Na$ $[M+Na]^+$ 684.2541, found 684.2511.

5'-*N*-(4-Methoxytrityl-amino-1-oxobutyl)amino-*N*^{6}-benzoyl-2',5'-dideoxyadenosine (15)

Piperidine (0.600 mL, 6.07 mmol) to a solution of **14** (400 mg, 0.605 mmol) in anhydrous DMF (6 mL). The reaction mixture was stirred at rt for 30 min. After the solvents were removed *in vacuo*, the residue was partitioned between diethyl ether/H_2O (60 mL/60 mL). The aqueous phase was separated and concentrated under nitrogen flow. The residue was co-evaporated with anhydrous acetonitrile for three times to give a white solid that was kept under vacuo overnight. The solid was dissolved in anhydrous pyridine (10 mL) and stirred under argon atmosphere before 4-methoxytriphenylmethyl chloride (450 mg, 1.45 mmol) was added. The reaction mixture was stirred at rt for 7 h before EtOH (3 mL) was added to quench the reaction. After the solvents were removed under reduced pressure, the residue was dissolved in CH_2Cl_2 (80 mL) and the organic phase was washed with H_2O (3x25 mL). The organic phase was evaporated *in vacuo*, and the residue was

purified by flash column chromatography on silica gel eluting with a stepwise gradient of MeOH in CH_2Cl_2 (0-10%) to furnish the final product as a white foam (360 mg, 83%). *Rf* = 0.36 (10% MeOH in CH_2Cl_2). ^{1}H NMR (400 MHz, $CDCl_3$) δ ppm 9.15 (s, 1H, NH), 8.75 (s, 1H, H2), 8.05 (s, 1H, H8), 8.01 (d, *J* = 7.3 Hz, 2H, Bz-Ar), 7.80 – 7.73 (m, 1H, NH), 7.65 – 7.58 (m, 1H, Bz-Ar), 7.55 – 7.47 (m, 2H, Bz-Ar), 7.44 – 7.39 (m, 4H, MMTr-Ar), 7.34 – 7.30 (m, 2H, MMTr-Ar), 7.24 – 7.19 (m, 4H, MMTr-Ar), 7.15 – 7.09 (m, 2H, MMTr-Ar), 6.76 (d, *J* = 8.9 Hz, 2H, MMTr-Ar), 6.34 – 6.23 (m, 1H, H1'), 5.28 (s, 1H, 3'-OH), 4.46 – 4.40 (m, 1H, C3'), 4.22 – 4.17 (m, 1H, C4'), 4.05 – 3.95 (m, 1H, C5'), 3.73 (s, 3H, OCH_3), 3.41 – 3.26 (m, 1H, C5'), 2.93 – 2.80 (m, 1H, C2'), 2.47 – 2.35 (m, 2H, CH_2), 2.34 – 2.27 (m, 1H, C2'), 2.20 – 2.12 (m, 2H, CH_2), 1.89 – 1.80 (m, 2H, CH_2). ^{13}C NMR (101 MHz, $CDCl_3$) δ ppm 174.24 (s, C=O), 164.73 (s, C=O), 157.92 (s, MMTR-Ar), 152.30 (s, C2), 151.23 (s, C6), 150.23 (s, C4), 146.42 (s, MMTr-Ar), 142.76 (s, C8), 138.32 (s, MMTr-Ar), 133.61 (s, Bz-Ar), 133.05 (s, Bz-Ar), 129.88 (s, MMTr-Ar), 129.03 (s, MMTr-Ar), 128.59 (s, Bz-Ar), 128.04 (s, MMTr-Ar), 127.90 (s, MMTr-Ar), 126.30 (s, MMTr), 124.50 (s, C5), 113.25 (s, MMTr-Ar), 87.04 (s, C4'), 86.26 (s, C1'), 72.24 (s, MMTr), 70.47 (s, C3'), 55.32 (s. MMTr [OCH_3]), 43.38 (s, CH_2), 41.13 (s, C5'), 39.57 (s, C2'), 34.70 (s, CH_2), 26.79 (s, CH_2). HRMS (ESI) m/z calc. for $C_{41}H_{41}N_7O_5Na$ $[M+Na]^+$ 734.3061, found 734.3074; m/z calc. for $C_{41}H_{42}N_7O_5$ $[M+H]^+$ 712.3242, found 712.3264.

5'-*N*-(4-Methoxytrityl-amino-1-oxobutyl)amino-*N*^{6}-benzoyl-2',5'-dideoxyadenosine-3'-*O*-(2-cyanoethyl-*N*,*N*-diisopropylamino)phosphoramidite (16)

2-Cyanoethyl *N*,*N*-diisopropylchlorophosphoramidite (0.160 mL, 0.710 mmol) was added to a solution of **15** (340 mg, 0.478 mmol) and DIPEA (0.160 mL, 0.960 mmol) in anhydrous CH_2Cl_2 (10 mL). The reaction mixture was stirred at rt for 1 h under argon atmosphere. To this KCl solution (sat. aq., 6 mL, purged with argon) was added, and the organic phase separated, dried (Na_2SO_4) and evaporated. The residue was purified on flash column chromatography on silica gel eluting with MeOH in CH_2Cl_2 (0-4%) to furnish the final product as a white foam after precipitation in CH_2Cl_2/cold *N*-hexane (1:70, mL) system twice (330 mg, 97%). *Rf* = 0.76 (10% MeOH in CH_2Cl_2 ^{31}P NMR (162 MHz, $CDCl_3$) δ ppm 149.31 (s), 148.80 (s). HRMS (ESI) m/z calc. for $C_{50}H_{59}N_9O_6P$ $[M+H]^+$ 912.4320, found 912.4283.

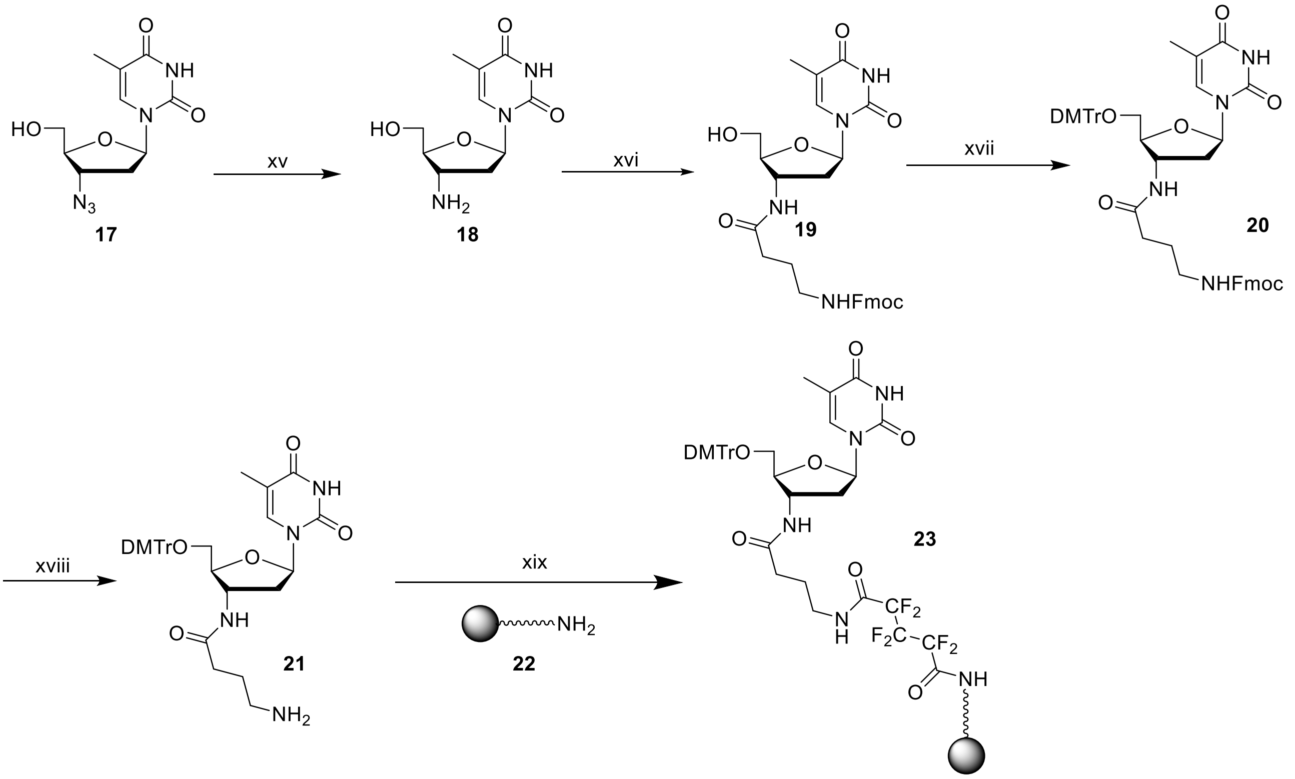

Supplementary Fig. 3. Synthesis of solid support **23**; xv) a) PPh_3, pyridine, rt, 40 min, b) 28% aq. ammonia solution, rt, 16 h, 95% for two steps; xvi) Fmoce-GABA-OH, HATU, DIPEA, DMF, rt, 2 h, 85%; xvii) 4,4'-dimethoxytrityl chloride, pyridine, rt, 4 h, 81%; xviii) piperidine, DMF, rt, 20 min, 95%; xix) a) hexafluoroglutaric anhydride, DIPEA,CH_2Cl_2, b) **21**, PPh_3, I_2, DMF, 14.7 µmol/g, 23% load efficiency.

3'-Amino-3'-deoxythymidine (18)

PPh_3 (1.37 g, 5.22 mmol) was added to a solution of 3'-azido-3'-deoxythymidine (930 mg, 3.48 mmol) in anhydrous pyridine (20 mL). The reaction mixture was stirred at rt for 40 min under N_2 atmosphere. Aqueous ammonia (20 mL, 28%) was added, and the resulting solution was further stirred at rt for 16 h. After the solution was concentrated under reduced pressure, the residue was partitioned between H_2O/CH_2Cl_2 (60mL/40mL). The aqueous phase was separated and washed with CH_2Cl_2 (2x40 mL) and concentrated under N_2-flow. The residue was co-evaporated from anhydrous acetonitrile three times to furnish the final product as a white solid (800 mg, 95%). ^{1}H NMR (400 MHz, DMSO-d_6) δ ppm 7.76 (d, J = 1.2 Hz, 1H, H6), 6.13 – 6.04 (m, 1H, H1'), 4.99 (s, 1H, NH), 3.68 – 3.47 (m, 3H, H4', H5', H5''), 3.44 – 3.36 (m, 1H, H3'), 2.10 – 2.05 (m, 1H, H1'), 2.02 – 1.94 (m, 1H, H2''), 1.76 (d, J = 1.1 Hz, 3H, CH_3). ^{13}C NMR (101 MHz, DMSO-d_6) δ ppm 163.76 (s, C4), 150.35 (s, C2), 136.29 (s, C6), 108.90 (s, C5), 87.60 (s, C4'), 83.45 (s, C1'), 60.75 (s, C5'), 50.76 (s, C3'), 40.65 (s, C2'), 12.20 (s, CH_3). Data is consistent with published NMR data.[5,6] HRMS (ESI) m/z calc. for $C_{10}H_{16}N_3O_4$ $[M+H]^+$ 242.1135, found 242.1143.

3’-*N*-(4-Fmoc-amino-1-oxobutyl)amino-3’-deoxythymidine (19)

DIPEA (2.32 mL, 13.3 mmol) and HATU (1.21 g, 3.19 mmol) were added into a solution of Fmoc-GABA-OH (1.04 g, 3.19 mmol) in anhydrous DMF (20 mL), and the resulting solution was stirred at rt for 10 minutes under argon atmosphere. To this **18** (700 mg, 2.90 mmol, dissolved in 5 mL anhydrous DMF) was added dropwise, and the reaction mixture was further stirred at rt for 2 h before CH_2Cl_2 (80 mL) and H_2O (70 mL) were added. The organic phase was separated, and the aqueous layer was extracted with CH_2Cl_2 (2x20 mL). All organic phases were combined, washed with brine (80 mL), and concentrated under reduced pressure. The residue was purified by flash column chromatography on silica gel eluting with MeOH in CH_2Cl_2 (0-10%) to furnish the final product as a white solid (1.27 g, 85%). *Rf*=0.27 (10% MeOH in CH_2Cl_2). ^{1}H NMR (400 MHz, DMSO-d_6) δ 11.29 (s, 1H, NH), 8.26 (d, *J* = 7.3 Hz, 1H, Fmoc-Ar), 7.89 (d, *J* = 7.5 Hz, 2H, Fmoc-Ar), 7.81 – 7.75 (m, 1H, H6), 7.71 – 7.61 (m, 2H, Fmoc-Ar), 7.46 – 7.38 (m, 2H, Fmoc-Ar), 7.37 – 7.24 (m, 3H, Fmoc-Ar, NH), 6.19 (t, *J* = 6.6 Hz, 1H, H1’), 5.07 (t, *J* = 5.2 Hz, 1H, 5’-OH), 4.38 – 4.26 (m, 3H, Fmoc, H3’), 4.26 – 4.16 (m, 1H, Fmoc), 3.81 – 3.71 (m, 1H, H4’), 3.69 – 3.50 (m, 2H, H5’, H5’’), 3.05 – 2.87 (m, 2H, CH_2), 2.27 – 2.16 (m, 1H, H_2’), 2.14 – 1.96 (m, 3H, CH_2, H_2’’), 1.78 (s, 3H,), 1.69 – 1.57 (m, 2H, CH_2). ^{13}C NMR (101 MHz, DMSO-d_6) δ 171.74 (s, C=O), 163.69 (s, C4), 156.07 (s, C=O), 150.39 (s, C2), 143.90 (s, Fmoc-Ar), 140.71 (s, Fmoc-Ar), 136.09 (s, C6), 127.55 (s, Fmoc-Ar), 127.01 (s, Fmoc-Ar), 125.09 (s, Fmoc-Ar), 120.06 (s, Fmoc-Ar), 109.35 (s, C5), 85.18 (s, C4’), 83.52 (s, c1’), 65.18 (s, C3’), 61.41 (s, C5’), 49.06 (s, CH_2 [Fmoc]), 46.76 (s, CH [Fmoc]), 39.85 (s, CH_2), 37.02 (s, C2’), 32.60 (s, CH_2), 25.55 (s, CH_2), 12.20 (s, CH_3). HRMS (ESI) m/z calc. for $C_{29}H_{32}N_4O_7Na[M+Na]^+$ 571.2163, found 571.2143.

5’-*O*-(4,4’-Dimethoxytrityl)-3’-*N*-(4-Fmoc-amino-1-oxobutyl)amino-3’-deoxythymidine (20)

4,4'-Dimethoxytrityl chloride (850 mg, 2.50 mmol) was added to a solution of **19** (1.00 g, 1.82 mmol) in anhydrous pyridine (20 mL). The reaction mixture was stirred at rt for 4 h under argon atmosphere. The solvents were removed under reduced pressure, and the residue was dissolved in CH_2Cl_2 (100 mL), washed with H_2O (2x40 mL) and evaporated. The residue was purified by flash column chromatography on silica gel eluting with a stepwise gradient of MeOH in CH_2Cl_2 (0-8%) to furnish the final product as a white foam (1.25 g, 81%). *Rf* = 0.61 (10% MeOH in CH_2Cl_2). ^{1}H NMR (400 MHz, $CDCl_3$) δ ppm 9.79 (s, 1H, NH), 8.66 – 8.57 (m, 1H, NH), 7.75 – 7.69 (m, 2H, Fmoc-Ar), 7.62 (s, 1H, H6), 7.57 – 7.51 (m, 2H, Fmoc-Ar), 7.50 – 7.43 (m, 1H, Fmoc-Ar), 7.42 – 7.32 (m, 4H, Fmoc-Ar, DMTr-Ar), 7.31 – 7.26 (m, 4H, DMTr-Ar), 7.25 – 7.18 (m, 4H, DMTr-Ar), 6.79 (d, *J* = 8.9 Hz, 4H, DMTr-Ar), 6.43 – 6.28 (m, 1H, H1’), 5.31 – 5.18 (m, 1H, NH), 4.78 – 4.61 (m, 1H, H3’), 4.38 (d, *J* = 6.8 Hz, 2H, CH_2 [Fmoc]), 4.14 (t, *J* = 6.7 Hz, 1H, CH [Fmoc]), 4.01 (s, 1H, H4’), 3.74 (d, *J* = 0.9 Hz, 6H, OCH_3),

3.50 – 3.34 (m, 2H, H5',H5''), 3.18 – 3.00 (m, 2H, CH_2), 2.52 – 2.22 (m, 2H, H2', H2''), 2.20 – 2.03 (m, 2H, CH_2), 1.91 – 1.69 (m, 2H, CH_2), 1.36 (s, 3H, CH_3). ^{13}C NMR (101 MHz, $CDCl_3$) δ ppm 173.00 (s, C=O), 164.10 (s, C4), 158.82 (s, DMT-Ar), 157.17 (s, C=O), 150.96 (s, C2), 149.94 (s, Ar), 144.45 (s, Ar), 144.02 (s, Ar), 143.98 (s, Ar), 141.44 (s, Ar), 136.09 (s, C6), 135.76 (s, Ar), 135.53 (s, Ar), 135.44 (s, Ar), 130.34 (s, Ar), 130.31 (s, Ar), 128.38 (s, Ar), 128.08 (s, Ar), 127.80 (s, Ar), 127.24 (s, Ar), 127.15 (s, Ar), 125.12 (s, Ar), 123.86 (s, Ar), 120.08 (s, Ar), 113.38 (s, Ar), 111.59 (s, C5), 87.09 (s, DMT), 84.94 (s, C4'), 84.61 (s, C1'), 66.73 (s, CH_2 [Fmoc]), 63.88 (s, C5'), 55.35 (s, OCH_3), 50.70 (s, C3'), 47.40 (s, CH [Fmoc]), 40.27 (s, CH_2), 38.23 (s, C2'), 33.31 (s, CH_2), 26.21 (s, CH_2), 11.82 (s, CH_3). HRMS (ESI) m/z calc. for $C_{50}H_{50}N_4O_9Na[M+Na]^+$ 873.3470, found 873.3437.

5'-*O*-(4,4'-Dimethoxytrityl)-3'-*N*-(4-amino-1-oxobutyl)amino-3'-deoxythymidine (21)

Piperidine (1 mL, 10.1 mmol) was added to a solution of **20** (1.00 g, 1.17 mmol) in anhydrous DMF (9 mL), and the resulting reaction mixture was stirred at rt for 20 min under argon atmosphere. Afterwards, solvents were removed under reduced pressure and the residue was redissolved in CH_2Cl_2 (3 mL) and precipitated out using cold diethyl ether (150 mL). The white precipitated was purified by flash column chromatography on silica gel, eluting with MeOH in CH_2Cl_2 (5-15%) before a solvent system of MeOH in EtOAc (40%) was used to furnish the final product as a white foam (708 mg, 95%). ^{1}H NMR (400 MHz, $CDCl_3$) δ ppm 8.30 (s, 1H, NH), 7.62 (s, 1H, H6), 7.46 – 7.35 (m, 2H, DMTr-Ar), 7.32 – 7.26 (m, 5H, DMTr-Ar), 7.26 – 7.15 (m, 2H, DMT-Ar), 6.81 (d, *J* = 8.7 Hz, 4H, DMTr-Ar), 6.56 – 6.40 (m, 3H, H1', R-NH_2), 4.75 (s, 1H, H3'), 4.12 – 3.96 (m, 1H, H4'), 3.76 (s, 6H, OCH_3), 3.50 – 3.34 (m, 2H, H5'), 2.88 – 2.74 (m, 2H, CH_2), 2.47 – 2.27 (m, 4H, CH_2, H2', H2''), 1.92 – 1.81 (m, 2H, CH_2), 1.28 (s, 3H, CH_3). ^{13}C NMR (101 MHz, $CDCl_3$) δ ppm 172.74 (s, C=O), 164.60 (s, C4), 158.73 (s, DMTr), 151.52 (s, C2), 144.42 (s, DMTr-Ar), 135.55 (s, C6), 135.49 (s, DMTr-Ar), 135.38 (s, DMTr-Ar), 130.21 (s, DMTr-Ar), 130.16 (s, DMTr-Ar), 128.27 (s, DMT-Ar), 127.97 (s, DMTr-Ar), 127.12 (s, DMTr-Ar), 113.28 (s, DMTr-Ar), 111.95 (s, C5), 87.01 (s, DMTr), 85.37 (s, C4'), 84.44 (s, C1'), 64.41 (s, C5'), 55.25 (s, OCH_3), 50.98 (s, C3'), 39.65 (s, CH_2), 38.14 (s, C2'), 33.11 (s, CH_2), 26.63 (s, CH_2), 11.59 (s, CH_3). HRMS (ESI) m/z calc. for $C_{35}H_{41}N_4O_7$ $[M+H]^+$ 629.2969, found 629.2969.

5'-*O*-(4,4'-Dimethoxytrityl)-3'-*N*-(4-amino-1-oxobutyl)amino-3'-deoxythymidine-LCAA-CPG solid support (23)

LCAA-CPG (**22**, 700 mg, loading 64 μmol/g) was co-evaporated with anhydrous toluene for two times and then dried on the vacuum line for 14 h. To this anhydrous CH_2Cl_2 (6 mL) and DIPEA (203 μL, 0.650 mmol), and hexafluoroglutaric anhydride (74 μL, 0.550 mmol) were subsequently added as

the given order. The resulting slurry was gently stirred at rt for 16 h. The solid support was then treated with a sequential wash process as following: After the solution was filtered out, the remaining solid support was firstly washed on the filter with CH_2Cl_2 (10 mL) and then transferred to a falcon tube as a white solid. To this DMF (10 mL, 1% Et_3N) was added, and the mixture was vortexed at rt for 15 min (1000 rpm, Thermomixer Compact). The falcon tube was centrifuged for 4 min until all the CPG was sedimented in the bottom of the tube (Heraeus Multifuge S-R, 1100 min^{-1}xg), to have the supernatant removed as much as possible. This DMF rinse procedure was carried out five times in total (5x10 mL, DMF with 1% Et_3N). The slurry residue was filtered to remove the remaining DMF. The CPG solid support was then washed with CH_2Cl_2 (3x10 mL) on the filter and transferred to a new falcon tube. To this MeOH (10 mL) was added. The mixture was vortexed at rt for 15 min (1000 rpm, Thermomixer Compact). The falcon tube was centrifuged for 4 min (Heraeus Multifuge S-R, 1100 min^{-1}xg), and the supernatant was removed as much as possible. This MeOH rinse procedure was carried out five times in total (5x10 mL, MeOH). To the remaining slurry CH_2Cl_2 (10 mL) was added. The mixture was vortexed at rt for 15 min (1000 rpm, Thermomixer Compact). The falcon tube was centrifuged for 4 min (Heraeus Multifuge S-R, 1100 min^{-1}xg), and the supernatant was removed as much as possible. This CH_2Cl_2 rinse procedure was repeated five times in total (5x10 mL, CH_2Cl_2). The remaining solvent(s) was removed by filtration to complete the sequential wash and the residue CPG solid support was co-evaporated with toluene (anhydrous) before it was dried on the vacuum line for 4 h. To this **21** (100 mg, 160 μmol) was added. The resulting mixture was co-evaporated with anhydrous toluene for two times and then dried on the vacuum line for 16 h. The white solid mixture was suspended in anhydrous DMF (5 mL) before DIPEA (160 μL, 0.580 mmol) and PPh_3 (650 mg, 244 μmol) was firstly added. To this suspension, I_2 (65.0 mg, 244 μmol, dissolved in 1 mL anhydrous DMF) was added dropwise. The resulting reddish slurry was gently stirred for 16 hours before the CPG was filtered and washed with DMF (2x10 mL, 1% Et_3N) and CH_2Cl_2 (2x10 mL) on filter. The white solid was treated with the sequential wash process using DMF containing 1% Et_3N, MeOH and CH_2Cl_2 (*vide supra*) and dried on the vacuum line for 24 h. The loading of the modified nucleoside on the LCAA-CPG was determined as 14.7 μmol/g (23% loading efficiency) from the ratio of two crude ON products used for this study (**ON1** and **ON4**, with an extra 4-amino-1-butanoyl linker on the 3'-amino group for the latter). **ON1** was synthesized from a commercially available 3'-Amino-dT-CPG (28 μmol/g, Glen Research, 20-2981-01).

S4 Oligonucleotide synthesis, purification, and analysis

Oligonucleotide synthesis was carried out on an ÄKTA oligopilot plus 10 system in 1.0 µmol scale (polystyrene support) using the phosphoramidite approach and following manufacturer's standard protocols. The coupling time for standard DNA monomers was 720 s and stepwise coupling efficiencies in all cases were >98.0%, which were determined by the absorbance of trityl cation at 500 nm.

For **ON1**, **ON2**, **ON3**, **ON4**, **ON5 and ON6**, the primary amine function was introduced into oligonucleotides either on the 5'-end or the 3'-end using the corresponding nucleoside phosphoramidite monomers or special solid CPG supports to serve as a reacting handle for BCN functionalization in post-ON synthesis (*vide infra*). Specifically, for **ON1**, **ON2**, **ON4** and **ON5**, phosphoramidite monomer **4**, **13**, **7** and **16** were respectively incorporated into the 5'-end of the oligonucleotides *via* manual-coupling using 5-[3,5-bis(trifluoromethyl)phenyl]-*H*-tetrazole (0.25 M, in anhydrous acetonitrile) as activator and extended coupling time (15 min). For **ON7**, **ON8** , **ON10**, **ON11**, **ON13**, **ON14**, **ON16** and **ON17**, 5'-amino-modifier 5 (Glen research) alone, 5'-amino-modifier 5 and Spacer Phosphoramidite C3, 5'-amino-modifier 5 and Spacer Phosphoramidite 9 or 5'-amino-modifier 5 and Spacer Phosphoramidite 18 were used to furnish the wanted linker length and the primary amine function on the 5'-end. Whereas, the primary amine function on the 3'-end (**ON3**, **ON6** and **ON9**) was realized by starting the oligonucleotide synthesis from three special solid supports, 3'-amino-dT-CPG (Glen research), **23** and 3'-PT-amino-modifier C6 PS (Glen research). Beside 3'-PT-amino-modifier C6 PS, three phosphoramidite monomers including Spacer Phosphoramidite 3, Spacer Phosphoramidite 9 and Spacer Phosphoramidite 18 were used for the synthesis of **ON12**, **ON15** and **ON18**, respectively. For **ON9**, a protected phosphate group was furnished on 5'-end using chemical phosphorylation Reagent II or solid chemical phosphorylation reagent II (the deprotection was carried out following the standard protocols from Glen Research). Upon completion the synthesized oligonucleotides attached to this special solid support were treated with 20% diethylamine in acetonitrile (5 mL) for 10 min then washed with acetonitrile extensively. This procedure removes cyanoethyl groups from the phosphotriesters and scavenges the resultant acrylonitrile, preventing cyanoethyl adducts being formed at the primary amine on the 3'-end. Standard deprotection and purification were then carried out for all eighteen synthesized oligonucleotides. Cleavage from solid support and removal of nucleobase protecting groups were performed using 28% aqueous ammonia, 16 h at 55 °C. The resulting oligonucleotides were purified

by MMTr-ON or DMTr-ON RP-HPLC using the Waters System 600 equipped with a Waters XBridge BEH C18-column (5 µm/ 135 Å, 100 mm × 19 mm). Elution was performed starting with an isocratic hold of A-buffer for 5 min followed by a linear gradient to 70% B-buffer over 16.5 min at a flow rate of 5.0 mL/min (A-buffer: 0.05 M triethylammonium acetate in Milli-Q water, pH 7.4; B-buffer: 25% A-buffer, 75% acetonitrile). After all the solvents were removed under nitrogen flow, oligonucleotides were detritylated using an 80% aqueous solution of acetic acid for 20 min, then desalted with an aqueous solution of sodium acetate (3 M, 15 µL) and sodium perchlorate (5 M, 15 µL) followed by cold ethanol (1 mL). The resulting suspension was stored at -20 °C for 1 h. After centrifugation (16100 × g, 5 min, 4 °C), the supernatant was removed and the pellet further washed with cold ethanol (2 × 1 mL), dried for 30 min under nitrogen flow, and dissolved in Milli Q water (1.0 mL). For some of the long oligonucleotides (purity < 90%), further purification was carried out to remove the shoulder peaks. Those oligonucleotides were purified by anion-exchange HPLC (IE-HPLC) using the DIONEX Ultimate 3000 system equipped with a DNAPac PA100 semipreparative column (13 µm, 250 mm × 9 mm) heated to 60 °C. Elution was performed with an isocratic hold of buffer B (10%), starting from 2 min hold on 2% Buffer A in Milli-Q water (solvent A), followed by a linear gradient to 25% buffer A in 20 min at a flow rate of 2.0 mL/min (buffer A: 1.0 M sodium perchlorate; buffer B: 0.25 M Tris-Cl, pH 8.0, solvent A: Milli-Q water). After IE-HPLC purification, the proper fractions were combined, all the solvents were removed under nitrogen flow, oligonucleotides were dissolved in Milli-Q water (100 µL), and desalted with an aqueous solution of sodium perchlorate (5 M, 15 µL) followed by cold ethanol (1.5 mL). The resulting suspension was stored at -20 °C for 1 h. After centrifugation (16100 × g, 5 min, 4 °C), the supernatant was removed and the pellet further washed with cold ethanol (2 × 1 mL), dried for 30 min under nitrogen flow, and dissolved in Milli Q water (1.0 mL).

Mass spectra of oligonucleotides were recorded either on a Waters Xevo G2-XS G TOF with Waters Aquity UPLC system in ES^- mode or on a Bruker Daltonics Microflex LT MAIDI-TOF MS instrument in positive mode (representative MS in Supplementary Fig. 4–9). Analytical IE-HPLC traces were recorded on a Merck-Hitachi Lachrom system equipped with a DNAPac PA100 analytical column (13 µm, 250 mm × 4 mm) heated to 60 °C. Elution was performed with an isocratic hold of buffer B (10 %), starting from 2 min hold using 2% buffer A in Milli-Q water, followed by a linear gradient to 30% buffer A in 23 min at a flow rate of 1.1 mL/min (buffer A: 1.0 M sodium perchlorate; buffer B: 0.25 M Tris-Cl, pH 8.0) (representative IE-HPLC traces in Supplementary Fig. 4–9). Concentrations of purified oligonucleotides were determined by UV absorption measurements at 260 nm.

S5 BCN-functionalization in post-ON synthesis

The NH_2-functionalized ONs (**ON1-18**, dissolved in Milli-Q H_2O, Supplementary Fig. 10), carbonate buffer (pH 8.75, 0.5 M) and a solution of click-easy® BCN *N*-hydroxysuccinimide ester I (30 eq., Berry&Associates) in acetonitrile were mixed (1:1:1, v/v/v) in a round bottle flask. The resulting solution was stirred at room temperature for 2 h. The mixture was evaporated *in vacuo* to remove most of the solvents and Milli-Q H_2O was then added. The crude was desalted using Illustra NAP™-10 column (GE Healthcare) to remove the inorganic salts and excess reagents.

RP-HPLC analysis was performed on the desalted crude **ON-BCN**s (representative RP-HPLC traces in Supplementary Fig. 11-16). Analytical RP-HPLC traces were recorded on a Merk Hitachi 7000 system equipped with a Waters XBridge OBD C18-column (2.5 µm/130 Å, 10×50 mm) or a Phenomenex Kinetex C8-column (5 µm/100 Å, 4.6×150 mm, core-shell technology) heated to 50 °C. Elution was performed at 60 °C with an isocratic hold of A-buffer for 2 min followed by a linear gradient to 70% B-Buffer over 28 min at a flow rate of 1 mL/min for C8-column or 1.3 mL/min for C18-column.

The crude **ON-BCN**s were purified by RP-HPLC using a Waters System 600 HPLC equipment equipped with a Waters XBridge BEH C18-column (5 µm/135 Å, 100 mm × 19 mm) or a Phenomenex Kinetex C8-column (5 µm/100 Å, 21.2×100 mm, core-shell technology). Elution was performed starting with an isocratic hold of A-buffer for 2 min followed by a linear gradient to 70% B-buffer over 16.5 min at a flow rate of 5.0 mL/min (A-buffer: 0.05 M triethylammonium acetate in Milli-Q water, pH 7.4; B-buffer: 25% A-buffer, 75% acetonitrile). After removal of the solvents under a flow of nitrogen, the oligonucleotide products were dissolved in Milli-Q water (100 µL) and then desalted with an aqueous solution of sodium perchlorate (5 M, 15 µL) followed by cold ethanol (1.5 mL). The resulting suspension was stored at -20 °C for 1 h. After centrifugation (13200 × g, 5 min, 4 °C), the supernatant was removed and the pellet further washed with cold ethanol (2 × 1 mL), dried for 30 min under nitrogen flow, and dissolved in Milli Q water (1.0 mL).

Linker length	Starting material	Amount	Product	Amount	Overall yield
16 bonds	**ON1**	540 nmol	**ON1-BCN**	368 nmol	68%
	**ON2**	363 nmol	**ON2-BCN**	269 nmol	74%
	**ON3**	375 nmol	**ON3-BCN**	217 nmol	58%
21 bonds	**ON4**	270 nmol	**ON4-BCN**	195 nmol	72%
	**ON5**	141 nmol	**ON5-BCN**	103 nmol	73%
	**ON6**	118 nmol	**ON6-BCN**	82 nmol	69%
24-25 bonds	**ON7**	--	**ON7-BCN***	--	--
	**ON8**	303 nmol	**ON8-BCN**	212 nmol	70%
	**ON9**	193 nmol	**ON9-BCN**	96 nmol	50%
30-31 bonds	**ON10**	180 nmol	**ON10-BCN**	119 nmol	66%
	**ON11**	101 nmol	**ON11-BCN**	67 nmol	66%
	**ON12**	107 nmol	**ON12-BCN**	63 nmol	59%
35-36 bonds	**ON13**	360 nmol	**ON13-BCN**	209 nmol	58%
	**ON14**	323 nmol	**ON14-BCN**	228 nmol	71%
	**ON15**	300 nmol	**ON15-BCN**	168 nmol	56%
44-45 bonds	**ON16**	270 nmol	**ON16-BCN**	205 nmol	76%
	**ON17**	161 nmol	**ON17-BCN**	110 nmol	68%
	**ON18**	171 nmol	**ON18-BCN**	110 nmol	64%

***ON7-BCN** is a known compound and synthesized following the reported method.[7]

IE-HPLC analysis and ESI-MS or MALDI-TOF MS were performed on all 18 **ON-BCN** products as described in **S4** (*vide supra*, and representative MS and IE-HPLC traces in Supplementary Fig. 17–22). Concentrations of **ON-BCN**s are determined by UV absorption measurements at 260 nm, upon which the overall reaction yields were calculated.

S6. POC synthesis, purification and analysis

ON-BCNs (39-261 nmol, 0.98 mL, dissolved in Milli-Q water, Figure 2) was added to a solution of azidopeptide (1.25 eq, 49-326 nmol) in DMSO (0.98 mL). The resulting solution was transferred to a Biotage microwave reaction vial (2 mL) and sealed under an atmosphere of nitrogen. The reaction was carried out on a Biotage Initiator microwave synthesizer at 60 °C for 3 h whereupon all solvents were removed *in vacuo*. The residue was redissolved in Milli-Q water (2 mL).

RP-HPLC analysis was performed on the crude **POC**s (representative RP-HPLC traces in Figure S20, Figure S21, Figure S22, Figure S23, Figure S24, Figure S25, Figure S26, Figure S27, Figure S28, Figure S29 and Figure S30). Analytical RP-HPLC traces were recorded on a Merk Hitachi 7000 system equipped with a Waters XBridge OBD C18-column (2.5 µm/130 Å, 10×50 mm) or a Phenomenex

Kinetex C8-column (5 µm/100 Å, 4.6×150 mm, core-shell technology) heated to 50 °C. Elution was performed at 50 °C with an isocratic hold of A-buffer for 2 min followed by a linear gradient to 70% B-Buffer over 28 min at a flow rate of 1 mL/min for C8-column or 1.3 mL/min for C18-column.

The 33 crude POCs were purified by RP-HPLC using a Waters System 600 HPLC equipment equipped with a Waters XBridge BEH C18-column (5 µm/135 Å, 100 mm × 19 mm). Elution was performed starting with an isocratic hold of A-buffer for 2 min followed by a linear gradient to 70% B-buffer over 16.5 min at a flow rate of 5.0 mL/min (A-buffer: 0.05 M triethylammonium acetate in Milli-Q water, pH 7.4; B-buffer: 25% A-buffer, 75% acetonitrile). After removal of the solvents under a flow of nitrogen, the resulting residues were dissolved in Milli-Q water (150 µL) and desalted *via* precipitation by first adding two aqueous solution of sodium acetate (3 M, 15 µL) and sodium perchlorate (5 M, 15 µL) followed by addition of cold ethanol (1.5 mL, 99% w/w; -20 °C). The resulting suspensions were stored at -20 °C for 1 h, and after centrifugation (16100 × g, 5 min, 4 °C) the supernatants were removed and the pellet further washed with cold ethanol (2 × 1 mL; -20 °C), and dried for 30 min under a flow of nitrogen. The precipitation and washing process was repeated one more time. The pellet was then dissolved in Milli-Q water (1.0 mL) to give 33 desired POC products (*vide infra*).

Linker length	Starting material	Amount	Azidopeptide	Product	Amount	Overall yield
16 bonds	**ON1-BCN**	162 nmol	L-Azidopeptide	**L,D-POC1**	119 nmol	73%
		162 nmol	D-Azidopeptide	**D,D-POC1**	100 nmol	62%
	**ON2-BCN**	125 nmol	L-Azidopeptide	**L,D-POC2**	74 nmol	59%
		125 nmol	D-Azidopeptide	**D,D-POC2**	70 nmol	56%
	**ON3-BCN**	101 nmol	L-Azidopeptide	**L,D-POC3**	71 nmol	70%
		101 nmol	D-Azidopeptide	**D,D-POC3**	65 nmol	64%
21 bonds	**ON4-BCN**	81 nmol	L-Azidopeptide	**L,D-POC4**	50 nmol	62%
		81 nmol	D-Azidopeptide	**D,D-POC4**	47 nmol	58%
	**ON5-BCN**	49 nmol	L-Azidopeptide	**L,D-POC5**	31 nmol	63%
		49 nmol	D-Azidopeptide	**D,D-POC5**	30 nmol	61%
	**ON6-BCN**	39 nmol	L-Azidopeptide	**L,D-POC6**	26 nmol	67%
		39 nmol	D-Azidopeptide	**D,D-POC6**	24 nmol	62%
24-25 bonds	**ON7-BCN**	--	L-Azidopeptide	**L,D-POC7**	30 nmol*	--
		--	D-Azidopeptide	**D,D-POC7**	20 nmol*	--
	**ON8-BCN**	242 nmol	L-Azidopeptide	**L,D-POC8**	122 nmol	50%
		261 nmol	D-Azidopeptide	**D,D-POC8**	140 nmol	54%
	**ON9-BCN**	111 nmol	L-Azidopeptide	**L,D-POC9**	71 nmol	64%
		164 nmol	D-Azidopeptide	**D,D-POC9**	113 nmol	69%
30-31 bonds	**ON10-BCN**	171 nmol	L-Azidopeptide	**L,D-POC10**	127 nmol	74%
		100 nmol	D-Azidopeptide	**D,D-POC10**	72 nmol	72%
	**ON11-BCN**	101 nmol	L-Azidopeptide	**L,D-POC11**	72 nmol	71%
		60 nmol	D-Azidopeptide	**D,D-POC11**	38 nmol	63%
	**ON12-BCN**	97 nmol	L-Azidopeptide	**L,D-POC12**	74 nmol	76%
		60 nmol	D-Azidopeptide	**D,D-POC12**	47 nmol	78%
35-36 bonds	**ON13-BCN**	101 nmol	L-Azidopeptide	**L,D-POC13**	68 nmol	67%
		101 nmol	D-Azidopeptide	**D,D-POC13**	68 nmol	67%
	**ON14-BCN**	90 nmol	L-Azidopeptide	**L,D-POC14**	58 nmol	64%
		90 nmol	D-Azidopeptide	**D,D-POC14**	59 nmol	66%
	**ON15-BCN**	82 nmol	L-Azidopeptide	**L,D-POC15**	53 nmol	65%
		82 nmol	D-Azidopeptide	**D,D-POC15**	53 nmol	65%
44-45 bonds	**ON16-BCN**	180 nmol	L-Azidopeptide	**L,D-POC16**	143 nmol	79%
	**ON17-BCN**	99 nmol	L-Azidopeptide	**L,D-POC17**	64 nmol	65%
	**ON18-BCN**	104 nmol	L-Azidopeptide	**L,D-POC18**	78 nmol	75%

*__L,D-POC7__ and **D,D-POC7** are known compounds and synthesized following the reported method.[7]

IE-HPLC analysis and ESI-MS or MALDI-TOF MS were performed on all 33 POC products as described in **S4** (*vide supra*, and representative MS and IE-HPLC traces in Supplementary Fig. 34–44). Analytical IE-HPLC traces were recorded on a Merck-Hitachi Lachrom system equipped with a DNAPac PA100 analytical column (13 µm, 250 mm × 4 mm) heated to 60 °C. Elution was performed with an isocratic hold of buffer B (10%), starting from 2 min hold on 2% Buffer A in Milli-Q water, followed by a linear gradient to 30% buffer A in 23 min at a flow rate of 1.1 mL/min (buffer A: 1.0 M sodium perchlorate; buffer B: 0.25 M Tris-Cl, pH 8.0) (representative IE-HPLC traces in Supplementary Fig. 34–44). Concentrations of POCs were determined by UV absorption measurements at 260 nm, upon which the overall reaction yields were calculated.

S7 CD Spectroscopy

Five extra ONs (DNA sequence) were synthesized for CD measurement:

		Triplex
**ON22**	5'-CAAATACTGTCCTTCTAGTGTAGC	
**ON19**		3'-TTTTCCCTCTCTCT
**ON20**	3'-GTTTATGACAGGAAGATCACATCGTT	ATGCCGCTAAAAGGGAGAGAGA
**ON21**	5'-GGCCGACGCGCTGGGCTACGTCTTGCTGGCTT	TACGGCGATTTTCCCTCTCTCT
**ON23**	3'-CCGGCTGCGCGACCCGATGCAGAACGACCG	

Far UV CD data were recorded on a JASCO J-815 calibrated with ammonium d-10-camphorsulfonate using a cell with a path-length of 0.2 cm from Hellma Analytics. All samples were dissolved in 10 mM MES buffer (300 µL, pH 5.5, 100 mM NaCl), with a concentration of 3.0 µM for each of the five ON/POCs strands. Thus, the total peptide concentration for POC triplex samples were 9 µM. All ON/POC triplex samples were prepared as following: The triplex-forming strand (**ON19**, **POC1**, **POC4**, **POC7**, **POC10**, **POC13** and **POC16**), the corresponding DNA/POC duplex and two ONs (**ON22** and **ON23**, complementary to the two remaining ssDNA regions in as-formed ON/POC triplexes) were mixed in a 2 mL Eppendorf tube followed by addition of 2× MES buffer (20 mM MES, 200 mM NaCl, pH 5.5, 150 µL). To this Milli-Q water was added to a total volume of 300 µL. Thus, all triplex samples were dissolved in 1 × MES buffer condition (10 mM MES, 100 mM NaCl, pH 5.5). The samples were denatured by heating to 90 ˚C in a water-bath followed by slow cooling to room temperature and storage overnight in a fridge before they were transferred into cuvettes.

CD spectroscopy scans were recorded between 400-200 nm, with a scanning speed of 50 nm/min, and 5 cycles. The buffer was measured separately and subtracted from the CD scans. The recorded spectra were then converted to Mean Residue Ellipticity (MRE, Supplementary Fig. 51).

S8 SAXS experiments and data treatment

Table S1: SAXS data collection details

Instrument	BM29 BIOSAXS beamline (ESRF)
Date	18.02.2021
Detector	Pilatus 2M
Wavelength (Å)	0.998
Beam size (μm^2)	500 x 100
Detector distance (m)	2.83
q-measurement range (nm^{-1})	0.039-5.183
Absolute scaling method	Comparison with scattering from pure H_2O
Normalization	To transmitted intensity by beam-stop counter
Monitoring for radiation damage	Frame-by-frame comparison
Exposure time (s)	10 x 1
Sample configuration	Quartz glass capillary
Sample temperature (ºC)	20
c) Software employed for SAXS data reduction, analysis and interpretation	
SAS data reduction	*PRIMUSqt*[16] from ATSAS 2.8.32
Basic analyses: Guinier, $p(r)$, V_P	*PRIMUSqt*[16] from ATSAS 2.8.32
Molecular graphics	*PyMOL* (version 1.8.2.3, Schrödinger, LLC)
Figures	*Spyder (anaconda 3)*

The structure of **D,D-POC(7+8+9)***in solution was studied with SAXS at concentrations of 10 µM and 50 µM. SAXS data were collected at the BM29 BIOSAXS beamline at ESRF (Grenoble, FR). 40 µl of 10 µM and 50 µM **D,D-POC(7+8+9)*** were measured, while the corresponding buffer control was measured twice, before and after the two POC triplex samples. 10 frames for each sample and buffer measurements were collected and were further checked for radiation damage. All radiation damage free frames were averaged and **D,D-POC(7+8+9)*** scattering curves were obtained by subtracting the pure buffer signal from the sample signal. The obtained scattering curves were normalized by concentration to the 50 µM sample. All data analyses, including determination of radius of gyration (R_g), maximum dimension (D_{max}), and I_0, were performed using the graphical data analysis program *PRIMUSQT*.[8] For more details on the experimental procedure see Table S1.

S9 SAXS analysis

Using the same pH condition and ionic strength, **D,D-POC(7+8+9)*** was prepared in 10 µM and 50 µM for SAXS measurement and the results were compared to its diastereoisomer **L,D-POC(7+8+9)*** characterized in our prior work [denoted as **L,D-(POC1+POC2+POC3)** in the paper].[7]

Key SAXS parameters are summarized in Table S2. Guinier analysis was performed on the Guinier region (low-*q* part of the intensity curve). In this region, intermolecular interactions greatly affect calculated molecular weight (MW). The Guinier plots (Supplementary Fig. 54A, inset) showed no significant changes in intermolecular interactions when comparing 10 µM and 50 µM. This supports that **D,D-POC(7+8+9)*** is a well-defined heterotrimeric structure that does not form aggregates. The Kratky plots (Supplementary Fig. 54B) indicated folded, but flexible complexes. We focused this analysis on the low concentration (10 µM) preparation.

Pair-distance distribution function analysis (Supplementary Fig. 54C) was performed on the whole intensity curve, excluding the initial low-*q* part in case of attractive/repulsive interactions. We note that D_{max} is comparable to a similar system at 7.4 µM studied previously.[9] D_{max} is also in reasonable agreement with the maximum heavy-atom distance of 115 Å measured in the MD structure (see below and inset in Supplementary Fig. 55).

MD simulations were then carried out to produce an ensemble of conformations and their predicted SAXS curves were compared with the experimental data. The *ab initio* models (Supplementary Fig. 54D) are elongated structures with a bend that is more pronounced at 50 µM. We ascribe this to flexibility in the linker region, since our previous characterization[7] of the diastereomer **L,D-POC(7+8+9)*** demonstrated a similar bend in the linker region connecting the oligonucleotide and peptide domains.

c (µM)	*c* (g/L)	Corrected *c* (g/L)	Guinier	*p*(*r*)			MW (kDa)		
			R_g	R_g	D_{max}	Porod volume (nm^3)	Guinier	*p*(*r*)	Porod volume (nm^3)
10	0.25	0.31	3.27	3.56	12.7	38.9	31	31	23
50	1.27	1.27	3.41	3.60	12.7	38.0	33	33	22

Table S2: Calculated SAXS parameters. Data analysis was performed assuming that **D,D-POC(7+8+9)***is all protein, therefore the calculated MW is not exact. Furthermore, the Porod volume was used to calculate the MW. This approach assumes a globular structure. For elongated structures such as in the present case, the calculated MW is thus approximative.The MW of 26.4 kDa was used to convert concentration to g/L. R_g and D_{max} are not affected by concentration. Distance measures are given in nm.

S10 Molecular modelling and fitting to SAXS data

A molecular model of **D,D-POC(7+8+9)*** was built with a similar approach as used previously for related POC structures[7,9]. The DNA triplex was identical with the idealized structure built with BIOVIA Discovery studio visualizer[10] in our previous study[7] as described in the Supporting Information for that article. The three linkers in their initial extended conformations were also inherited from that study[7], and were attached identically to the DNA triplex termini. However, the peptide domain differed: In order to obtain the desired all-D peptide coiled coil domain with right-

handed super-helicity, we inverted the cartesian coordinates of the previously used segment of the coil-VaLd crystal structure.[11] The resulting all-D coiled-coil domain was then attached to the free linker ends projecting axially from the DNA triplex, yielding an initial **D,D-POC(7+8+9)*** model. In order to sample the conformational preferences of this model, it was subjected to MD simulations in Desmond[12]. In preparation for this, the molecular model was solvated in an orthorhombic box of TIP3P[13] water leaving a buffer region of at least 10 Å on all sides of the model. Na^+ counterions were added to give an overall charge neutral system and an additional ionic background of 0.15 M NaCl[14] was used. The OPLS3[15] force field was assigned to the macromolecule. As done previously[7], we addressed the lack of reliable OPLS3 parameters for the DNA triplex by assigning positional harmonic restraints on the nitrogen and oxygen atoms in all oligonucleotide bases. This would allow sampling the conformations of the peptide and linkers relative to the DNA triplex which remained constrained to its idealized initial structure during the simulation. The solvated system was simulated for 400 ns in the NPT ensemble with standard pressure and temperature settings in Desmond[16].

The MD trajectory was processed in VMD[17] which was used to remove waters and ions and to save several thousand conformations of the **D,D-POC(7+8+9)*** model. The theoretical SAXS curve for each conformation was calculated with FoXS[18,19] followed by fitting to the experimental SAXS profile and evaluation of goodness-of-fit (FoXS $\chi 2$).

S11 Non-denaturing polyacrylamide gel electrophoresis

The samples were prepared in MES buffer (100 mM, pH 5.5), with a concentration of 30 µM for **TFO alone**, **D,D-POC7** and **L,D-POC7**. Each sample (2.5 µL) was mixed with equivalent volume of Ficoll 400 solution (2.5 µL, 15%, w/w in 100 mM MES buffer, pH 5.5) and then loaded together with O'GeneRuler Ultra Low Range DNA Ladder onto a 13% non-denaturing polyacrylamide gel for electrophoresis at a low current (10 mA) in the cold room (4 °C) and visualisation by UV excitation at 302 nm on a Syngene Geni Imager after SYBR Gold staining.

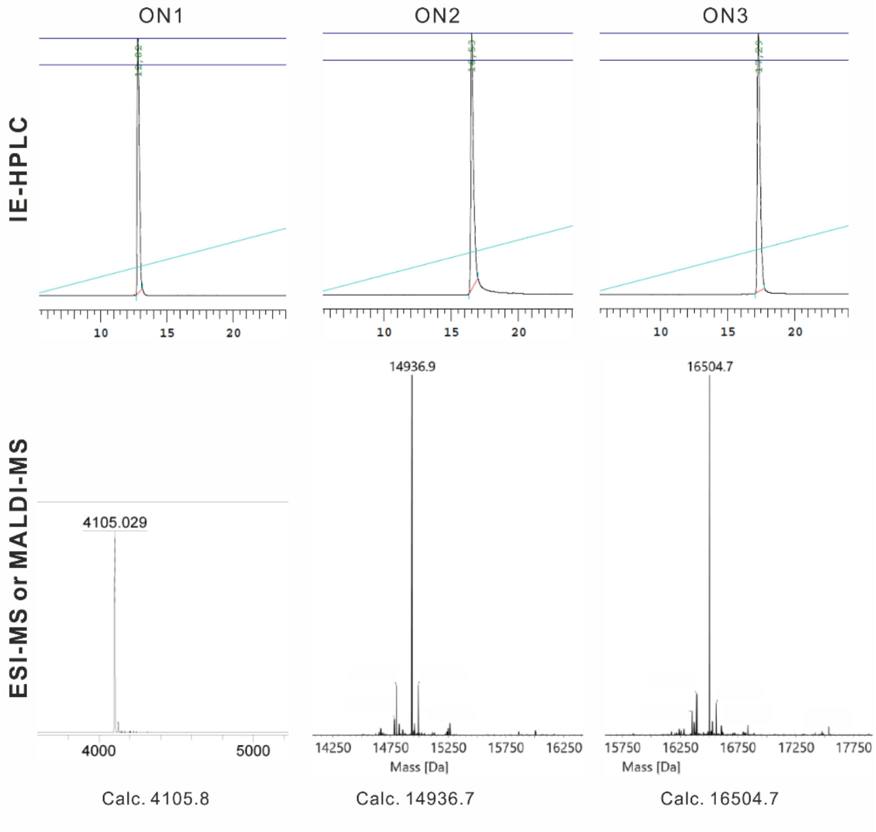

Supplementary Fig. 4. Analytic IE-HPLC trace and ESI-MS (or MALDI-MS) on **ON1**, **ON2** and **ON3**.

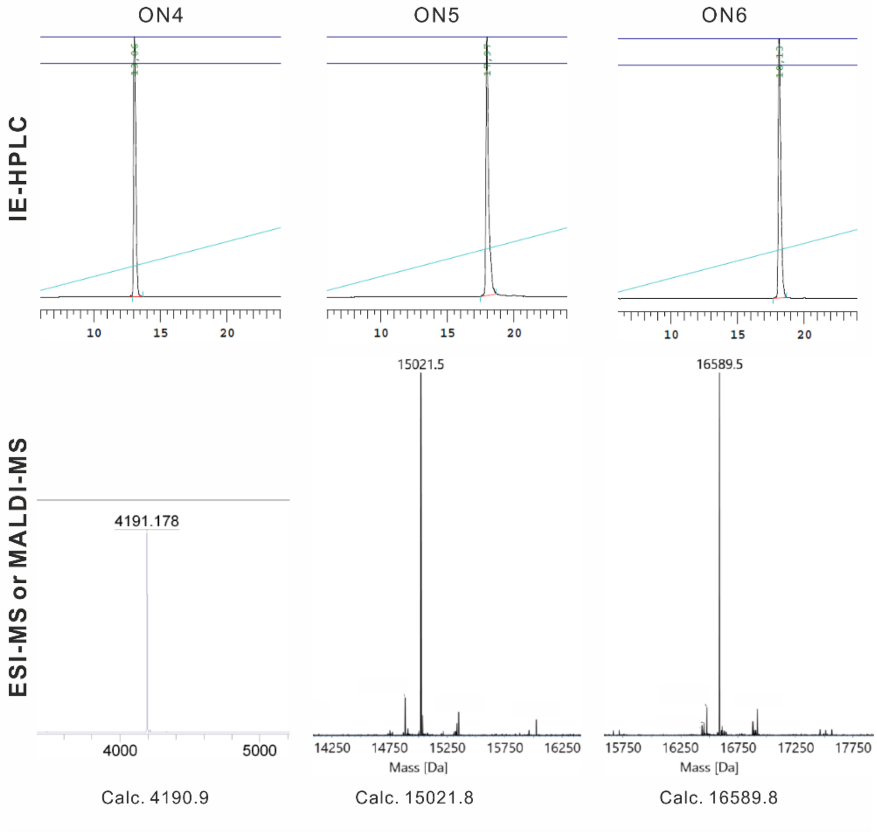

Supplementary Fig. 5. Analytic IE-HPLC trace and ESI-MS (or MALDI-MS) on **ON4**, **ON5** and **ON6**.

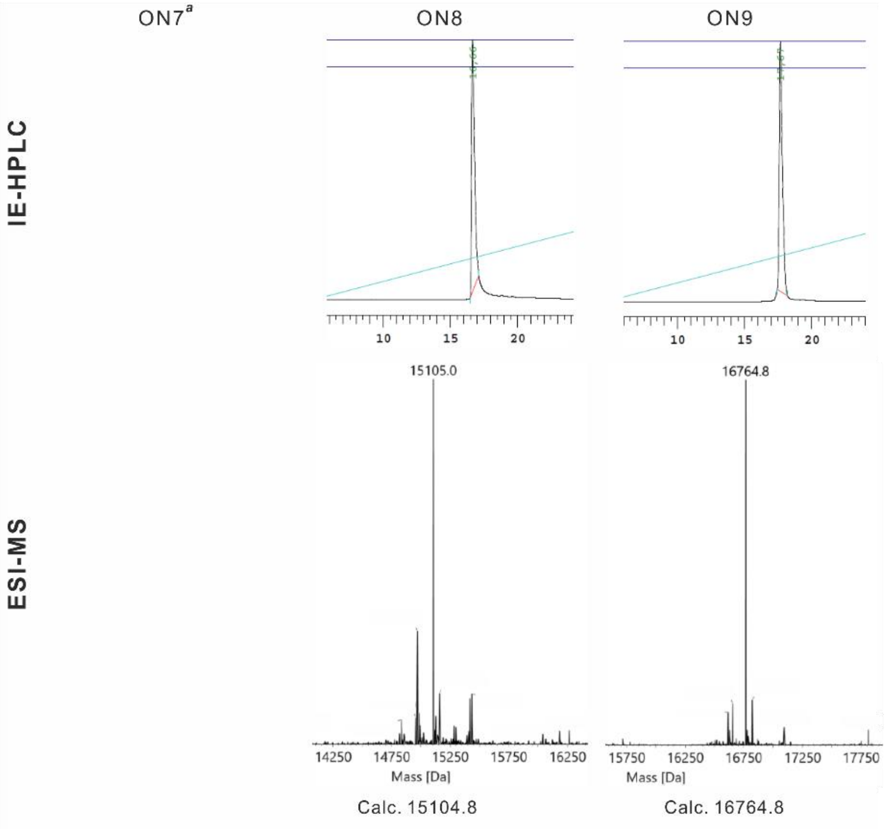

Supplementary Fig. 6. Analytic IE-HPLC trace and ESI-MS (or MALDI-MS) on **ON8** and **ON9**. [a]**ON7** is a known compound and were synthesized following the reported method.[7]

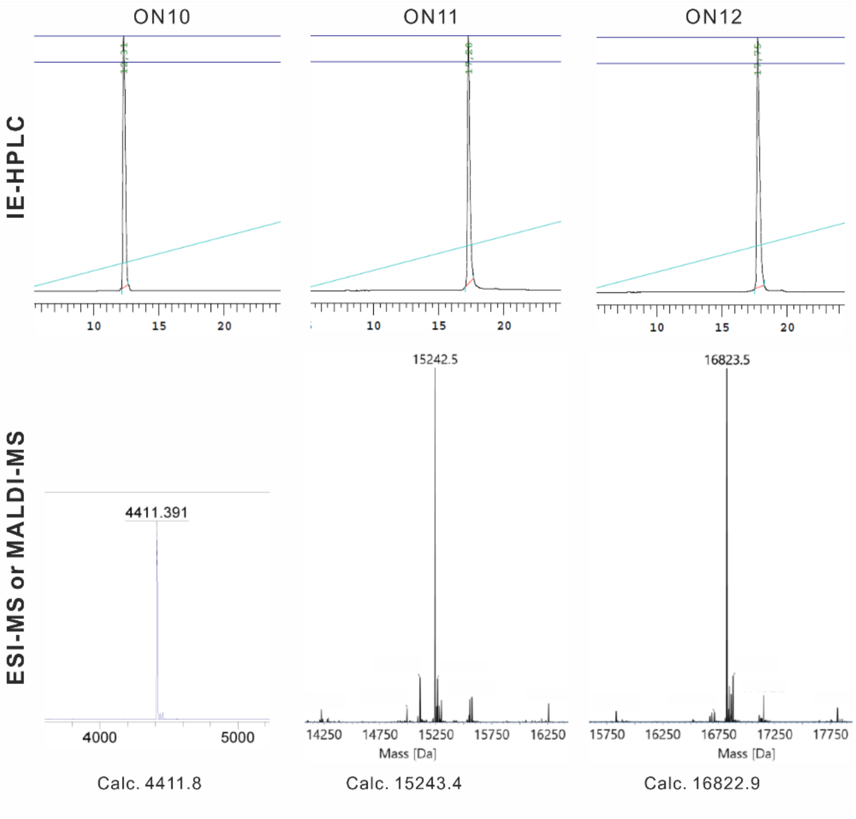

Supplementary Fig. 7. Analytic IE-HPLC trace and ESI-MS (or MALDI-MS) on **ON10**, **ON11** and **ON12**.

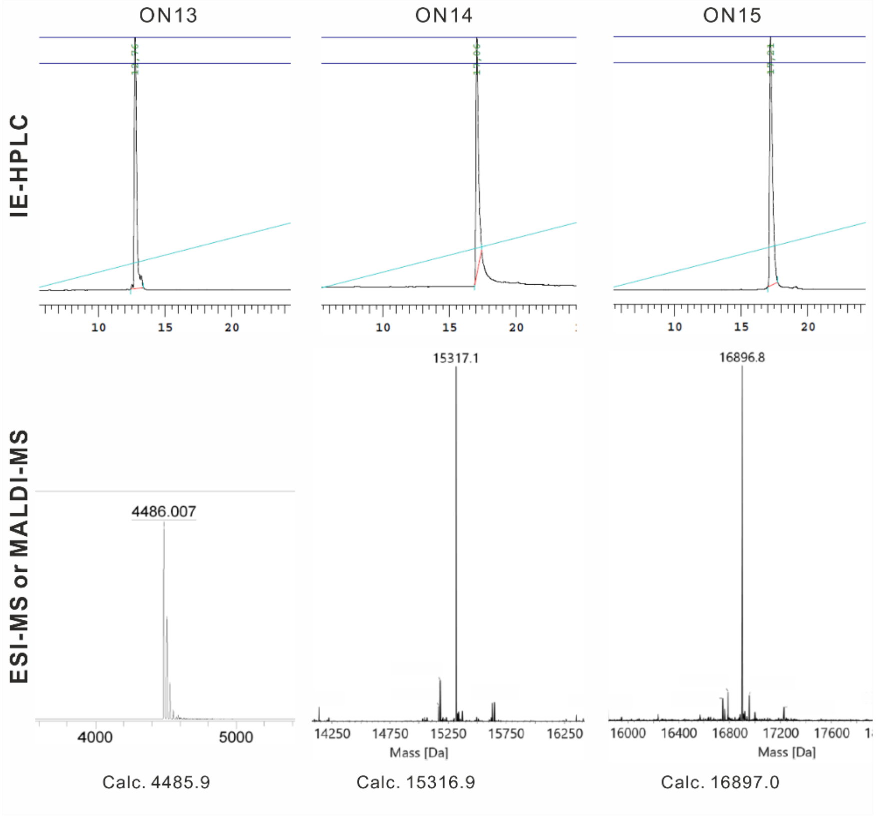

Supplementary Fig. 8. Analytic IE-HPLC trace and ESI-MS (or MALDI-MS) on **ON13**, **ON14** and **ON15**.

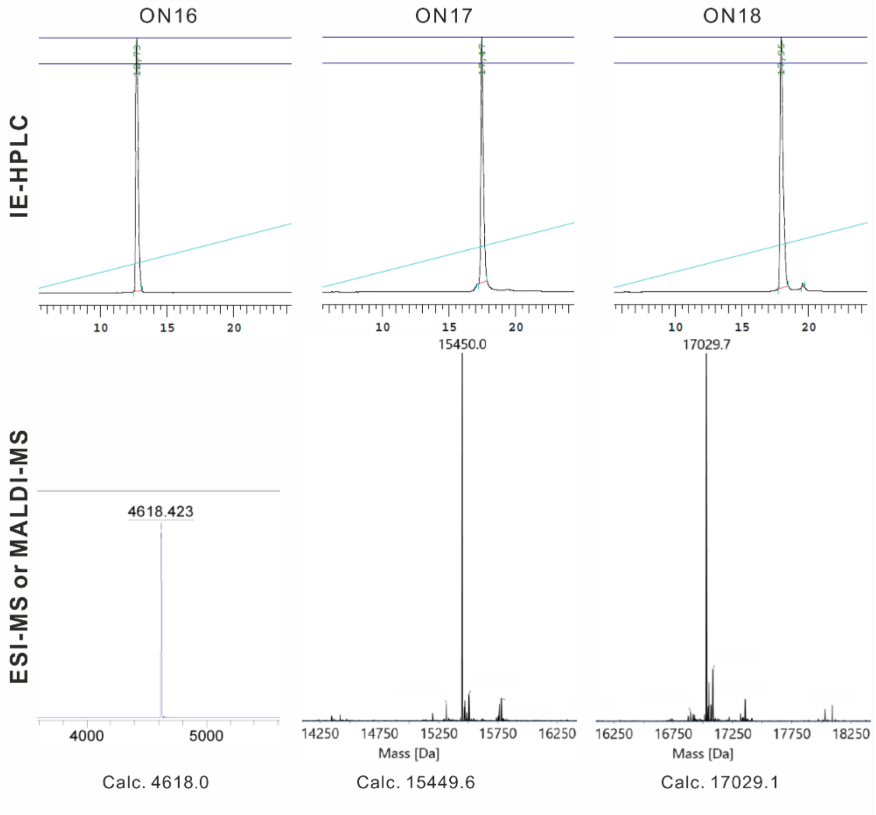

Supplementary Fig. 9. Analytic IE-HPLC trace and ESI-MS (or MALDI-MS) on **ON16**, **ON17** and **ON18**.

A)

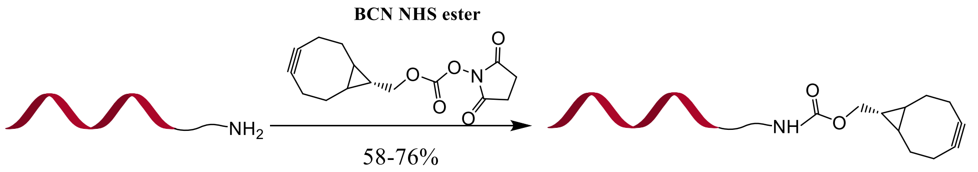

B)

Triplex

ON1, ON4, ON7, ON10, ON13, ON16 3'-TTTTCCCTCTCTC**X**

ON2, ON5, ON8, ON11, ON14, ON17 3'-GTTTATGACAGGAAGATCACATCGTTATGCCGCTAAAAGGGAGAGAG**Y**

ON3, ON6, ON12, ON15, ON18 5'-GGCCGACGCGCTGGGCTACGTCTTGCTGGCTTTACGGCGATTTTCCCTCTCTC**Z**

ON9 **p** = phosphate group 5'-**p**GGCCGACGCGCTGGGCTACGTCTTGCTGGCTTTACGGCGATTTTCCCTCTCTC**Z**

C)

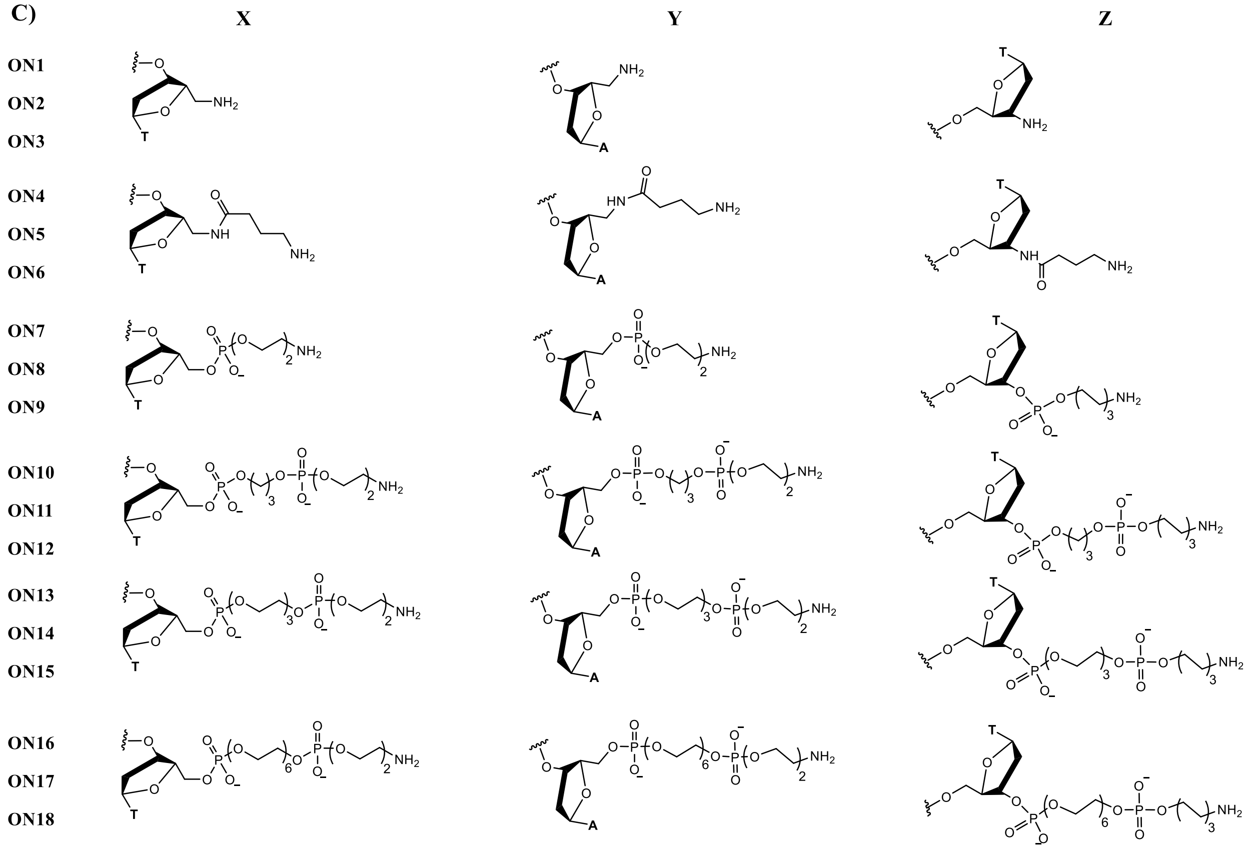

Supplementary Fig. 10. A) BCN-labelling on **ON1-18** in post-ON synthesis: **ON** (63-368 nmol), BCN-NHS ester (30 eq), MeCN/carbonate buffer (1:1), rt, 2 h. B) Sequence of eighteen **ON**s. C) Different linker lengths employed here to furnish the primary amine function either on the 5'-end or on the 3'-end of **ON**s.

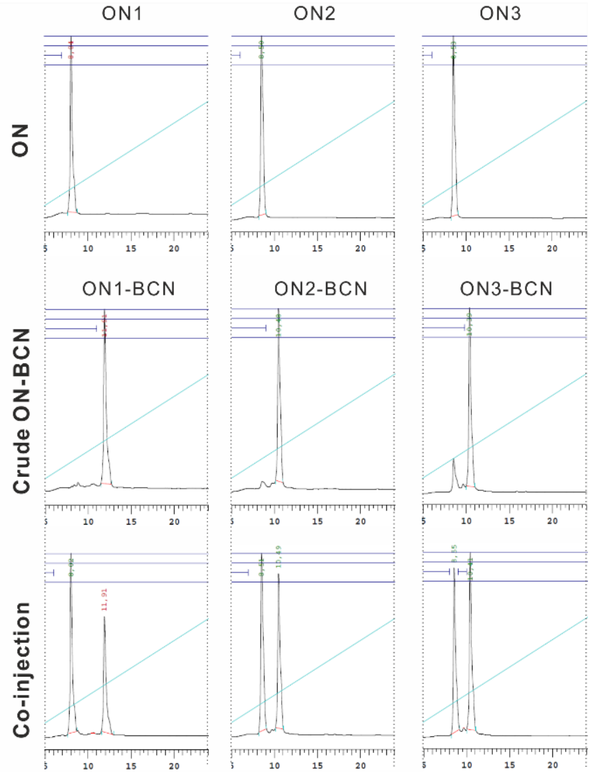

Supplementary Fig. 11. BCN-labelling reactions were monitored *via* analytic RP-HPLC: ON (**ON1**, **ON2** and **ON3**, see top panel), ON-BCN crude products (**ON1-BCN**, **ON2-BCN** and **ON3-BCN**, see middle panel) and co-injection of the corresponding ON and crude ON-BCN (circa 1:1 molar ratio, see bottom panel).

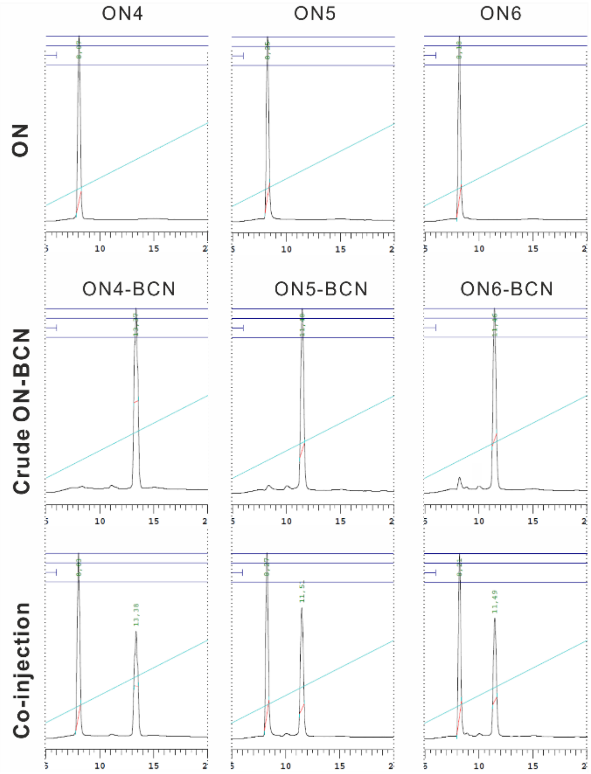

Supplementary Fig. 12. BCN-labelling reactions were monitored *via* analytic RP-HPLC: ON (**ON4**, **ON5** and **ON6**, see top panel), ON-BCN crude products (**ON4-BCN**, **ON5-BCN** and **ON6-BCN**, see middle panel) and co-injection of the corresponding ON and crude ON-BCN (circa 1:1 molar ratio, see bottom panel).

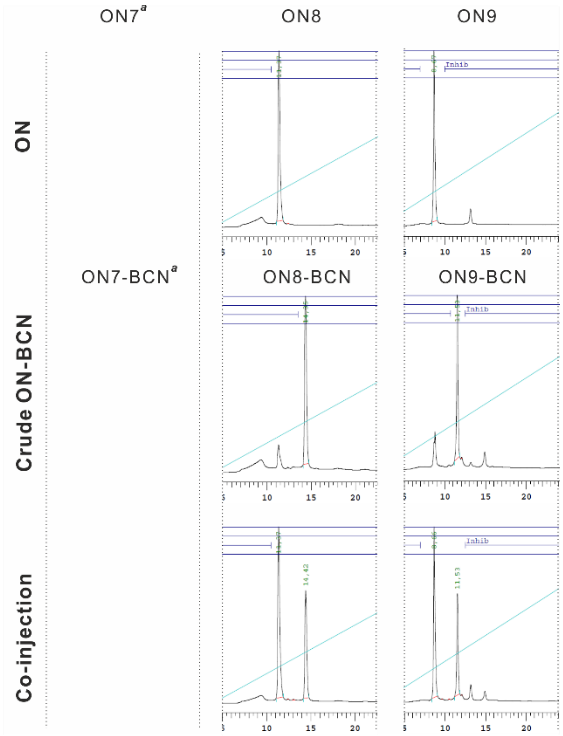

Supplementary Fig. 13. BCN-labelling reactions were monitored *via* analytic RP-HPLC: ON (**ON8** and **ON9**, see top panel), ON-BCN crude products (**ON8-BCN** and **ON9-BCN**, see middle panel) and co-injection of the corresponding ON and crude ON-BCN (circa 1:1 molar ratio, see bottom panel). [a]**ON7** and **ON7-BCN** are known compounds and were synthesized following the reported method.[7]

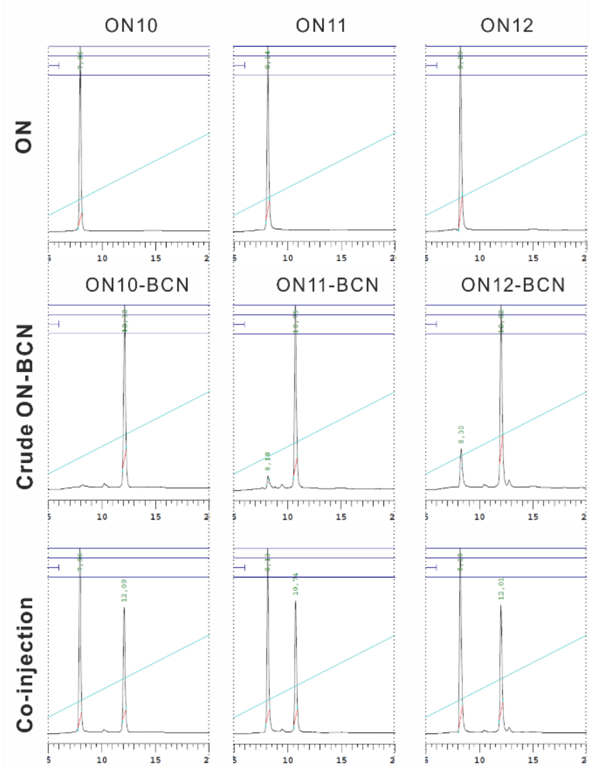

Supplementary Fig. 14. BCN-labelling reactions were monitored *via* analytic RP-HPLC: ON (**ON10**, **ON11** and **ON12**, see top panel), ON-BCN crude products (**ON10-BCN**, **ON11-BCN** and **ON12-BCN**, see middle panel) and co-injection of the corresponding ON and crude ON-BCN (circa 1:1 molar ratio, see bottom panel).

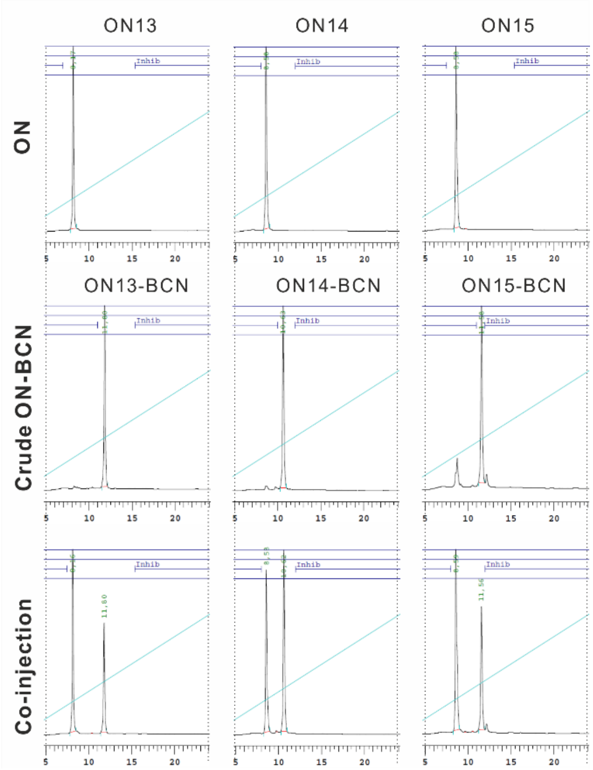

Supplementary Fig. 15. BCN-labelling reactions were monitored *via* analytic RP-HPLC: ON (**ON13**, **ON14** and **ON15**, see top panel), ON-BCN crude products (**ON13-BCN**, **ON14-BCN** and **ON15-BCN**, see middle panel) and co-injection of the corresponding ON and crude ON-BCN (circa 1:1 molar ratio, see bottom panel).

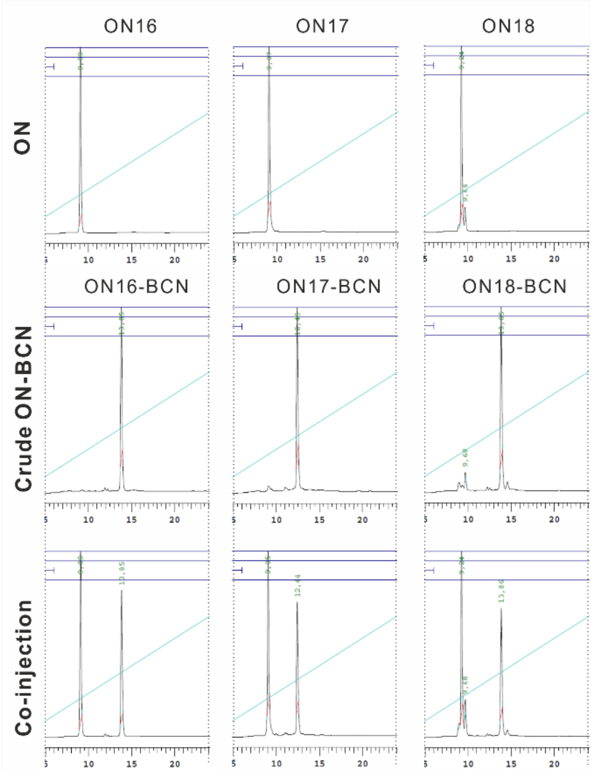

Supplementary Fig. 16. BCN-labelling reactions were monitored *via* analytic RP-HPLC: ON (**ON16**, **ON17** and **ON18**, see top panel), ON-BCN crude products (**ON16-BCN**, **ON17-BCN** and **ON18-BCN**, see middle panel) and co-injection of the corresponding ON and crude ON-BCN (circa 1:1 molar ratio, see bottom panel).

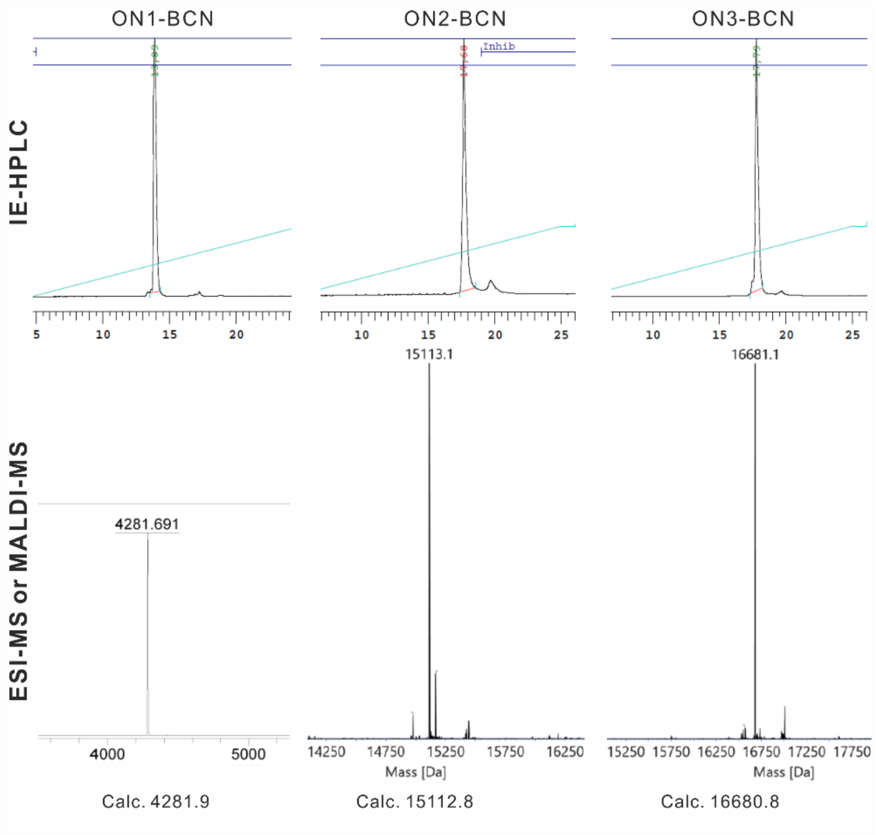

Supplementary Fig. 17. Analytic IE-HPLC trace and ESI-MS (or MALDI-MS) on **ON1-BCN**, **ON2-BCN** and **ON3-BCN**.

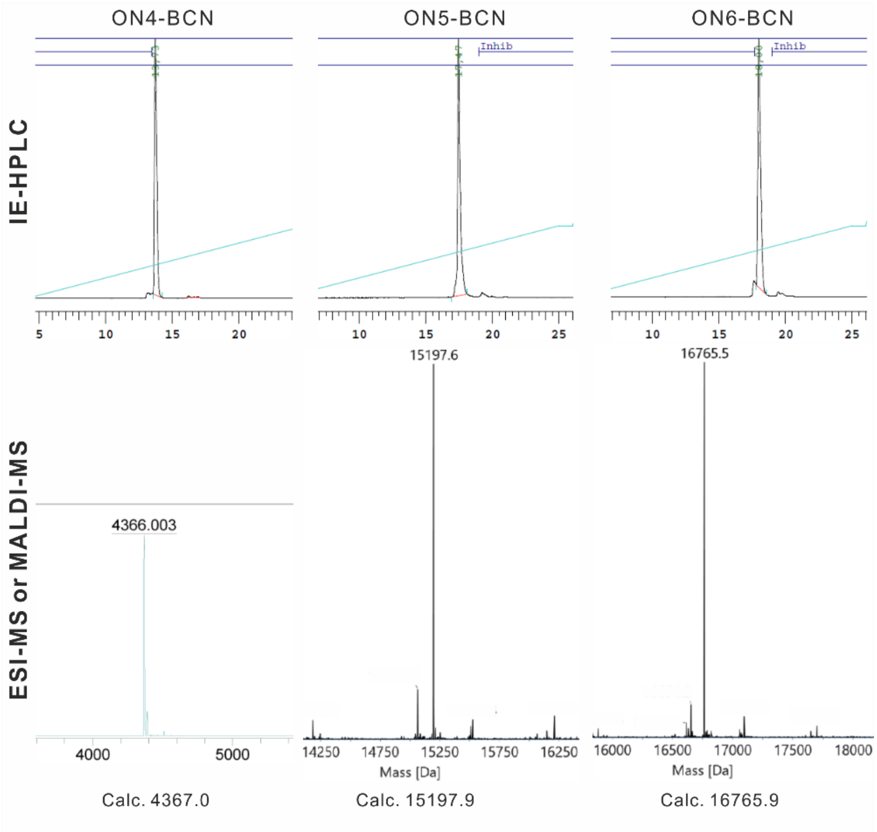

Supplementary Fig. 18. Analytic IE-HPLC trace and ESI-MS (or MALDI-MS) on **ON4-BCN**, **ON5-BCN** and **ON6-BCN**.

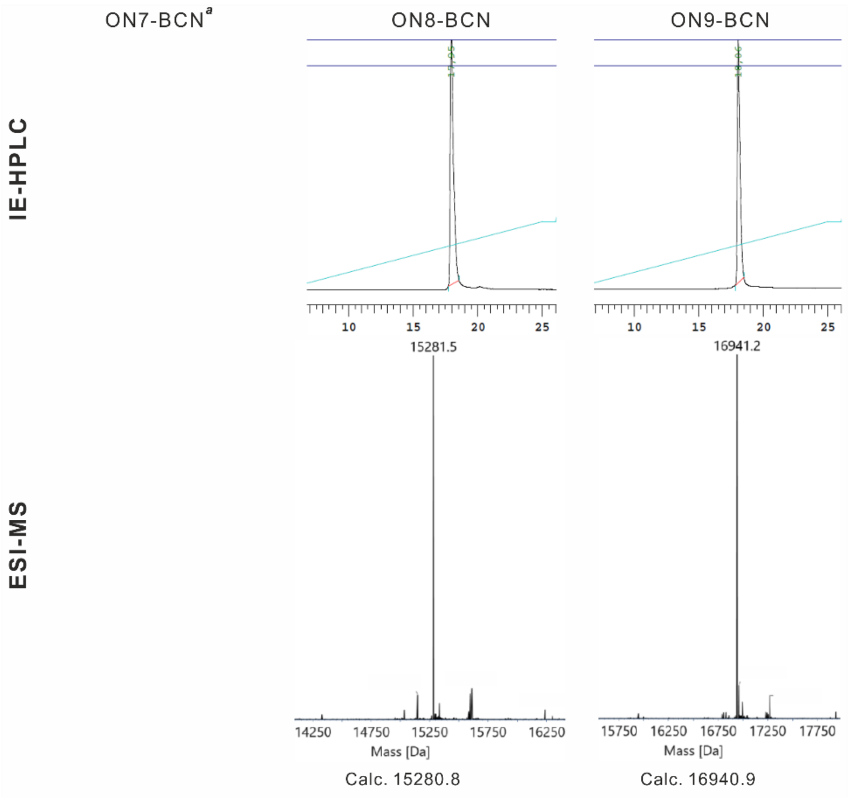

Supplementary Fig. 19. Analytic IE-HPLC trace and ESI-MS (or MALDI-MS) on **ON8-BCN** and **ON9-BCN**. [a]**ON7-BCN** is a known compound and were synthesized following the reported method.[7]

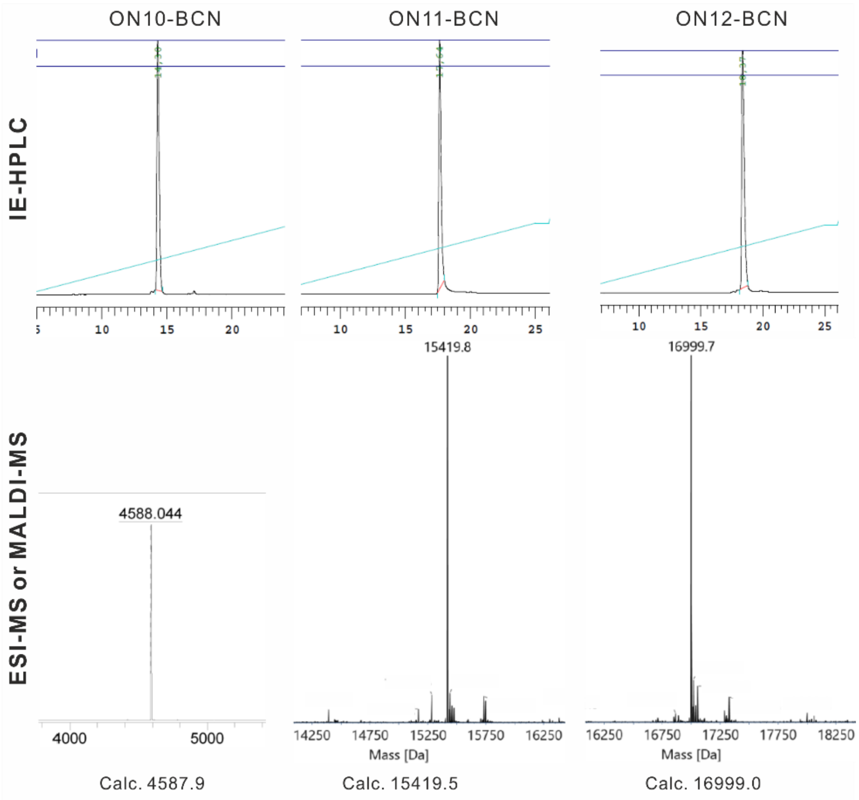

Supplementary Fig. 20. Analytic IE-HPLC trace and ESI-MS (or MALDI-MS) on **ON10-BCN**, **ON11-BCN** and **ON12-BCN**.

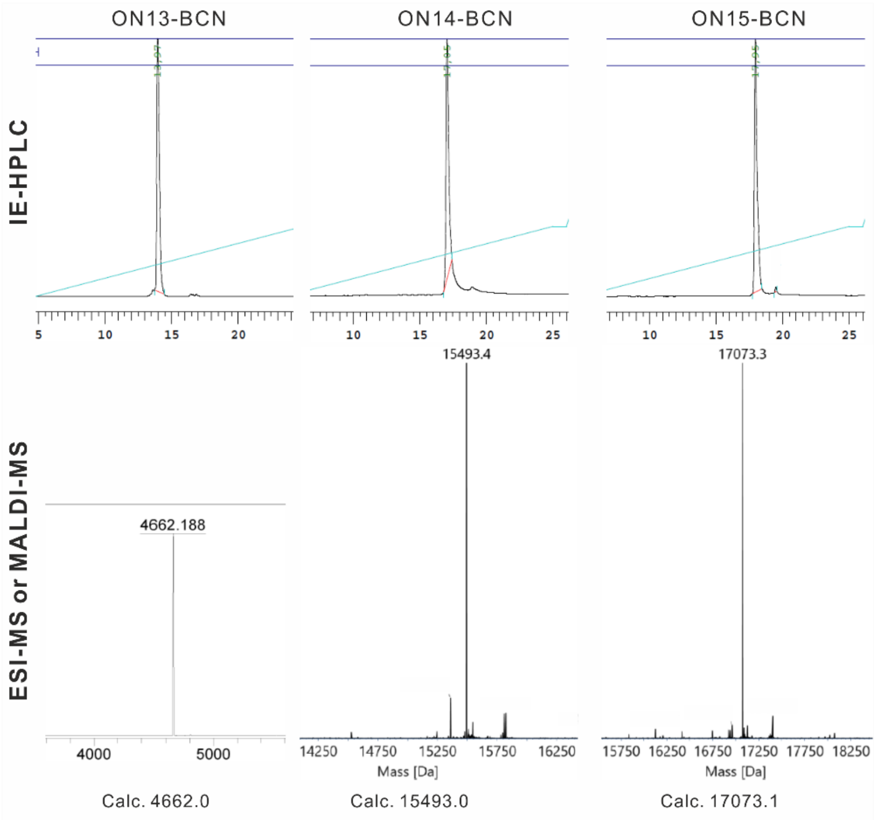

Supplementary Fig. 21. Analytic IE-HPLC trace and ESI-MS (or MALDI-MS) on **ON13-BCN**, **ON14-BCN** and **ON15-BCN**.

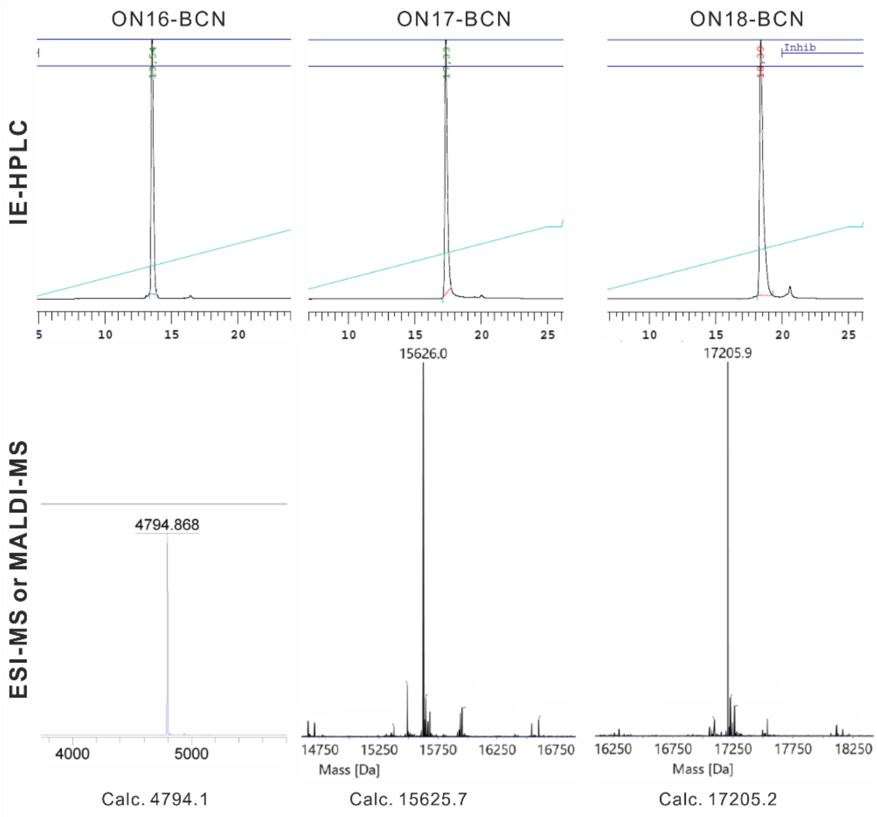

Supplementary Fig. 22. Analytic IE-HPLC trace and ESI-MS (or MALDI-MS) on **ON16-BCN**, **ON17-BCN** and **ON18-BCN**.

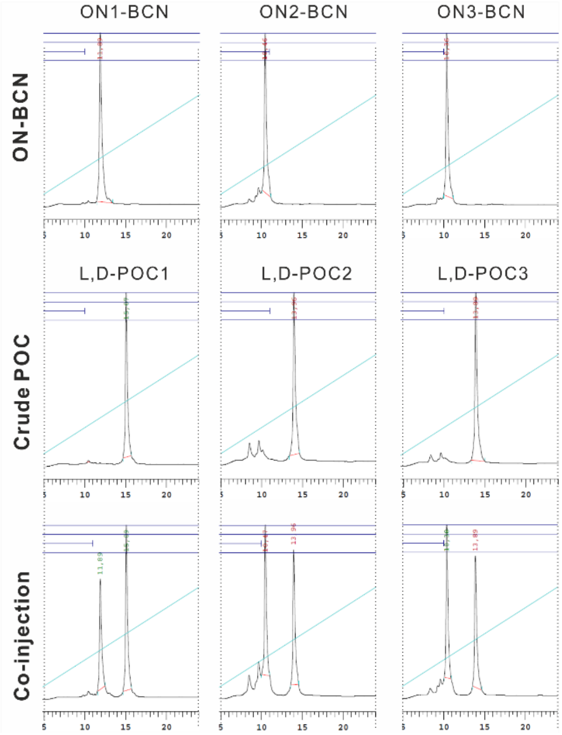

Supplementary Fig. 23. Strain-promoted azide-alkyne cycloaddition reactions between **ON-BCN**s and **L-azidopeptide** was were monitored via analytic RP-HPLC: **ON-BCN** (**ON1-BCN**, **ON2-BCN** and **ON3-BCN**, see top panel), crude POC products (**L,D-POC1**, **L,D-POC2** and **L,D-POC3**, see middle panel) and co-injection of the corresponding D-ON-BCN and crude POC (circa 1:1 molar ratio, see bottom panel).

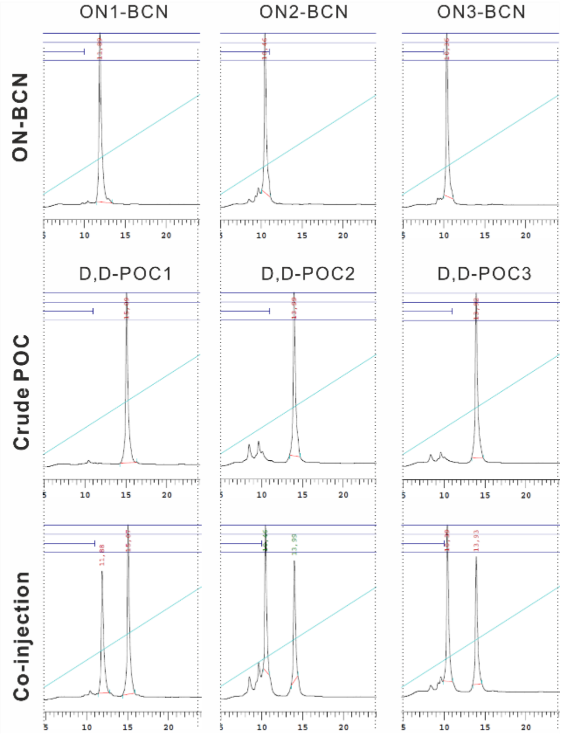

Supplementary Fig. 24. Strain-promoted azide-alkyne cycloaddition reactions between **ON-BCN**s and **D-azidopeptide** was were monitored via analytic RP-HPLC: **ON-BCN** (**ON1-BCN**, **ON2-BCN** and **ON3-BCN**, see top panel), crude POC products (**D,D-POC1**, **D,D-POC2** and **D,D-POC3**, see middle panel) and co-injection of the corresponding D-ON-BCN and crude POC (circa 1:1 molar ratio, see bottom panel).

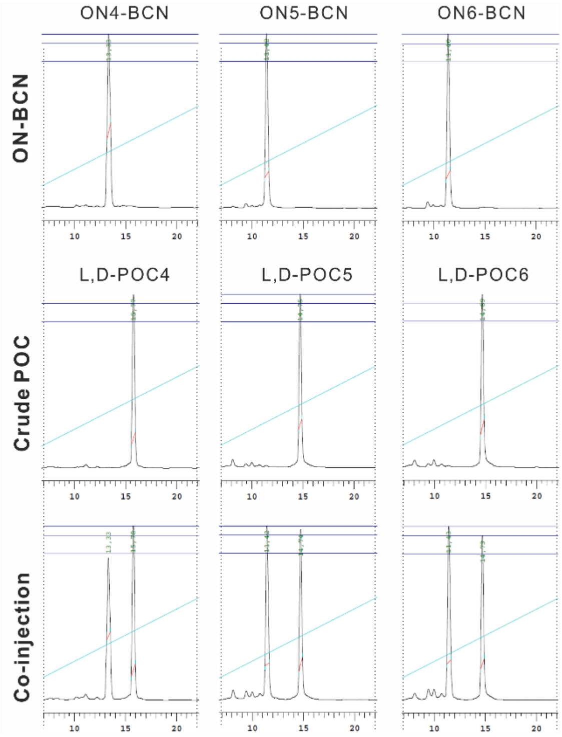

Supplementary Fig. 25. Strain-promoted azide-alkyne cycloaddition reactions between **ON-BCN**s and **L-azidopeptide** was were monitored via analytic RP-HPLC: **ON-BCN** (**ON4-BCN**, **ON5-BCN** and **ON6-BCN**, see top panel), crude POC products (**L,D-POC4**, **L,D-POC5** and **L,D-POC6**, see middle panel) and co-injection of the corresponding D-ON-BCN and crude POC (circa 1:1 molar ratio, see bottom panel).

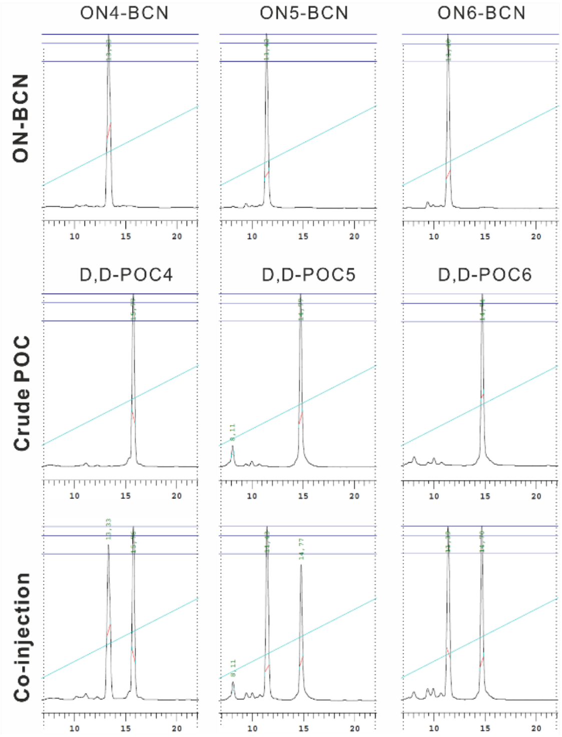

Supplementary Fig. 26. Strain-promoted azide-alkyne cycloaddition reactions between **ON-BCN**s and **D-azidopeptide** was were monitored via analytic RP-HPLC: **ON-BCN** (**ON4-BCN**, **ON5-BCN** and **ON6-BCN**, see top panel), crude POC products (**D,D-POC4**, **D,D-POC5** and **D,D-POC6**, see middle panel) and co-injection of the corresponding D-ON-BCN and crude POC (circa 1:1 molar ratio, see bottom panel).

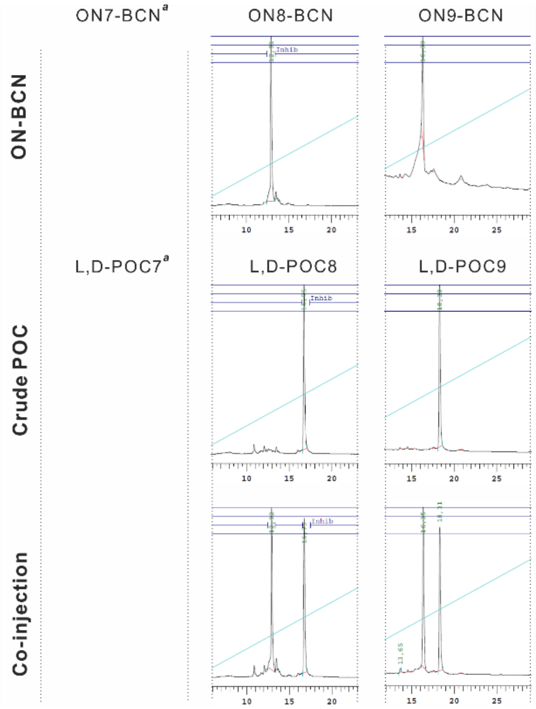

Supplementary Fig. 27. Strain-promoted azide-alkyne cycloaddition reactions between **ON-BCN**s and **L-azidopeptide** was were monitored via analytic RP-HPLC: **ON-BCN** (**ON7-BCN**, **ON8-BCN** and **ON9-BCN**, see top panel), crude POC products (**L,D-POC7**, **L,D-POC8** and **L,D-POC9**, see middle panel) and co-injection of the corresponding D-ON-BCN and crude POC (circa 1:1 molar ratio, see bottom panel). [a]**ON7-BCN** and **L,D-POC7** are known compounds and were synthesized following the reported method.[7]

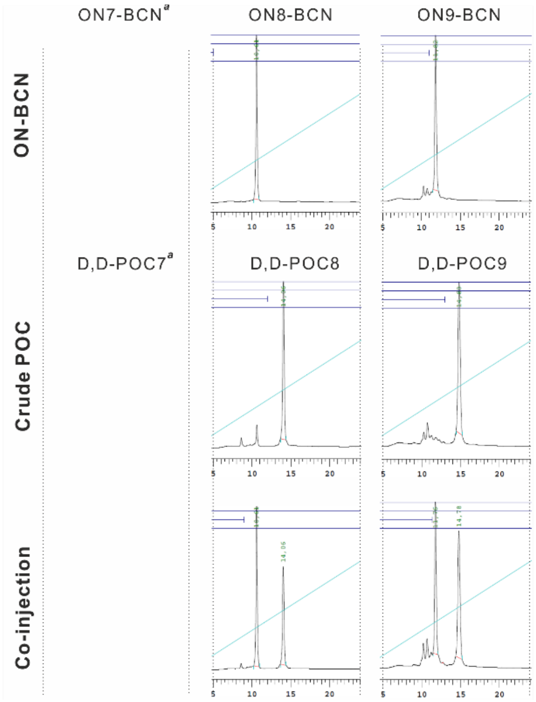

Supplementary Fig. 28. Strain-promoted azide-alkyne cycloaddition reactions between **ON-BCN**s and **D-azidopeptide** was were monitored via analytic RP-HPLC: **ON-BCN** (**ON7-BCN**, **ON8-BCN** and **ON9-BCN**, see top panel), crude POC products (**D,D-POC7**, **D,D-POC8** and **D,D-POC9**, see middle panel) and co-injection of the corresponding D-ON-BCN and crude POC (circa 1:1 molar ratio, see bottom panel). [a]**ON7-BCN** and **D,D-POC7** are known compounds and were synthesized following the reported method.[7]

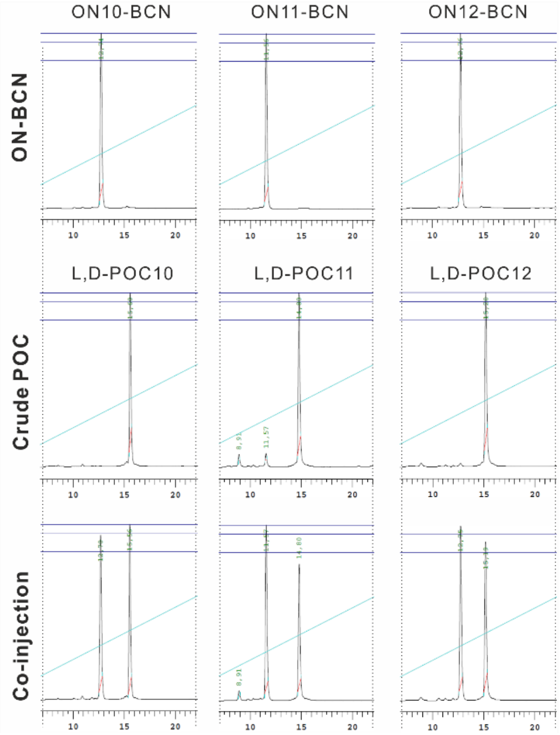

Supplementary Fig. 29. Strain-promoted azide-alkyne cycloaddition reactions between **ON-BCN**s and **L-azidopeptide** was were monitored via analytic RP-HPLC: **ON-BCN** (**ON10-BCN**, **ON11-BCN** and **ON12-BCN**, see top panel), crude POC products (**L,D-POC10**, **L,D-POC12** and **L,D-POC13**, see middle panel) and co-injection of the corresponding D-ON-BCN and crude POC (circa 1:1 molar ratio, see bottom panel).

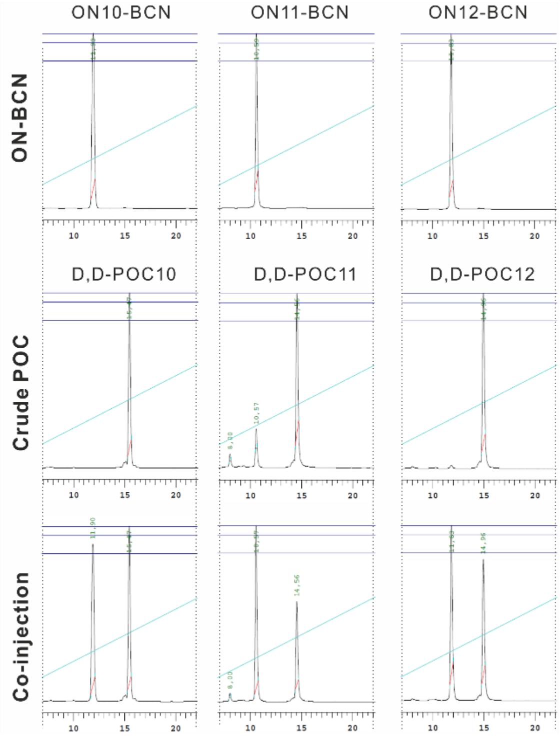

Supplementary Fig. 30. Strain-promoted azide-alkyne cycloaddition reactions between **ON-BCN**s and **D-azidopeptide** was were monitored via analytic RP-HPLC: **ON-BCN** (**ON10-BCN**, **ON11-BCN** and **ON12-BCN**, see top panel), crude POC products (**D,D-POC10**, **D,D-POC12** and **D,D-POC13**, see middle panel) and co-injection of the corresponding D-ON-BCN and crude POC (circa 1:1 molar ratio, see bottom panel).

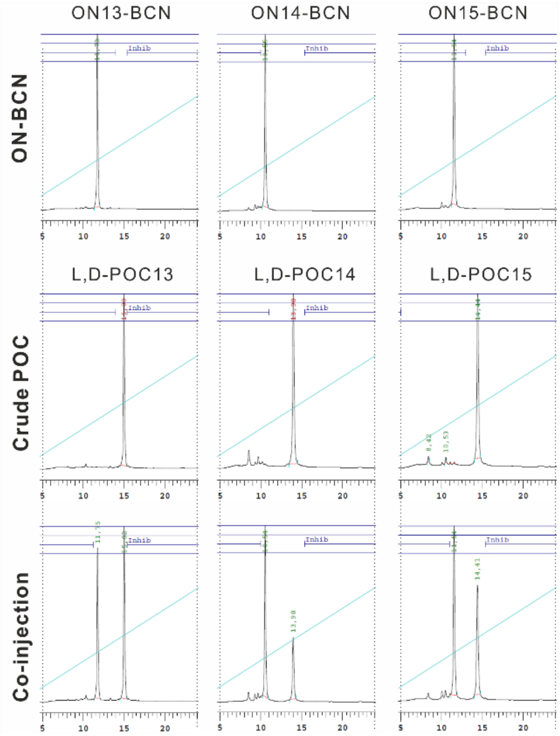

Supplementary Fig. 31. Strain-promoted azide-alkyne cycloaddition reactions between **ON-BCN**s and **L-azidopeptide** was were monitored via analytic RP-HPLC: **ON-BCN** (**ON13-BCN**, **ON14-BCN** and **ON15-BCN**, see top panel), crude POC products (**L,D-POC13**, **L,D-POC14** and **L,D-POC15**, see middle panel) and co-injection of the corresponding D-ON-BCN and crude POC (circa 1:1 molar ratio, see bottom panel).

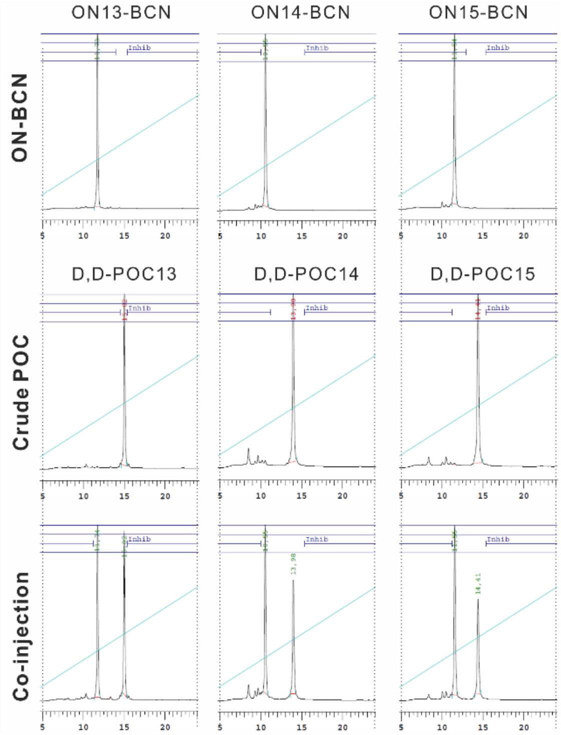

Supplementary Fig. 32. Strain-promoted azide-alkyne cycloaddition reactions between **ON-BCN**s and **D-azidopeptide** was were monitored via analytic RP-HPLC: **ON-BCN** (**ON13-BCN**, **ON14-BCN** and **ON15-BCN**, see top panel), crude POC products (**D,D-POC13**, **D,D-POC14** and **D,D-POC15**, see middle panel) and co-injection of the corresponding D-ON-BCN and crude POC (circa 1:1 molar ratio, see bottom panel).

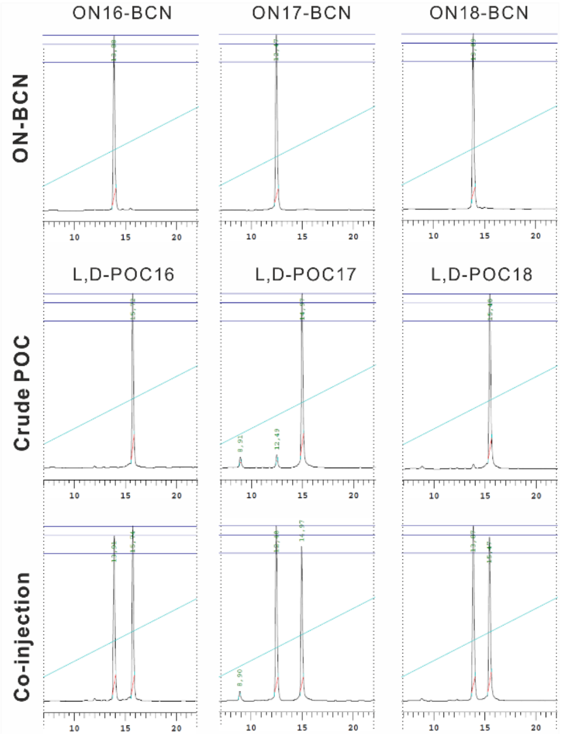

Supplementary Fig. 33. Strain-promoted azide-alkyne cycloaddition reactions between **ON-BCN**s and **L-azidopeptide** was were monitored via analytic RP-HPLC: **ON-BCN** (**ON16-BCN**, **ON17-BCN** and **ON18-BCN**, see top panel), crude POC products (**L,D-POC16**, **L,D-POC17** and **L,D-POC18**, see middle panel) and co-injection of the corresponding D-ON-BCN and crude POC (circa 1:1 molar ratio, see bottom panel).

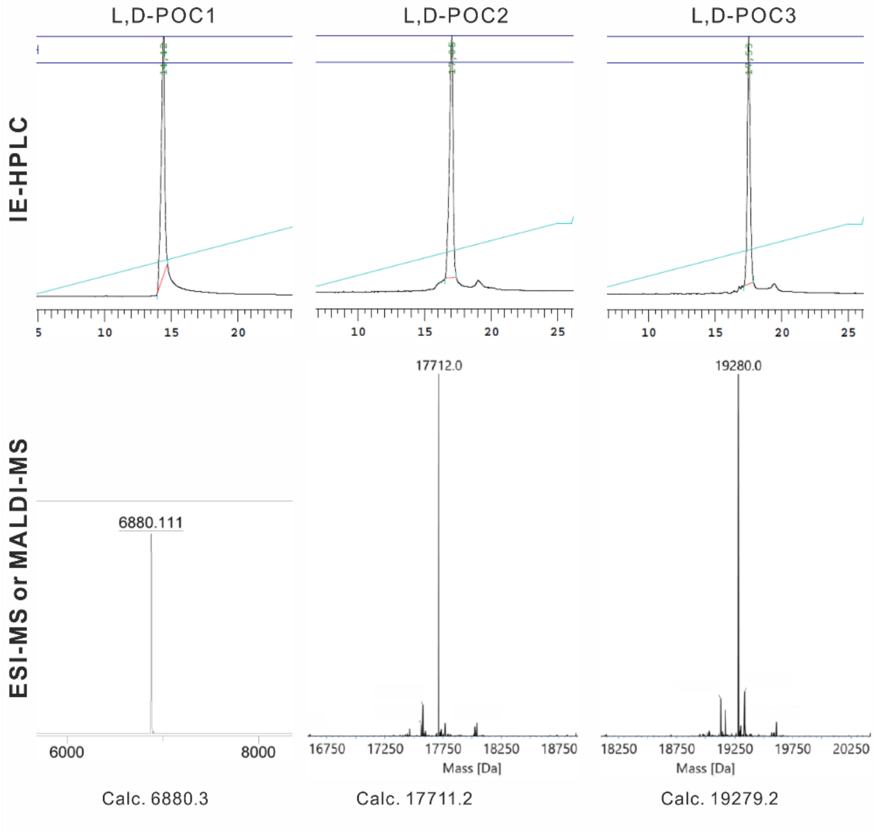

Supplementary Fig. 34. Analytic IE-HPLC trace and ESI-MS (or MALDI-MS) on **L,D-POC1**, **L,D-POC2** and **L,D-POC3**.

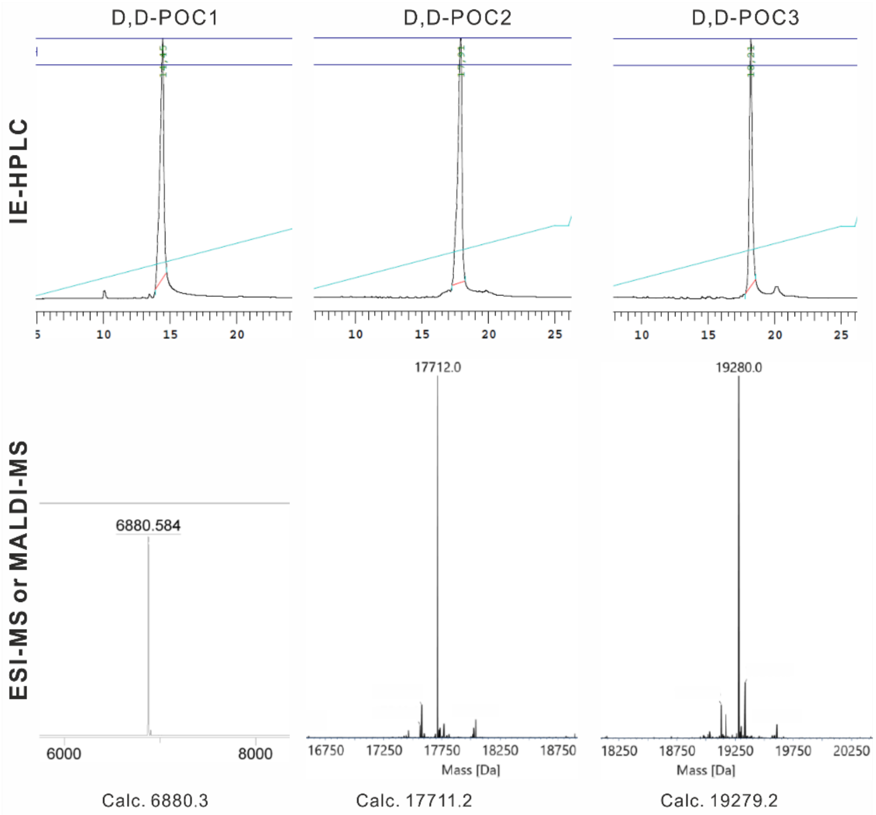

Supplementary Fig. 35. Analytic IE-HPLC trace and ESI-MS (or MALDI-MS) on **D,D-POC1**, **D,D-POC2** and **D,D-POC3**.

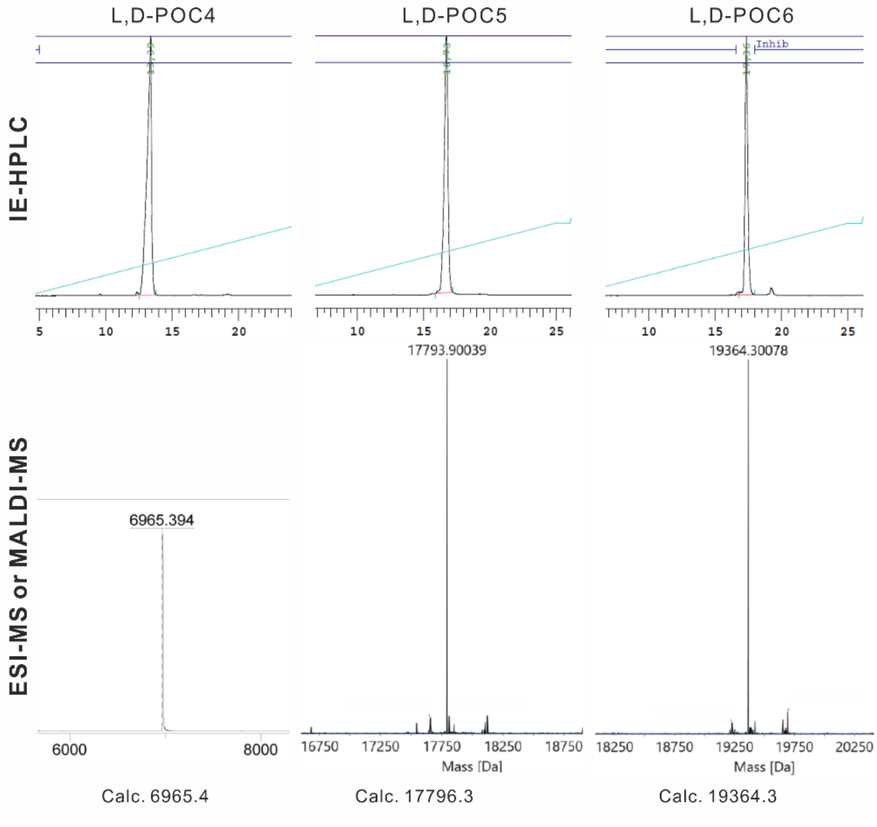

Supplementary Fig. 36. Analytic IE-HPLC trace and ESI-MS (or MALDI-MS) on **L,D-POC4**, **L,D-POC5** and **L,D-POC6**.

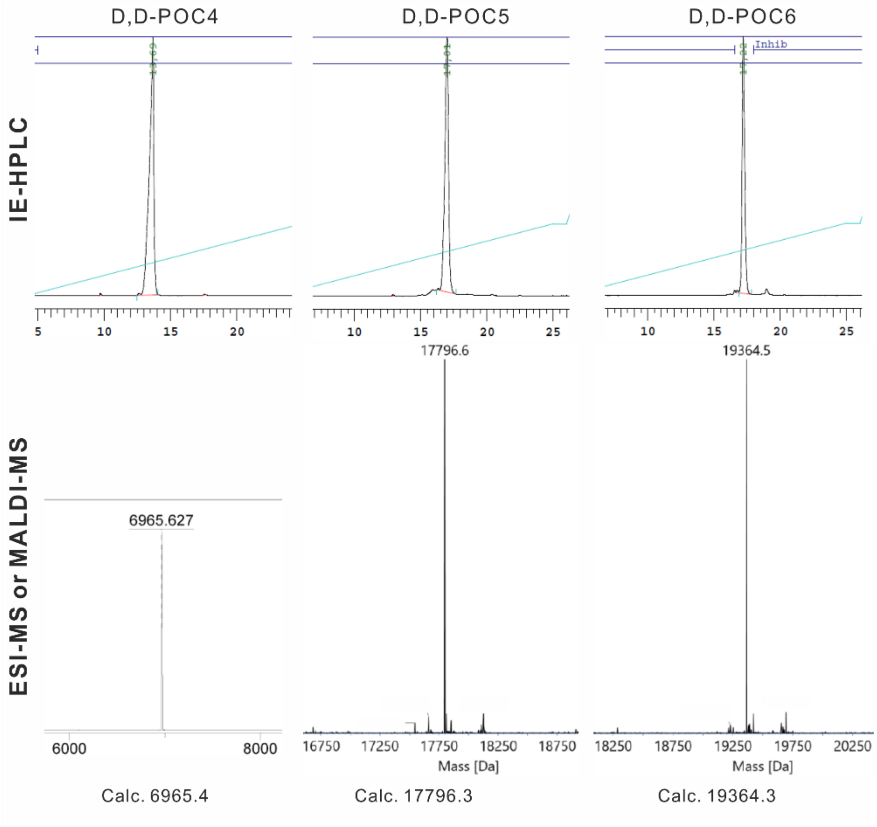

Supplementary Fig. 37. Analytic IE-HPLC trace and ESI-MS (or MALDI-MS) on **D,D-POC4**, **D,D-POC5** and **D,D-POC6**.

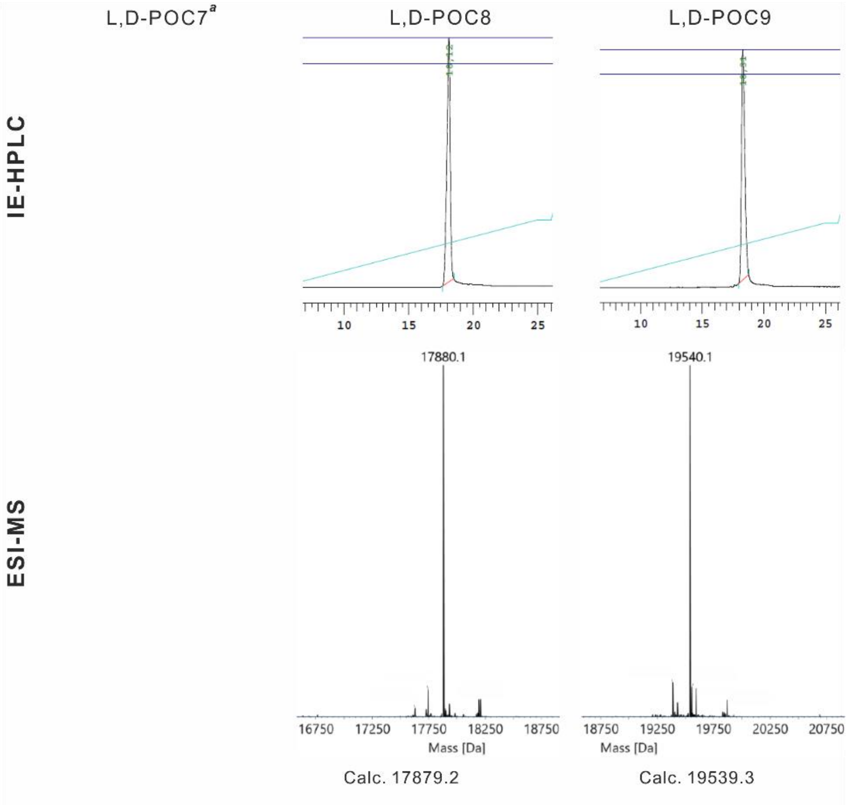

Supplementary Fig. 38. Analytic IE-HPLC trace and ESI-MS on **L,D-POC7**, **L,D-POC8** and **L,D-POC9**. [a]**L,D-POC7** is a known compound and was synthesized following the reported method.[7]

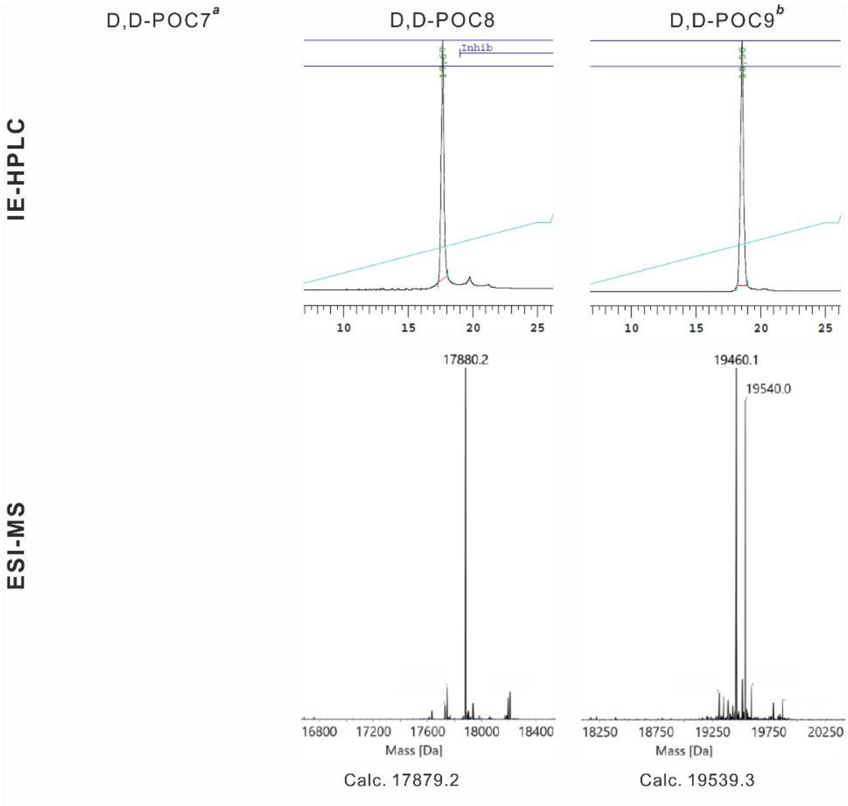

Supplementary Fig. 39. Analytic IE-HPLC trace and ESI-MS on **D,D-POC7**, **D,D-POC8** and **D,D-POC9**. [a]**D,D-POC7** is a known compound and was synthesized following the reported method.[7] [b] The found main peak (19460.1) for **D,D-POC9** is interpreted as the loss of 5'-phophate group (-80 Da).

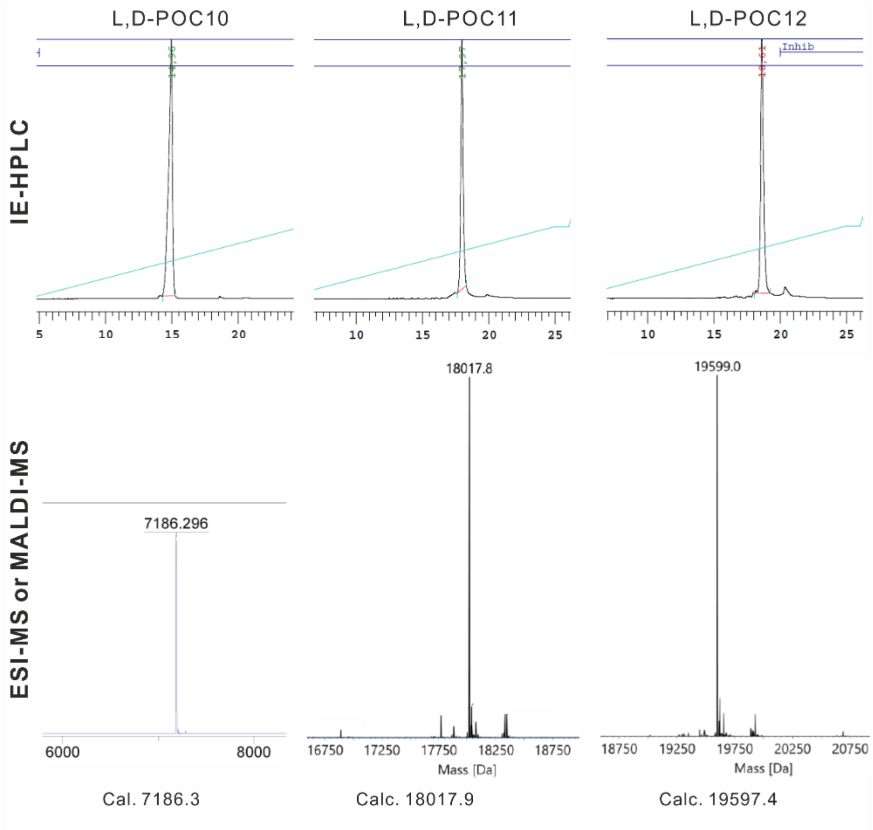

Supplementary Fig. 40. Analytic IE-HPLC trace and ESI-MS (or MALDI-MS) on **L,D-POC10**, **L,D-POC11** and **L,D-POC12**.

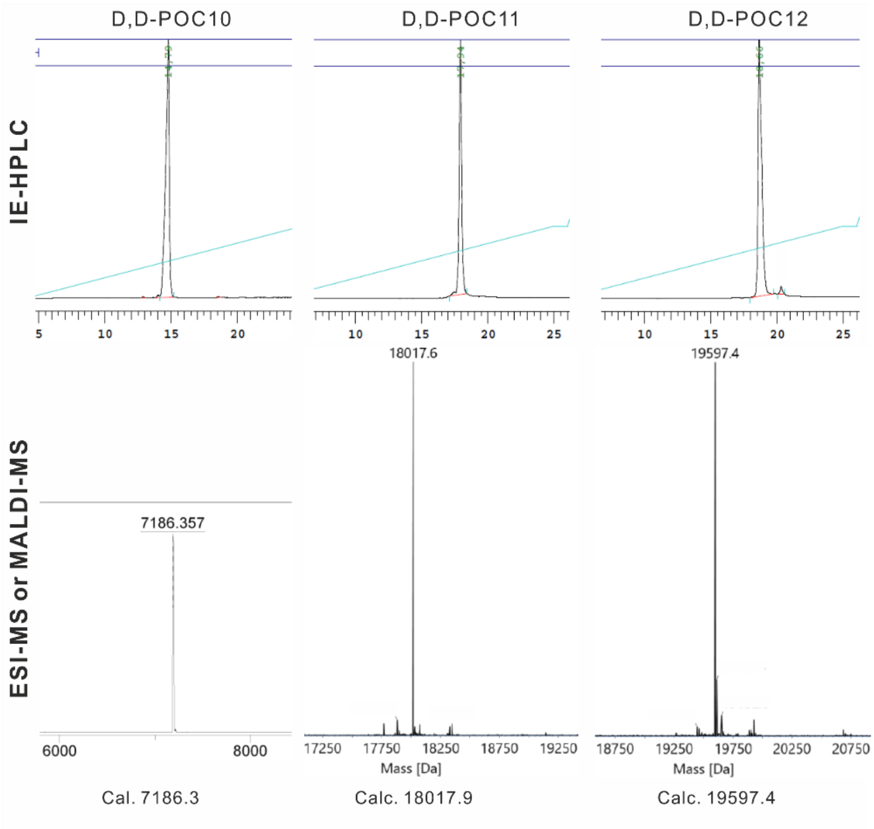

Supplementary Fig. 41. Analytic IE-HPLC trace and ESI-MS (or MALDI-MS) on **D,D-POC10**, **D,D-POC11** and **D,D-POC12**.

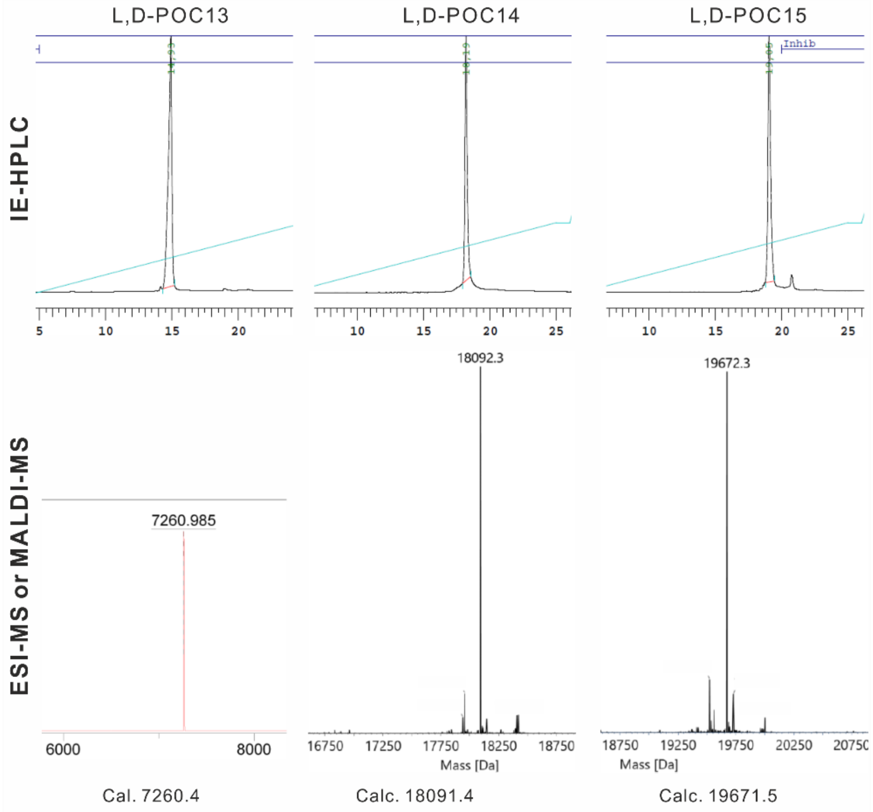

Supplementary Fig. 42. Analytic IE-HPLC trace and ESI-MS (or MALDI-MS) on **L,D-POC13**, **L,D-POC14** and **L,D-POC15**.

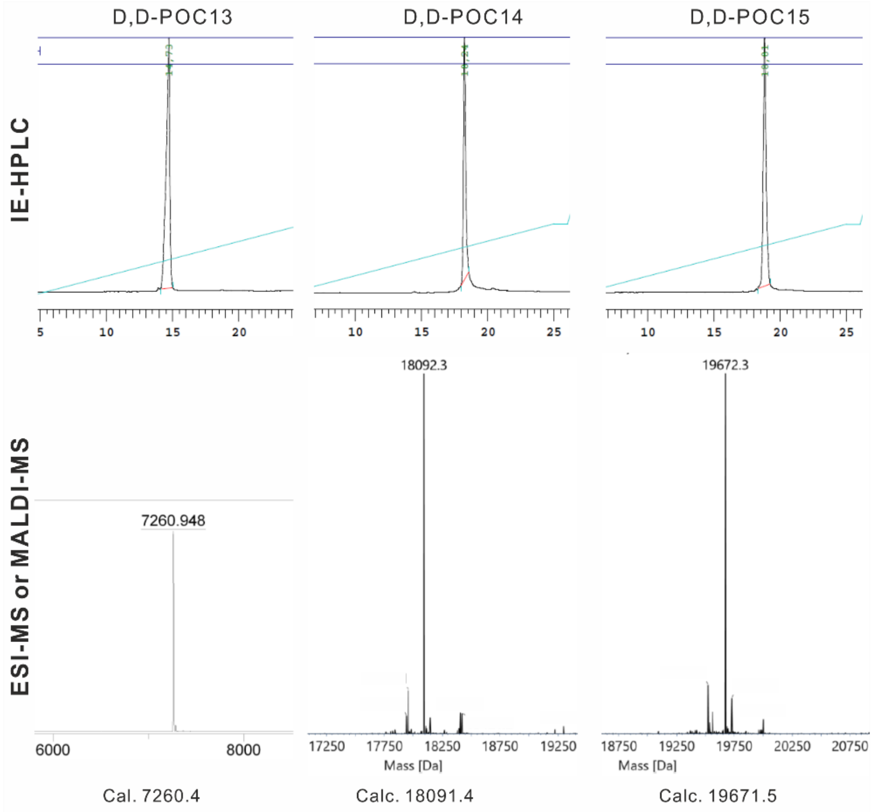

Supplementary Fig. 43. Analytic IE-HPLC trace and ESI-MS (or MALDI-MS) on **D,D-POC13**, **D,D-POC14** and **D,D-POC15**.

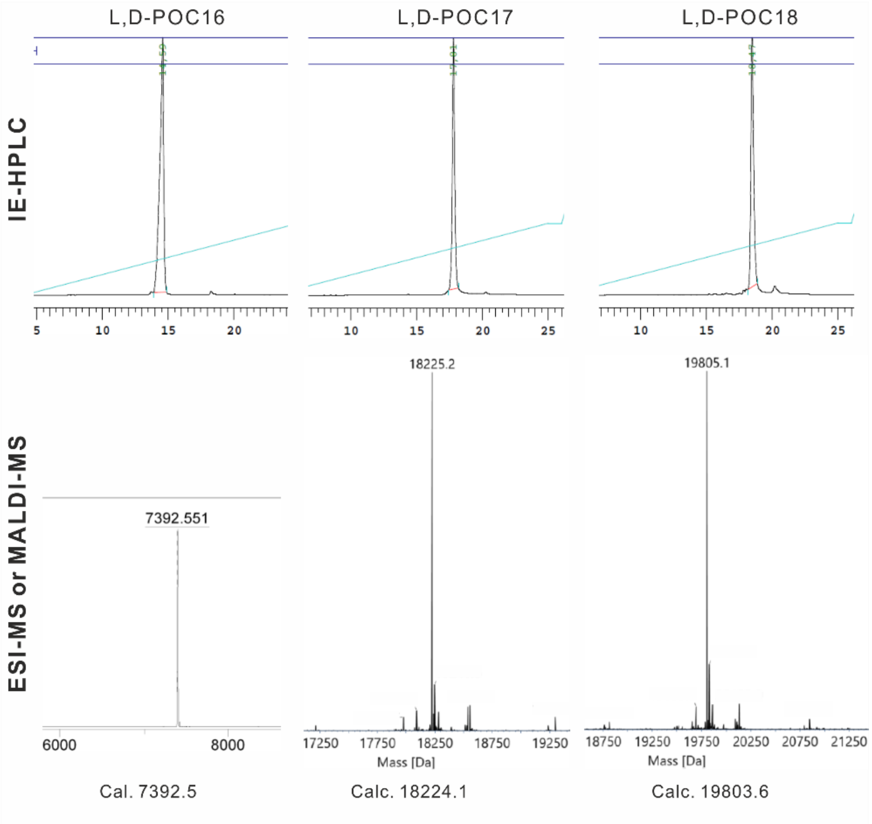

Supplementary Fig. 44. Analytic IE-HPLC trace and ESI-MS (or MALDI-MS) on **L,D-POC16**, **L,D-POC17** and **L,D-POC18**.

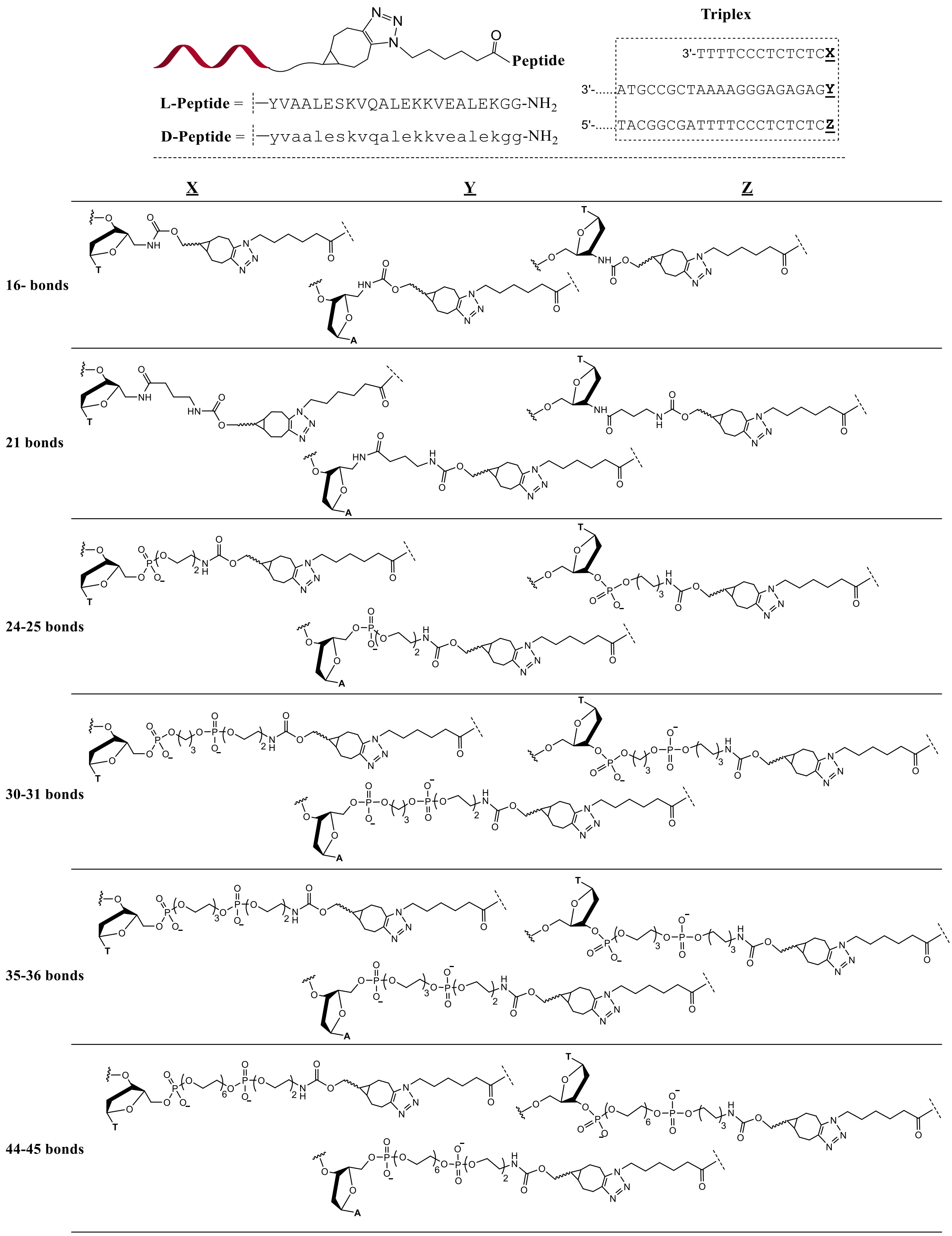

Supplementary Fig. 45. Different interdomain linkers between the ON triplex template and the trimeric peptide coiled-coil domain used in this study. The interdomain distances were calculated as the shortest path from 5'-/3'-position (5'-NH/3'-NH or 5'-O/3'-) of the oligonucleotide triplex to the N-termini (NH of Tyr) of the peptide. Y, V, L, E, S, K, Q, A, G are L-amino acids whereas y, v, l, e, s, k, q, a, g are D-amino acids. A, G, C and T are natural DNA monomers.

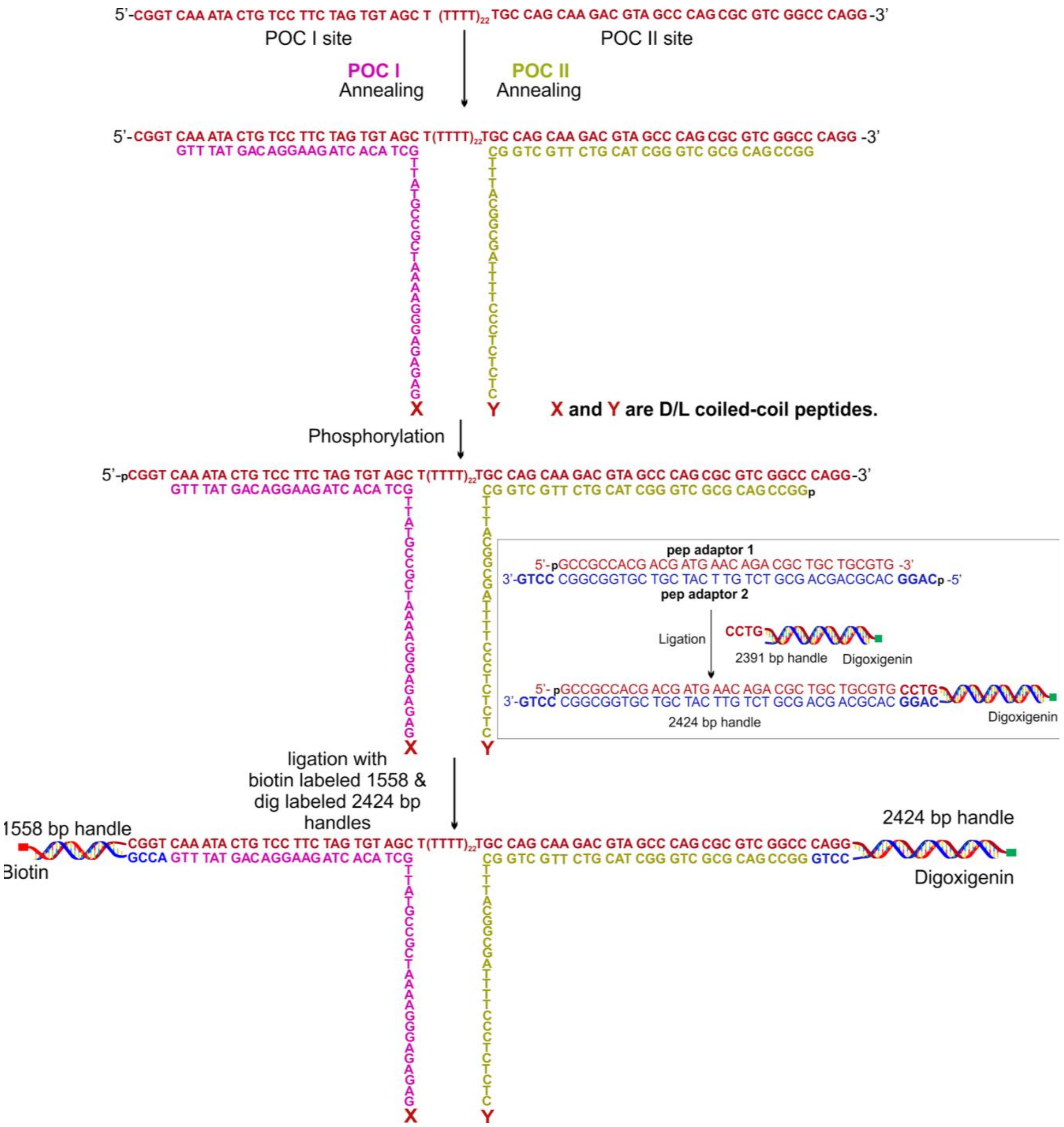

Supplementary Fig. 46. Flow chart of the synthesis strategy of the coiled-coil peptide constructs.

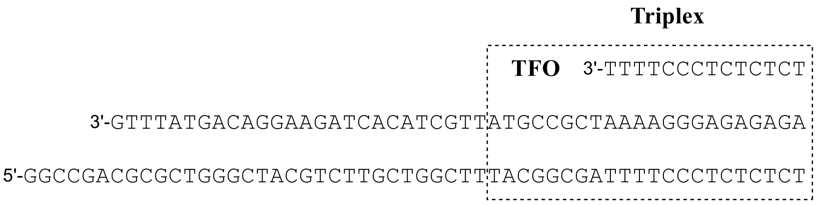

Supplementary Fig. 47. The three ONs used to assemble the DNA duplex and triplex controls (Figure 3A and 3D).

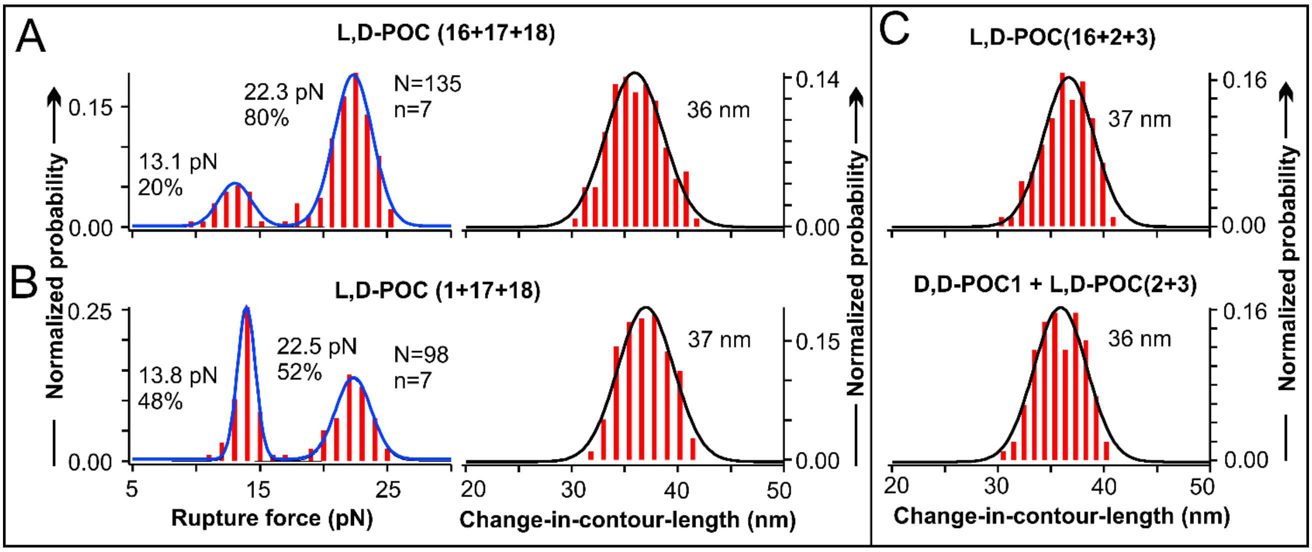

Supplementary Fig. 48 A) Rupture force histogram for the L-peptide, D-DNA (L,D-POC(16+17), 44-45 bonds) bound with a TFO conjugated with L-peptide (L,D-POC18, 44-45 bonds, top left) and corresponding change-in-contour-length histogram (top right). **B)** Rupture force histogram for the L-peptide, D-DNA (L,D-POC(17+18), 44-45 bonds) bound with a TFO conjugated with L-peptide (L,D-POC1, 16 bonds, bottom left) and corresponding change-in-contour-length histogram (bottom right). **C)** Change-in-contour-length histogram for the L-peptide, D-DNA (L,D-POC(2+3), 16 bonds] bound with a TFO conjugated with L-peptide (L,D-POC16, 44-45 bonds, top) and D-peptide (D,D-POC1, 16 bonds). N and n depict total numbers of features and distinct molecules, respectively.

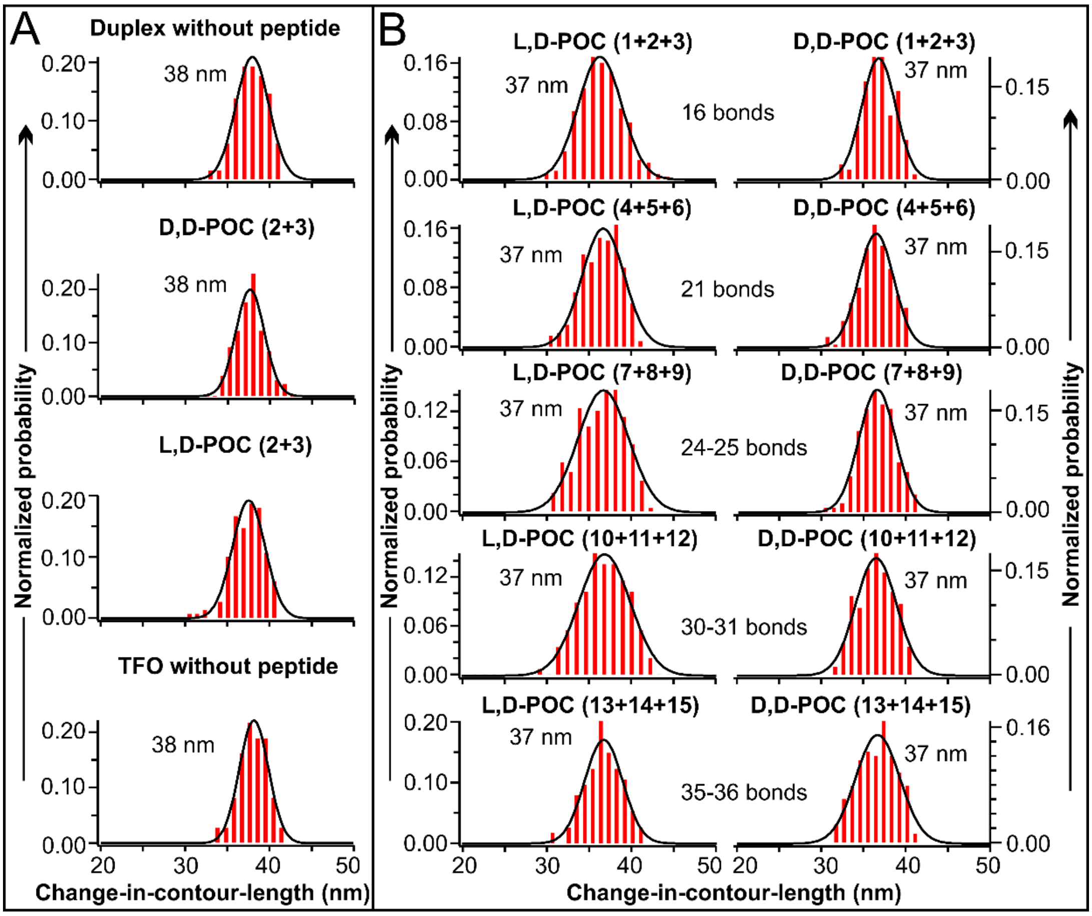

Supplementary Fig. 49. **A)** Change-in-contour-length histograms for duplex without peptide, D-peptide, D-DNA (D,D-POC(2+3)), L-peptide, D-DNA (L,D-POC(2+3)), and TFO without peptide, respectively (top to bottom). **B)** Change-in-contour-length histograms for the L-peptide, D-DNA (left) and D-peptide, D-DNA (right) with varying linker length (in number of bonds). See Figure 3 and Figure 4 in the main text for the number of features and distinct molecules.

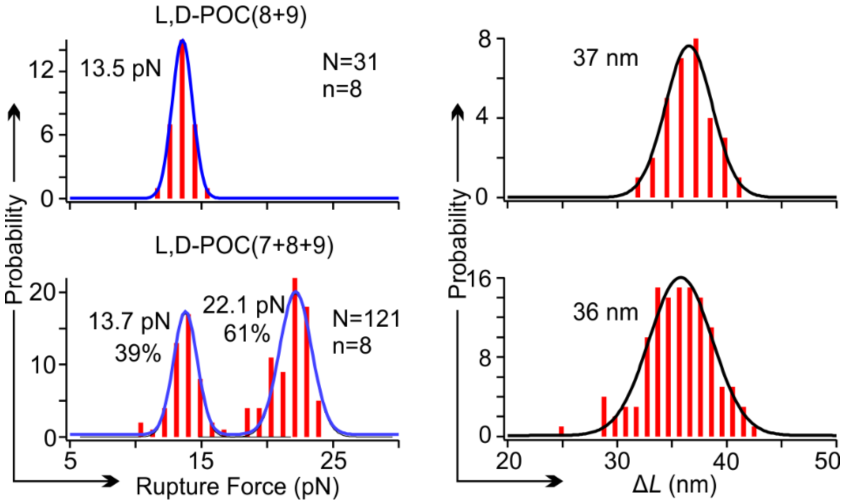

Supplementary Fig. 50 Rupture force and change-in-contour-length (ΔL) histograms for the **L,D-POC (8+9)** (top) and **L,D-POC(7+8+9)** (bottom) with 24-25 bonds linker length at 15-second incubation time. N and n depict total numbers of features and distinct molecules, respectively.

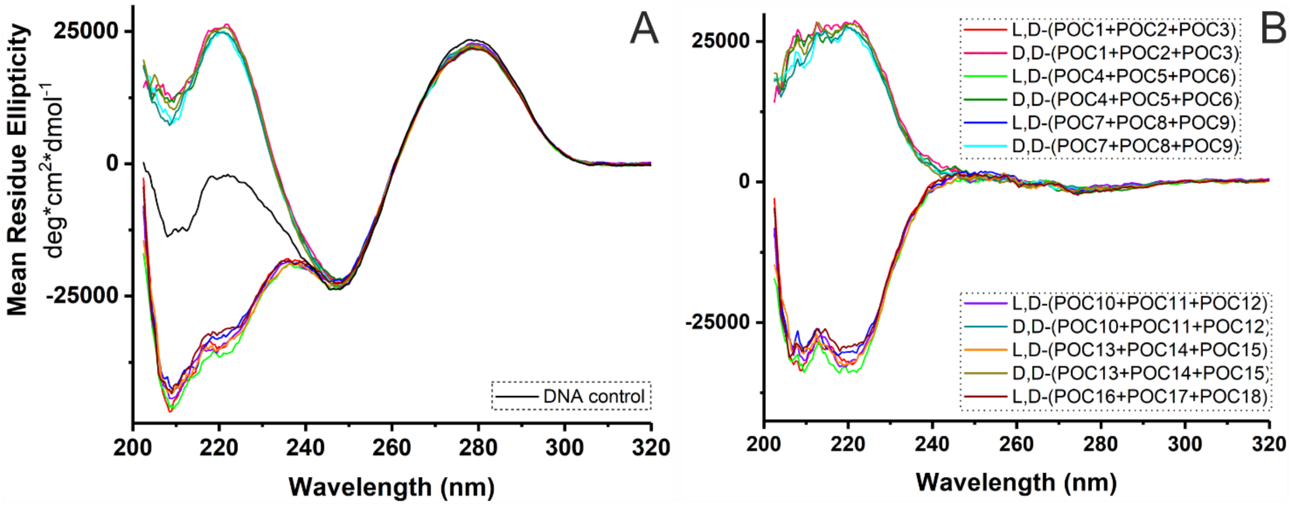

Supplementary Fig. 51. A) Circular dichroism spectra in 10 mM MES buffer (100 mM NaCl, pH 5.5) from 320 to 200 nm. The total peptide concentration was 9 µM for each POC triplex sample, determined by UV absorbance at 260 nm. B) Isolated peptide spectra of eleven POC triplex samples. The spectra of the DNA control have been subtracted from spectra of **POC**s. The signals beyond 202 nm were too noisy and removed (high HT values were also noticed).

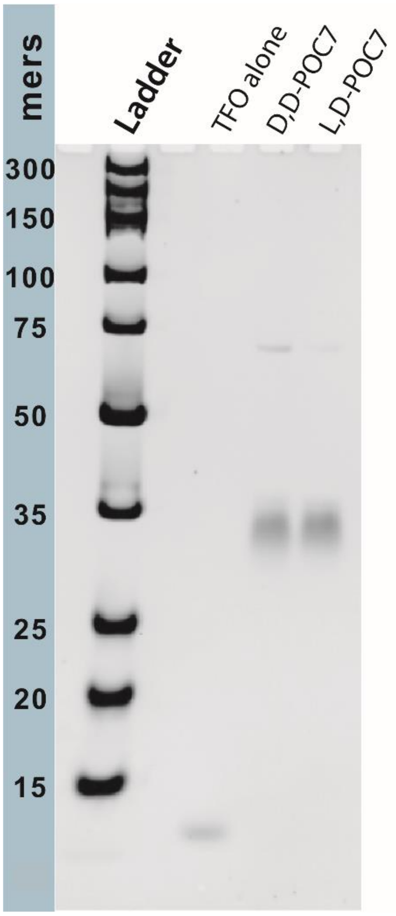

TFO alone: 3'-TTTTCCCTCTCTCT

Supplementary Fig. 52. 13% PAGE non-denaturing gel at pH 5.5 (100 mM MES buffer) under 4 °C. Lanes from left to right: **TFO alone**, **D,D-POC7** and **L,D-POC7**. All three samples were prepared at 15 µM before loading. The gel was visualized by UV excitation at 302 nm after SYBR Gold staining. The O'GeneRuler Ultra Low Range DNA Ladder from bottom to top: 15, 20, 25, 35, 50 75, 100, 150, 200 and 300-mers. The gel electrophoresis was repeated in triplicate, from which similar results were obtained accordingly.

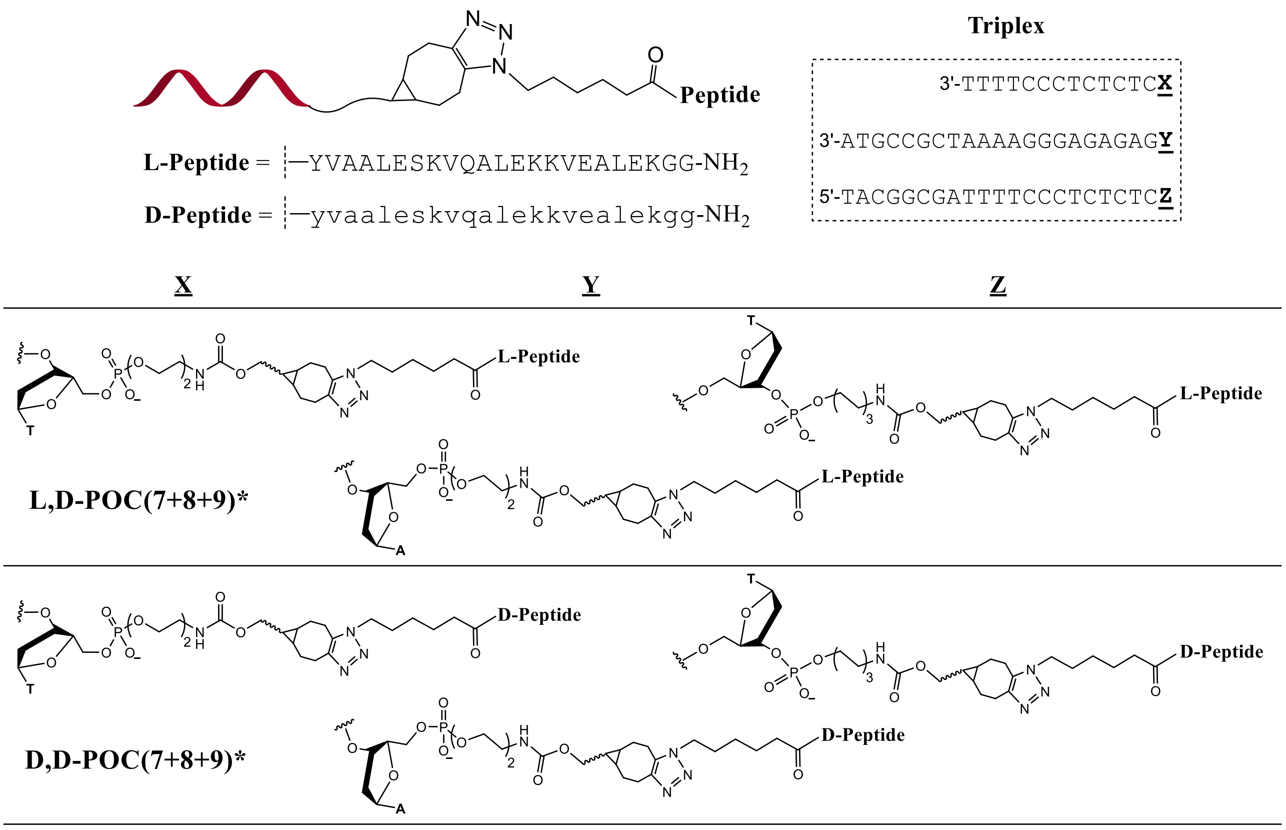

Supplementary Fig. 53. L,D-POC(7+8+9)*[7] and **D,D-POC(7+8+9)*** used for SAXS analysis and MD simulation. Y, V, L, E, S, K, Q, A, G are L-amino acids whereas y, v, l, e, s, k, q, a, g are D-amino acids. A, G, C and T are natural DNA monomers.

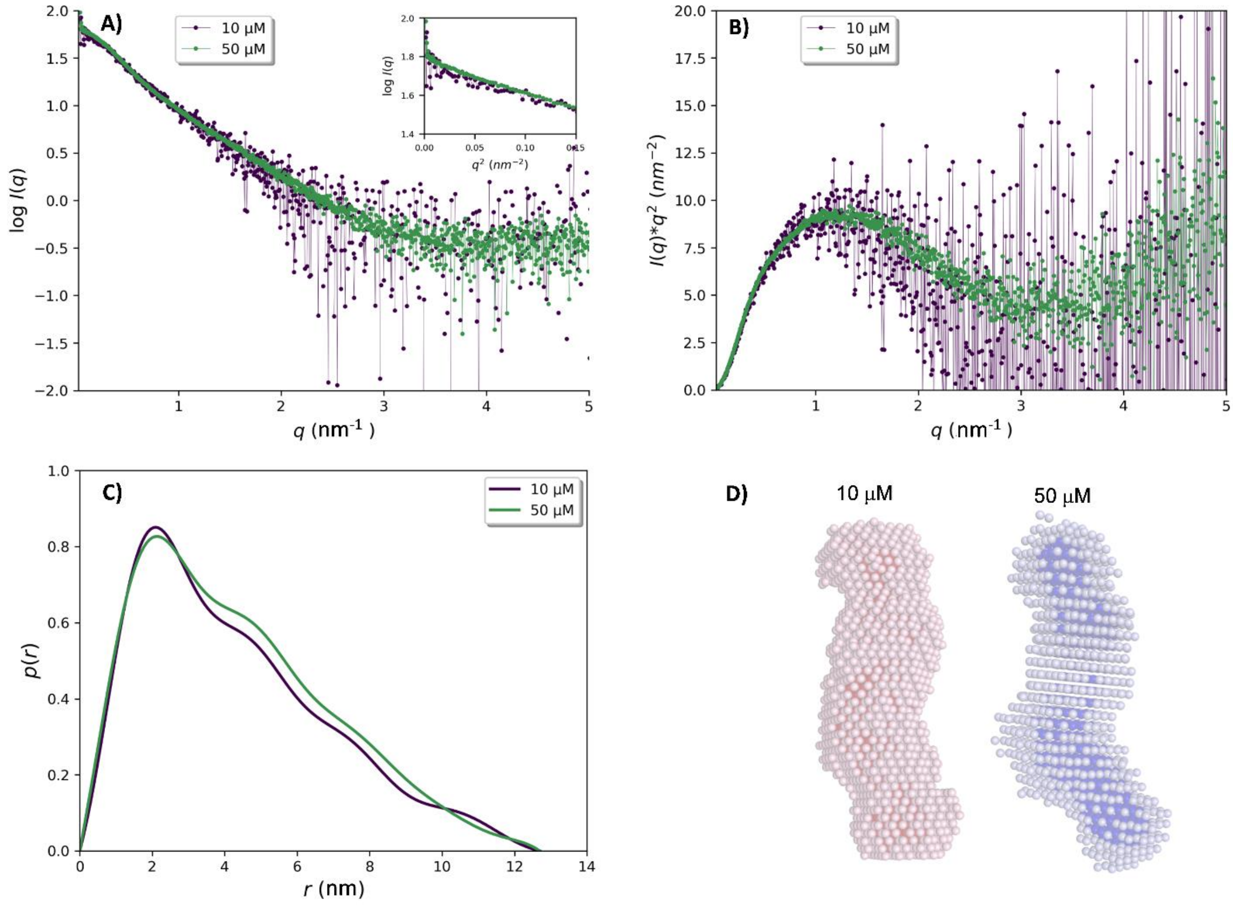

Supplementary Fig. 54. SAXS analyses for **D,D-POC(7+8+9)***at 10 µM and 50 µM. (A) SAXS intensity curves with inset Guinier plots showing no significant changes in intermolecular interactions at the two concentrations. (B) Kratky plots indicating folded yet flexible complexes. (C) Pair distance distribution functions. (D) *Ab initio* models with concentration-dependent conformational differences. The SAXS data were normalized using the 50 µM sample.

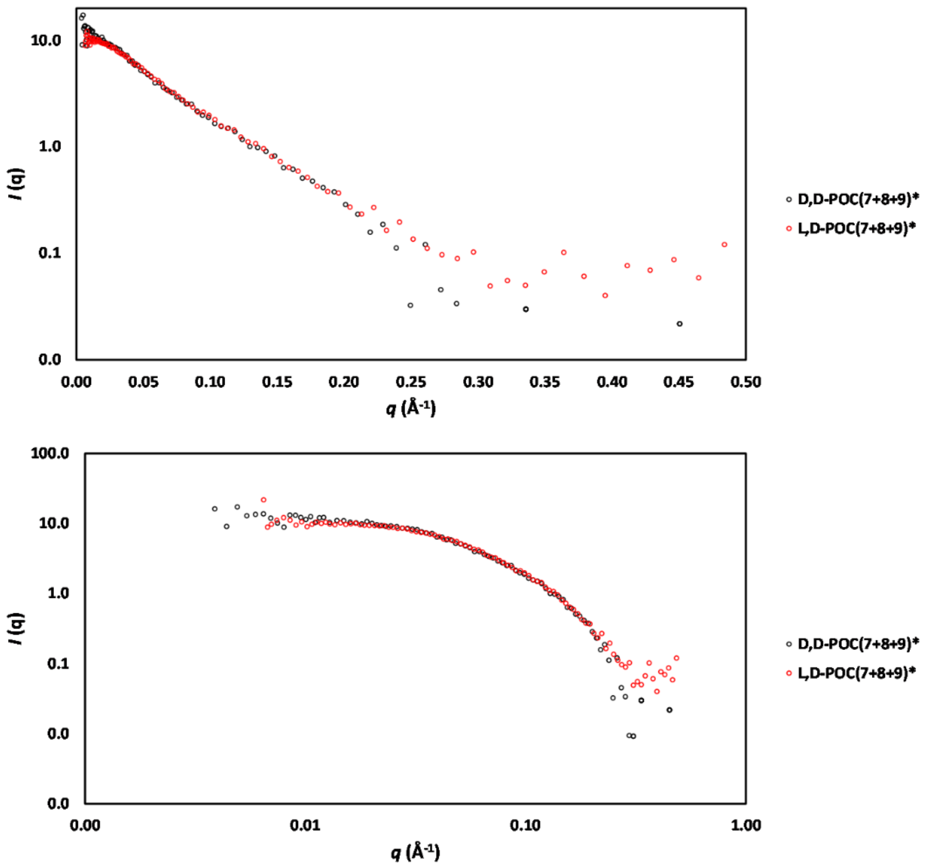

Supplementary Fig. 55. Demonstration of the similarity of SAXS intensity curves for **D,D-POC(7+8+9)*** and its diastereoisomer **L,D-POC(7+8+9)*** from our previous study[7]. For direct comparison, both macromolecular diastereoisomers were prepared at 10 µM in the same buffer condition (10 mM acetate buffer, 100 mM NaCl, pH 5.5). Please note that these profiles were obtained at different beamlines. The latter profile was not calibrated to absolute intensity, and the profile has been superimposed on the profile for **D,D-POC(7+8+9)*.** Top: log-linear plot; Bottom: log-log plot.

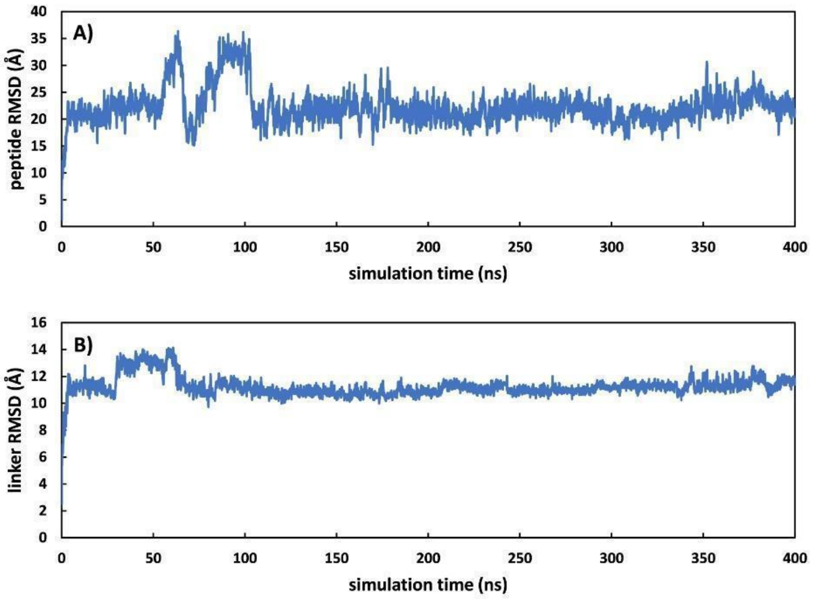

Supplementary Fig. 56. RMSD for the MD simulation of **D,D-POC(7+8+9)***. (A) RMSD for peptide atoms. (B) RMSD for linker atoms. We used RMSD as a measure of movement of peptide and linker atoms relative to the positionally restrained DNA triplex, and hence did not carry out superposition prior to RMSD evaluation.

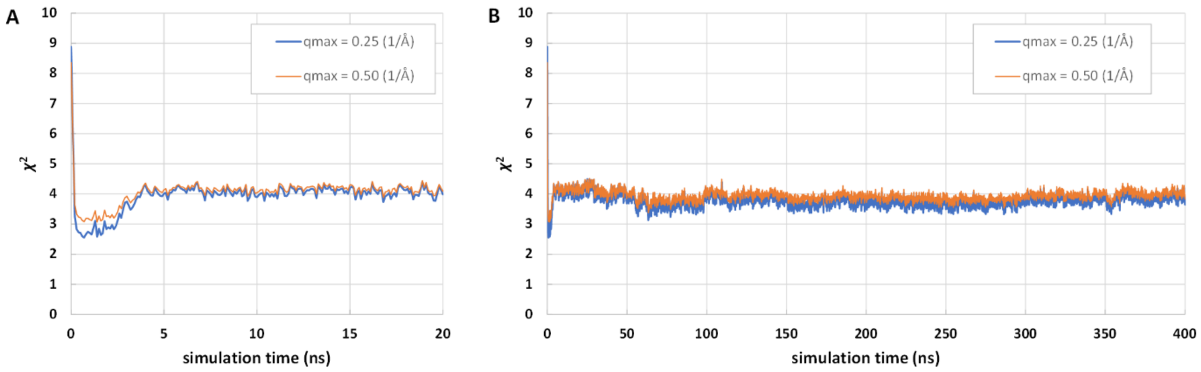

Supplementary Fig. 57. Goodness of fit for SAXS predictions from **D,D-POC(7+8+9)*** trimeric structures extracted at different time points in the MD trajectory. (A) First 20 ns. (B) Entire 400 ns trajectory. Blue line: Fit using maximum q of 0.25 Å^{-1}. Orange line: Fit using maximum q of 0.50 Å^{-1}

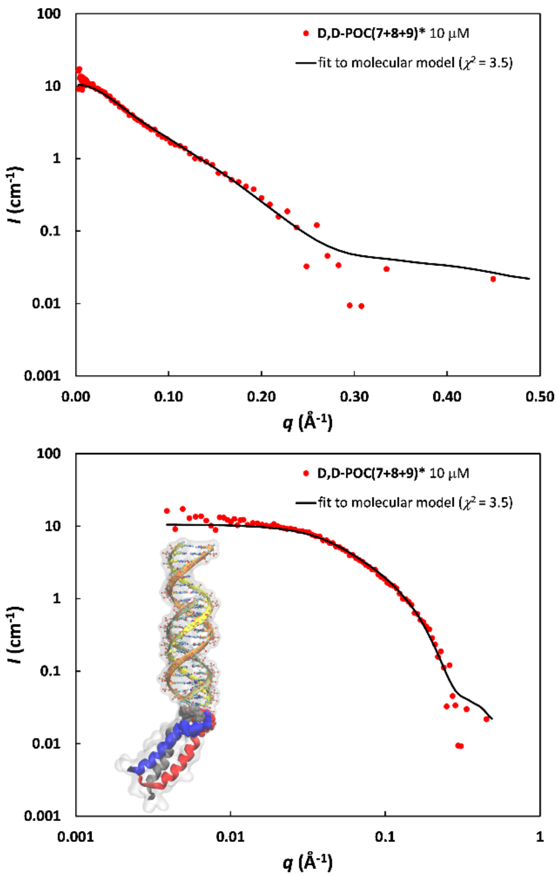

Supplementary Fig. 58 SAXS data for **D,D-POC(7+8+9)*** at 10 µM (red) and fit (black) to MD structure (inset). Peptide chains are shown as blue, red, and grey ribbons. Oligonucleotide chains are shown in orange, yellow and ochre. Linkers are shown as van der Waals spheres in the same colours as the peptide chains to which they are attached. The van der Waals surface of the POC assembly is shown in transparent white. Top: Log-linear plot. Bottom: Log-log plot.

Appendix

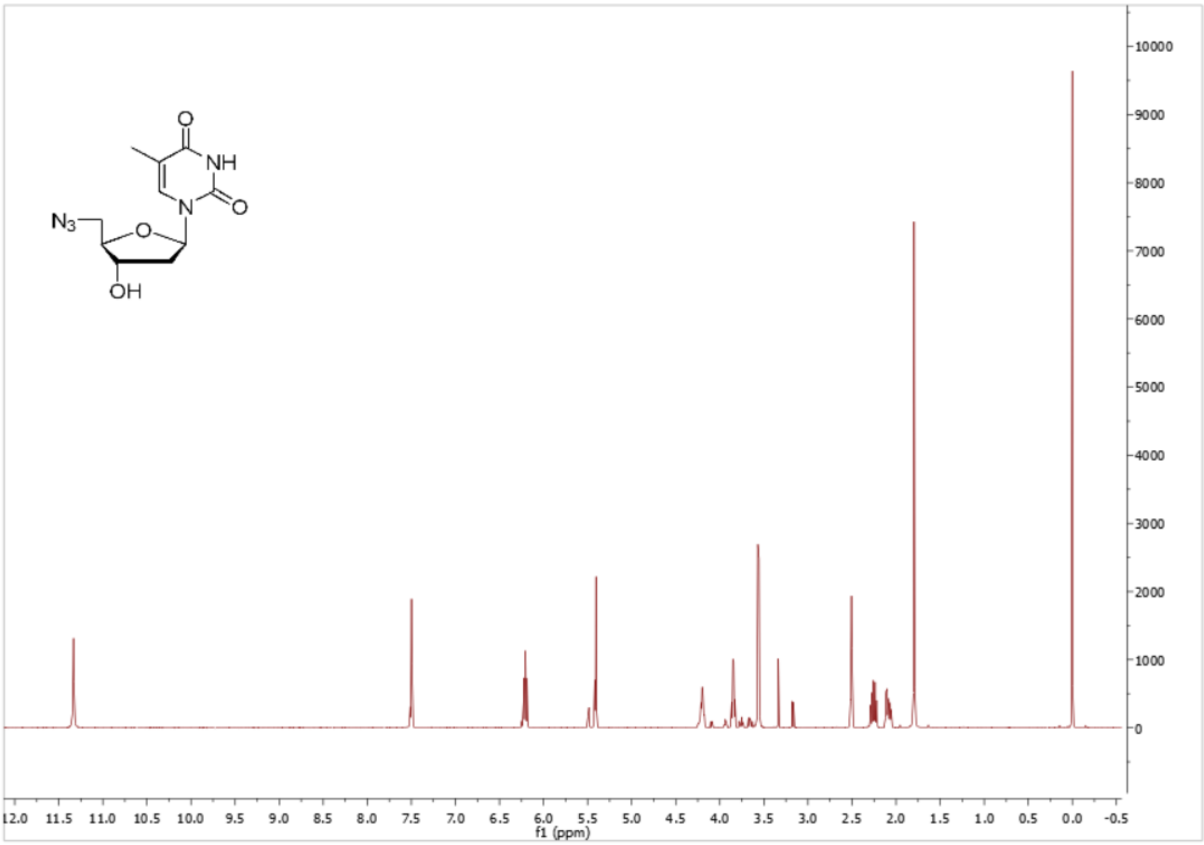

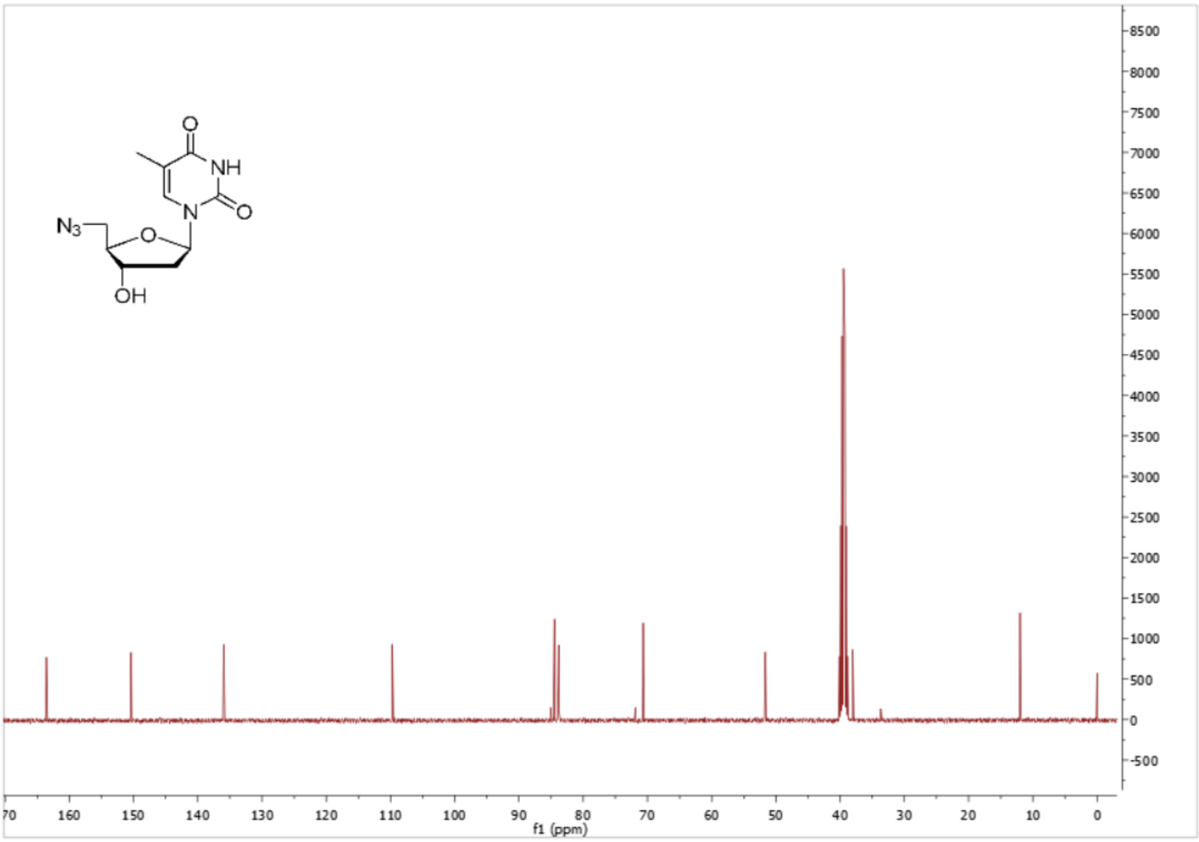

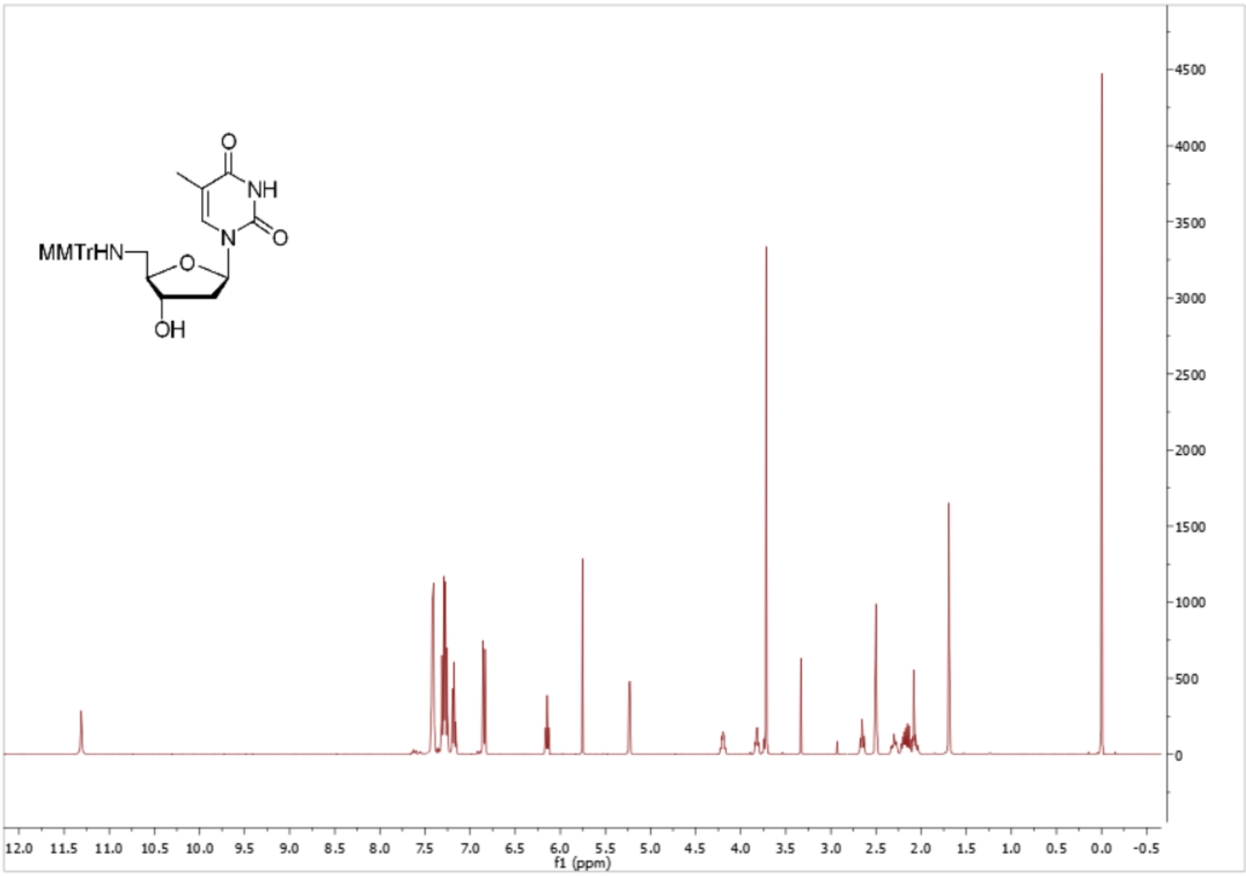
O
NH
O
N
MMTrHN
O
OH
f1 (ppm)

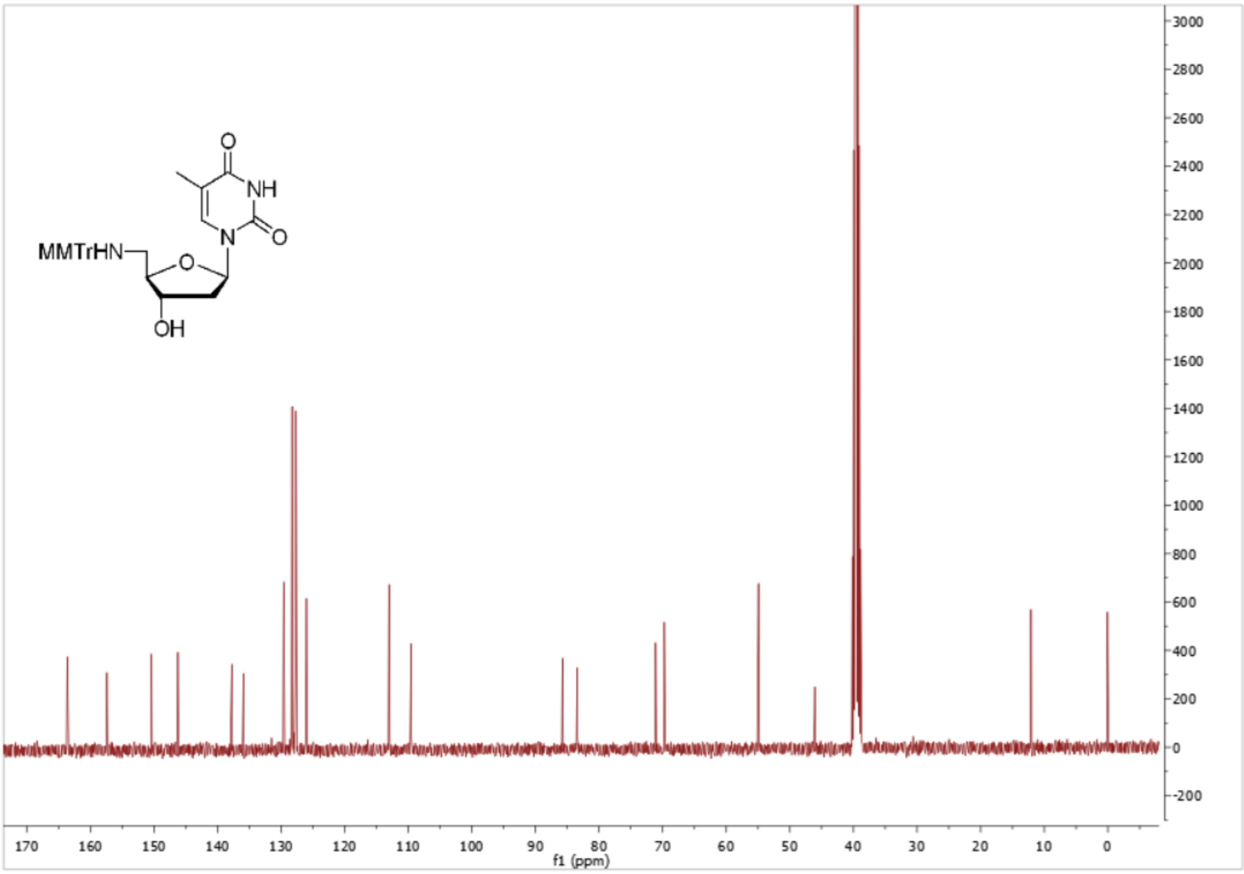
O
NH
O
N
MMTrHN
O
OH
f1 (ppm)

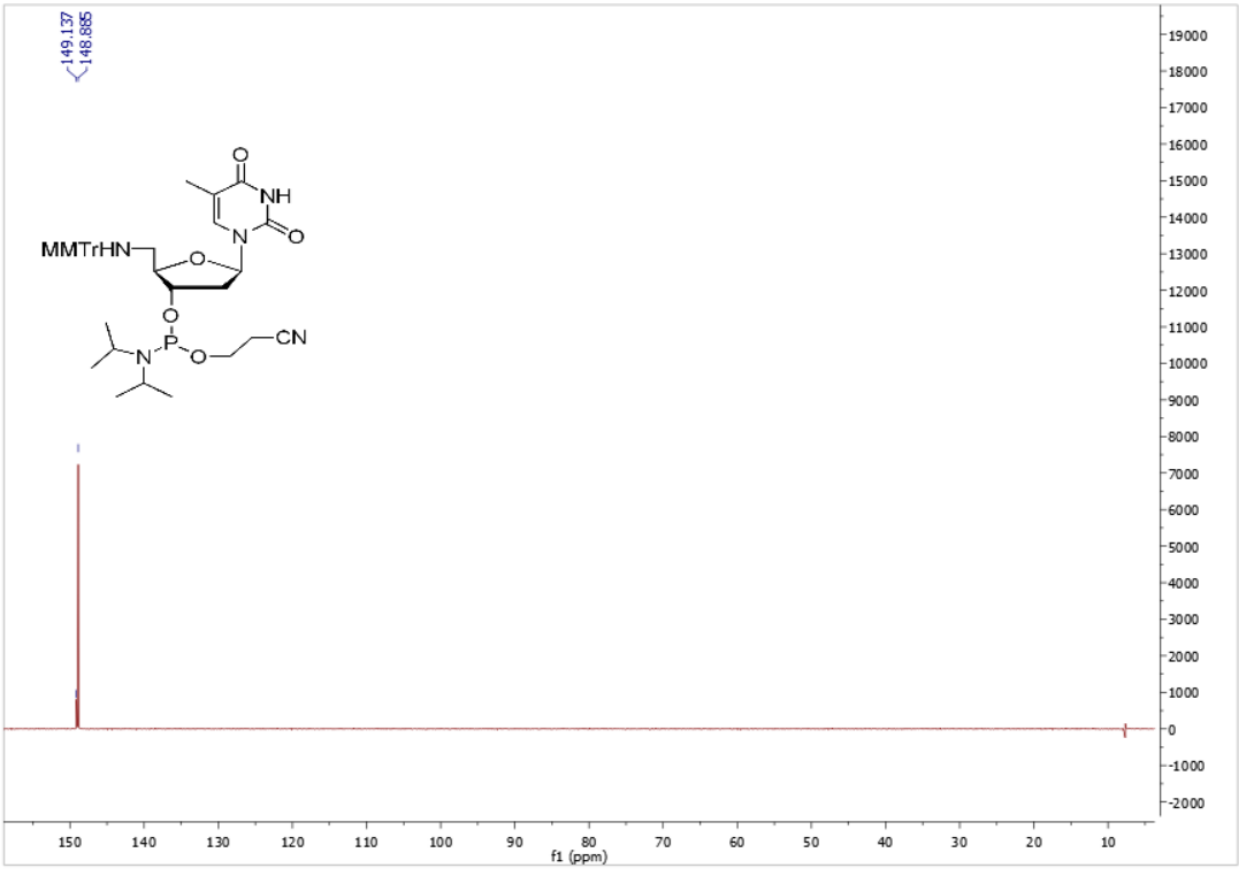
149.137
148.885
MMTrHN
NH
CN
f1 (ppm)

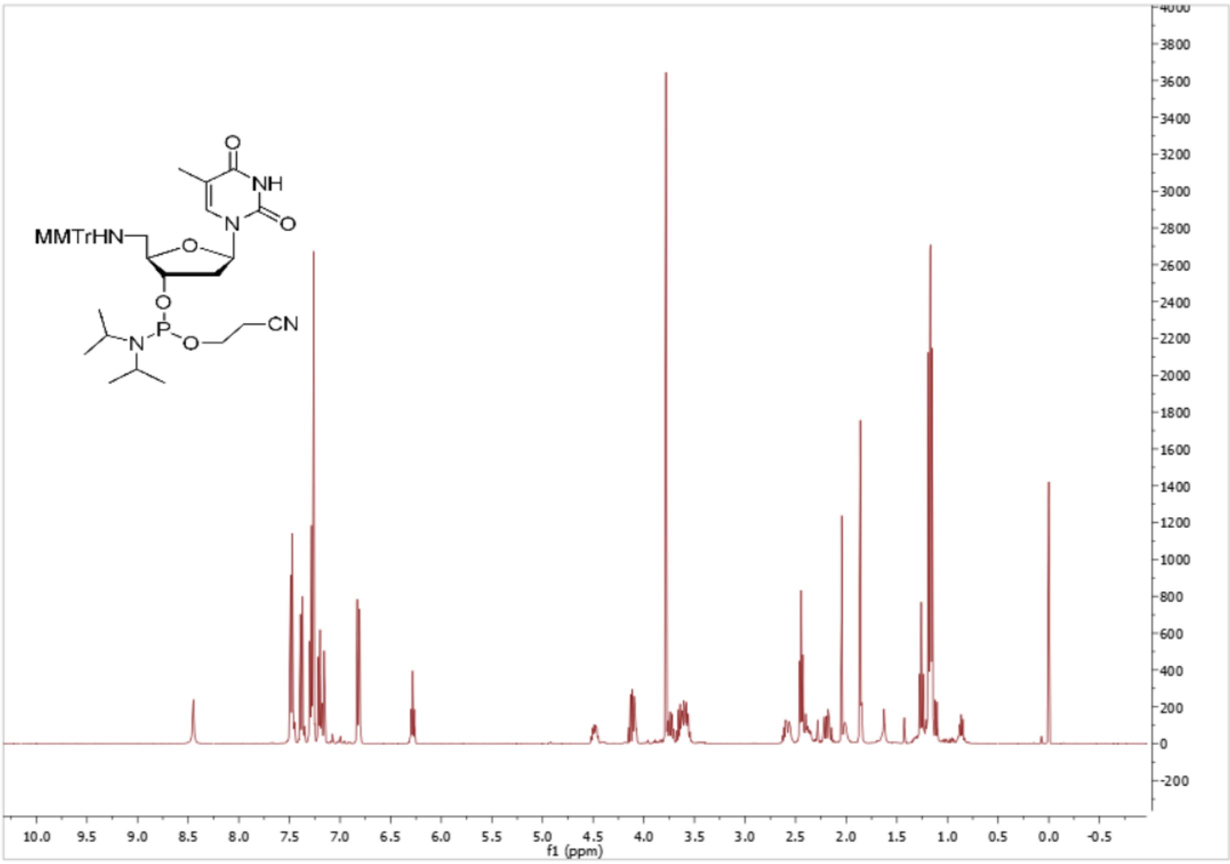
MMTrHN
NH
CN
f1 (ppm)

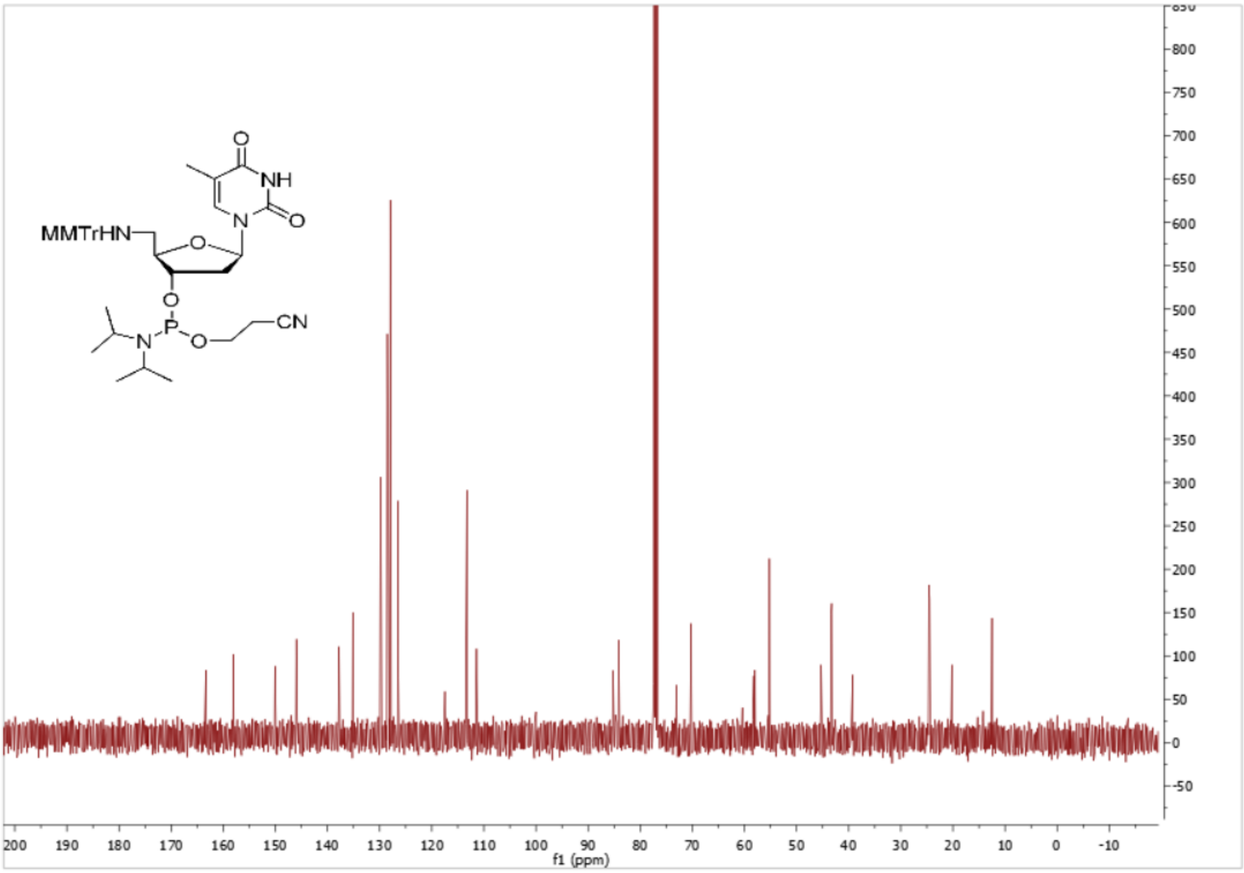
MMTrHN
O
NH
N
O
O
O
P
N
O
CN
f1 (ppm)

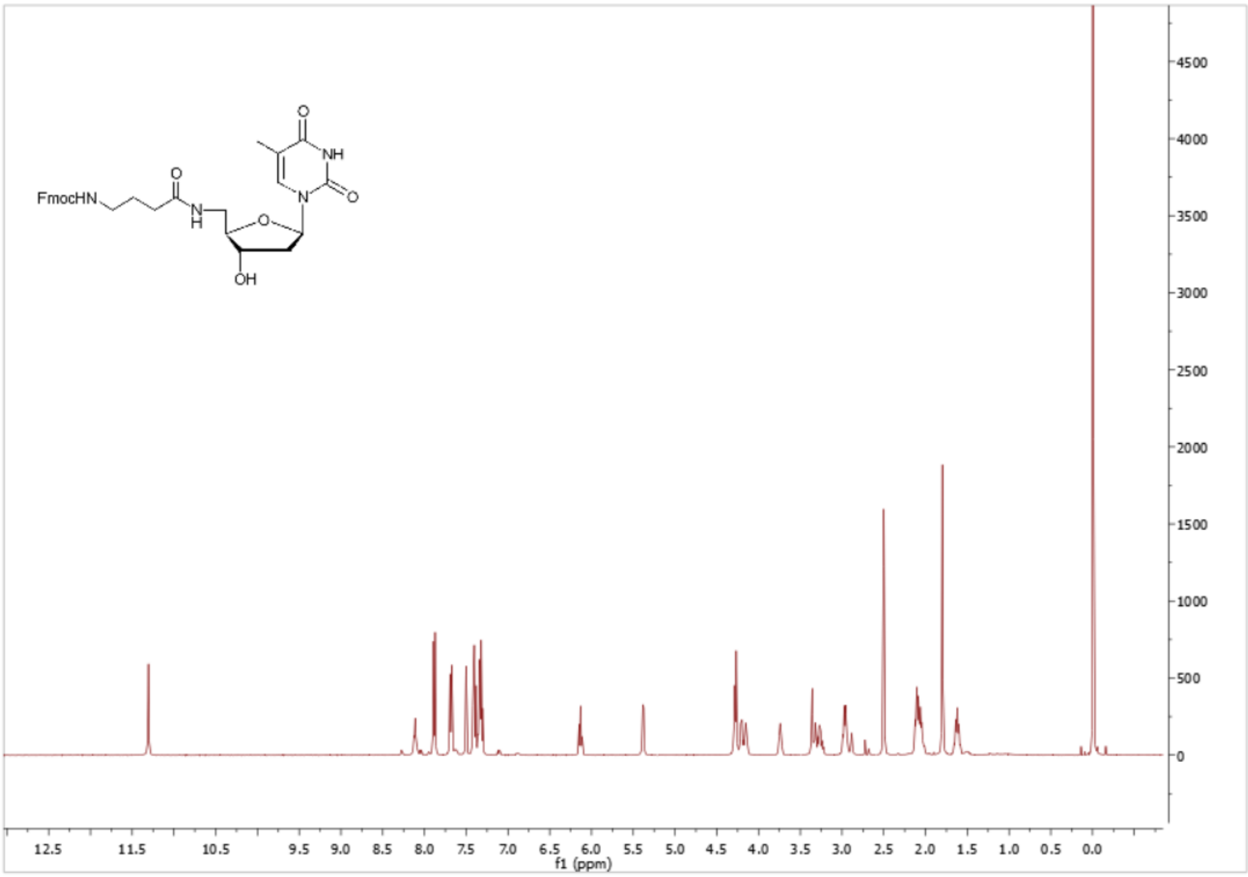
FmocHN
O
N
H
O
NH
N
O
O
OH
f1 (ppm)

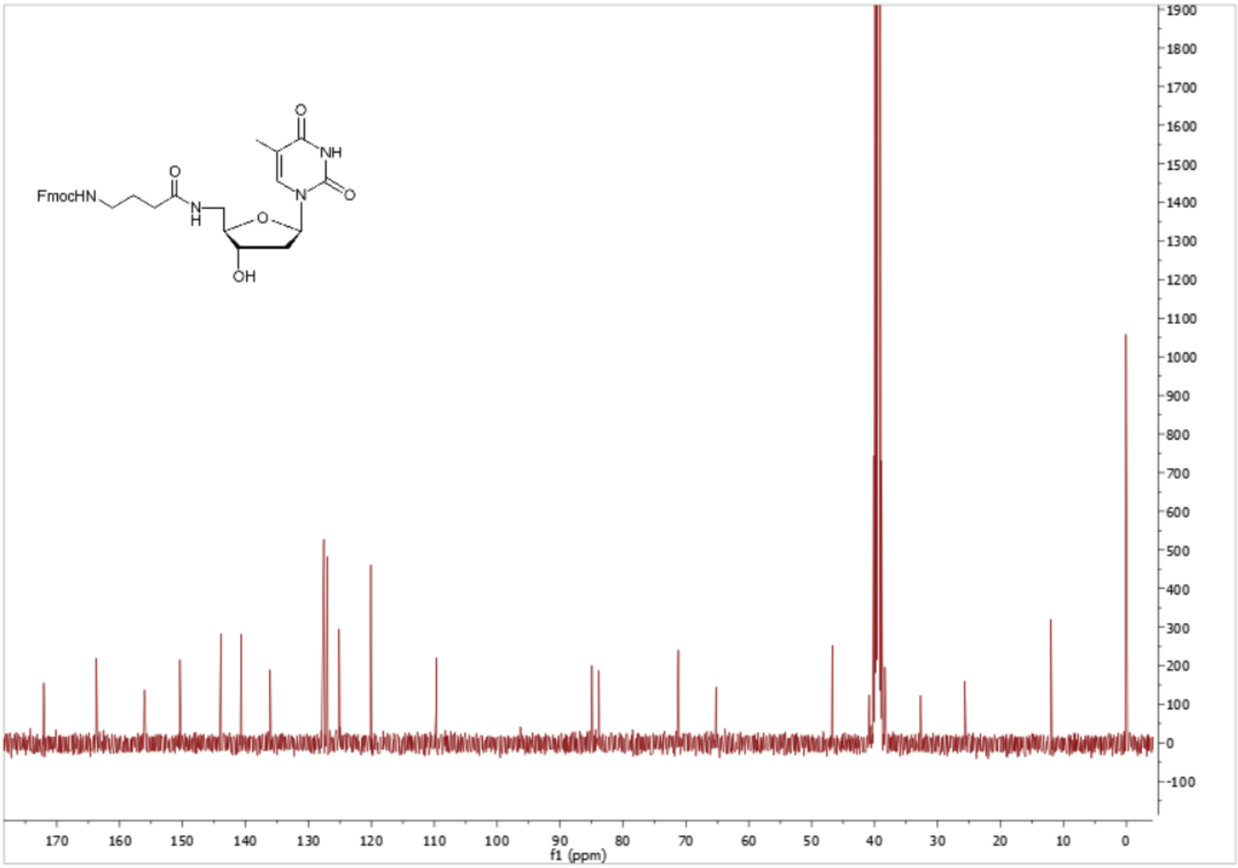
FmocHN
NH
OH
f1 (ppm)

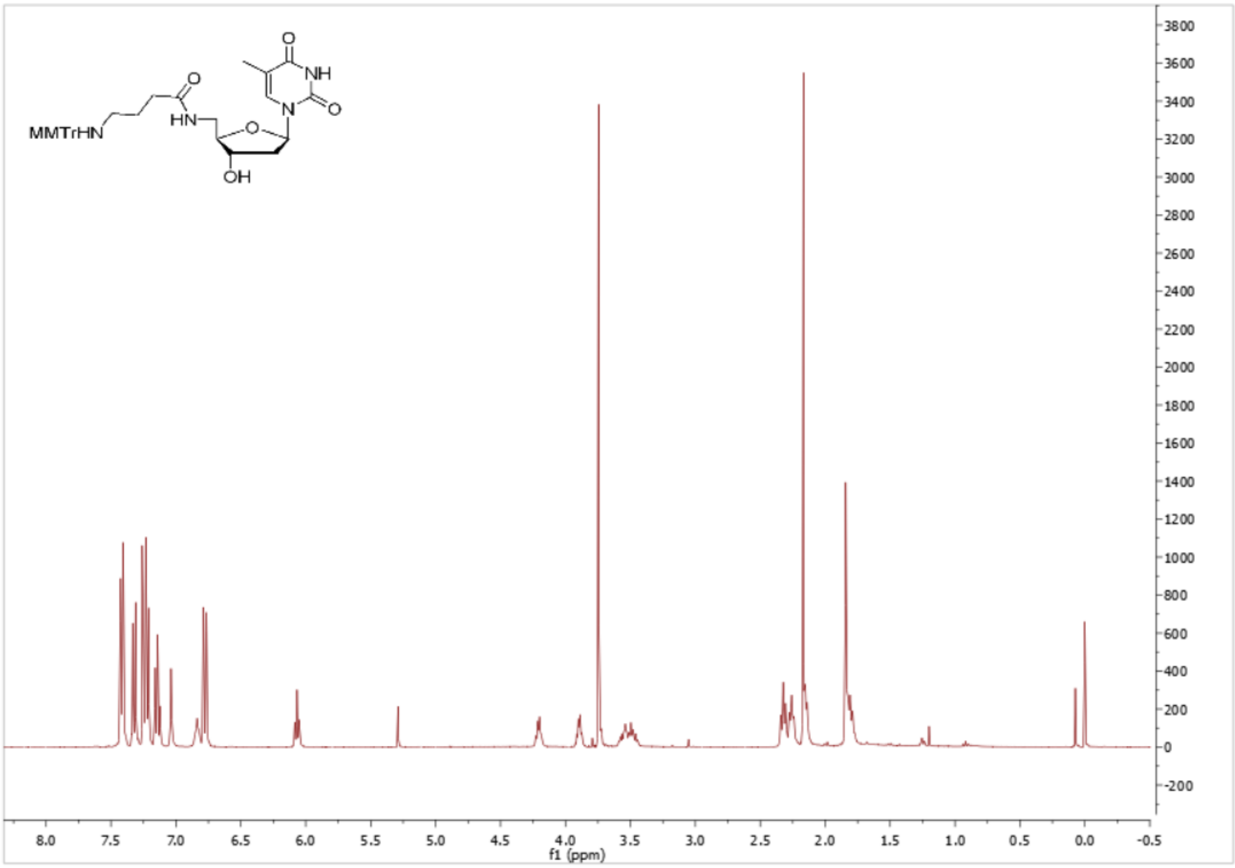
MMTrHN
HN
NH
OH
f1 (ppm)

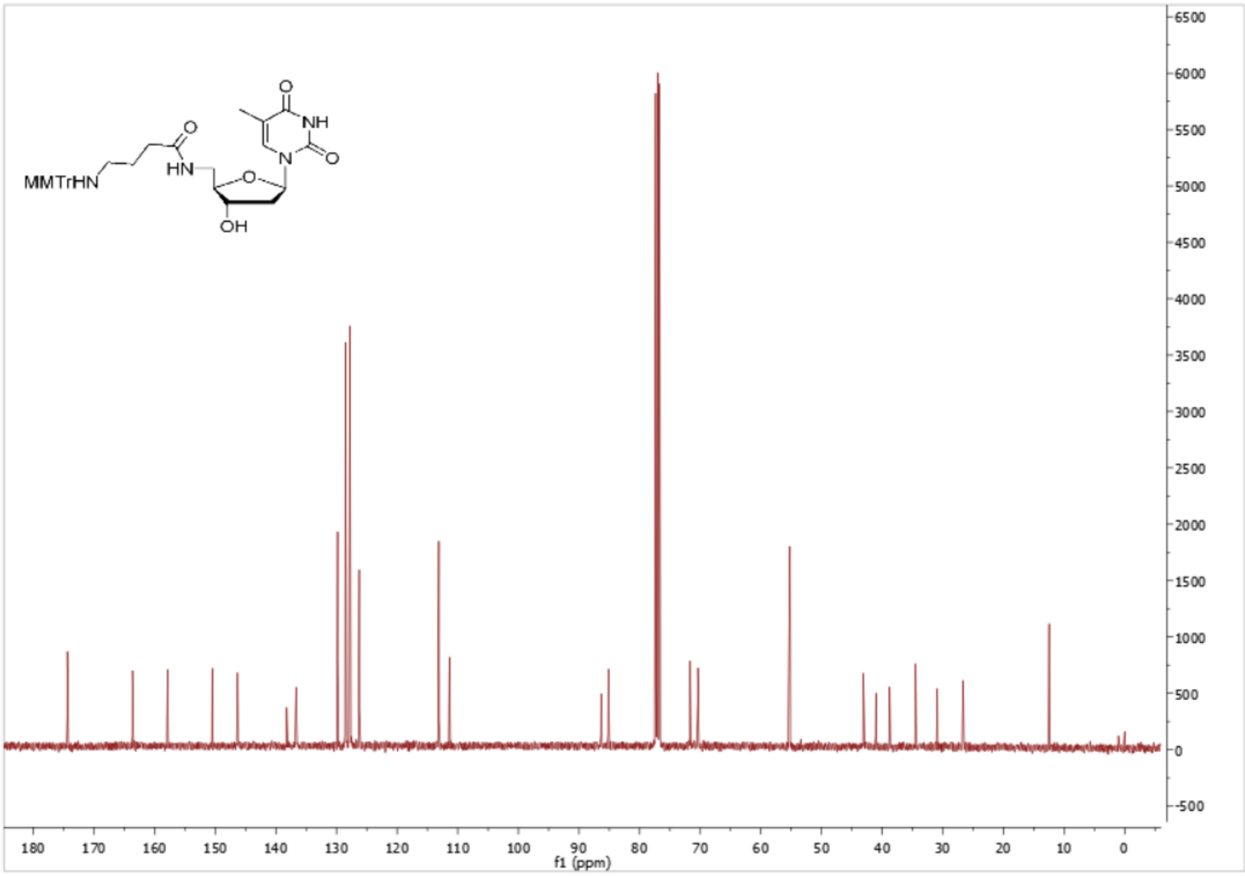
MMTrHN
HN
O
NH
N
O
OH
6500
6000
5500
5000
4500
4000
3500
3000
2500
2000
1500
1000
500
0
-500
180
170
160
150
140
130
120
110
100
90
80
70
60
50
40
30
20
10
0
f1 (ppm)

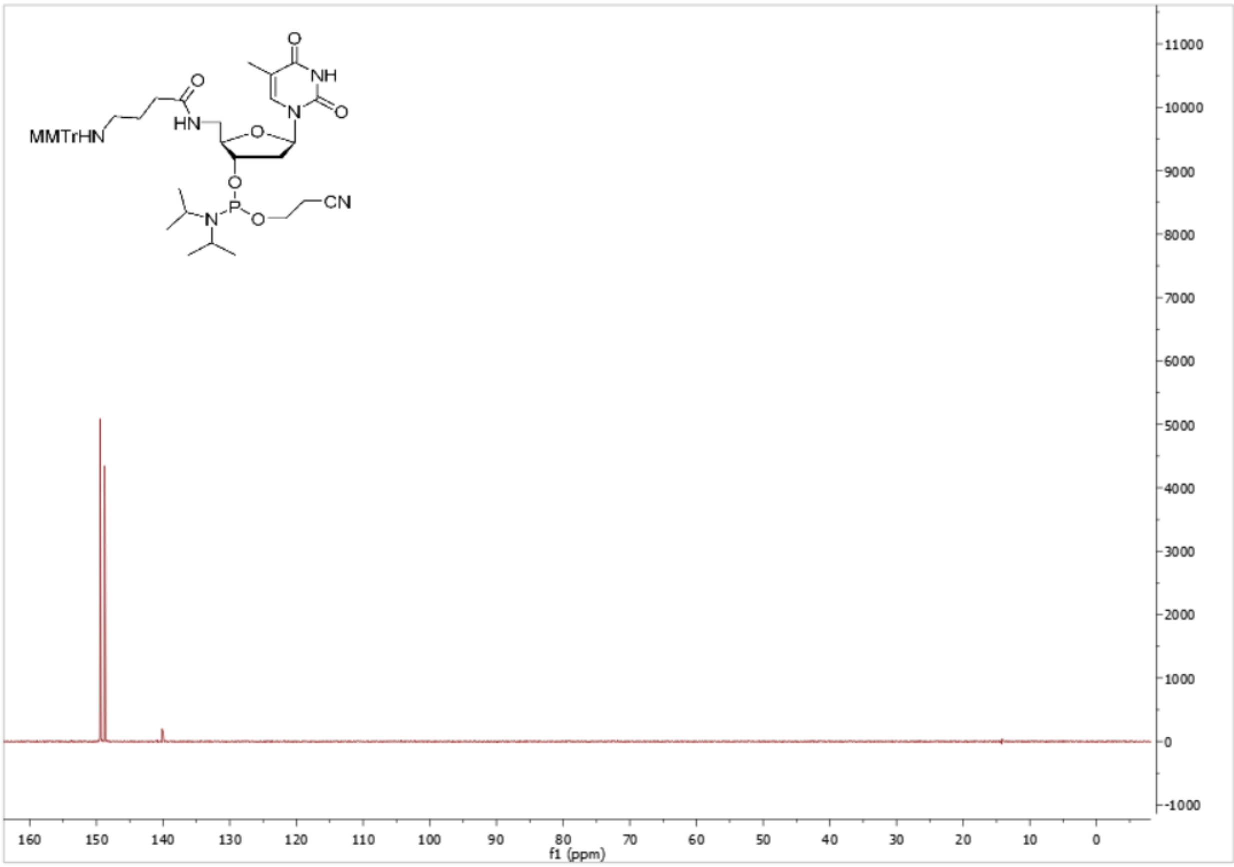
MMTrHN
HN
O
NH
N
O
P
N
CN
11000
10000
9000
8000
7000
6000
5000
4000
3000
2000
1000
0
-1000
160
150
140
130
120
110
100
90
80
70
60
50
40
30
20
10
0
f1 (ppm)

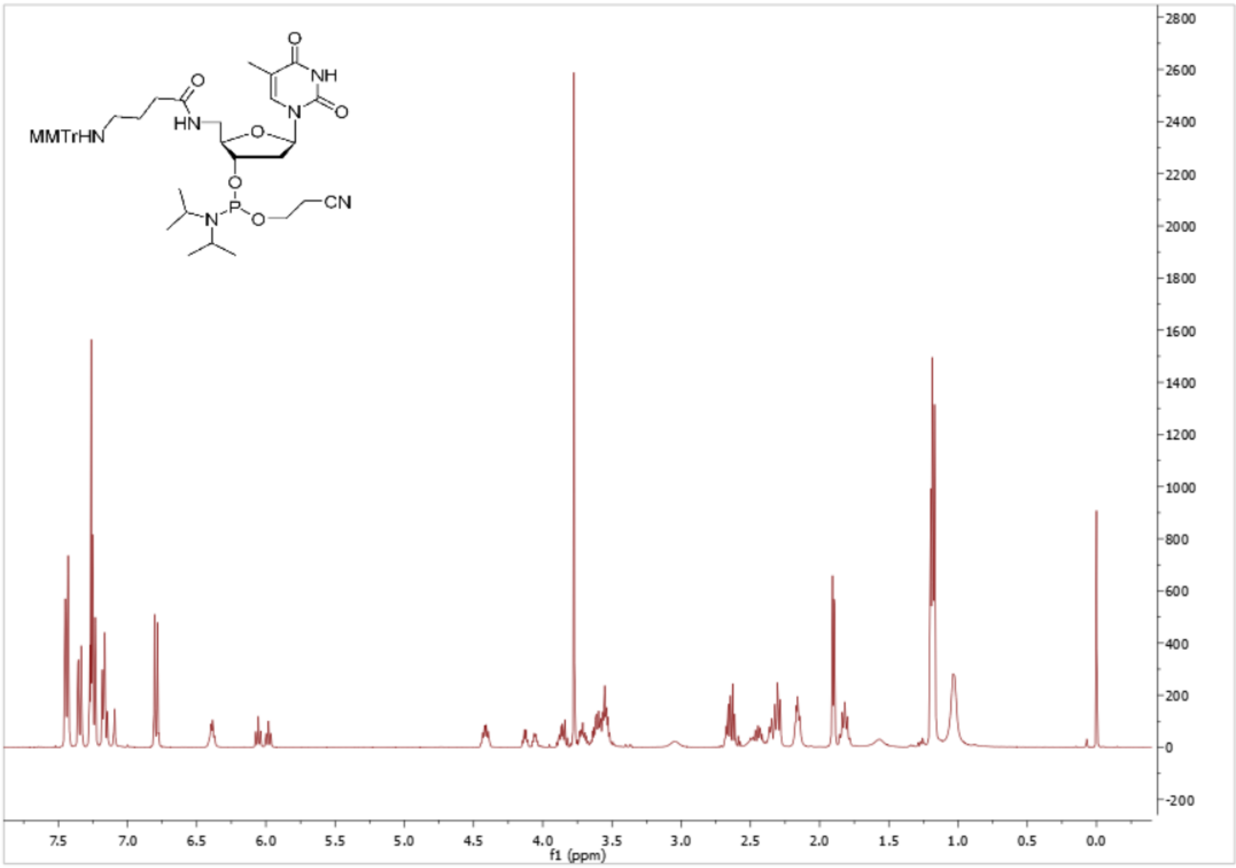

MMTrHN
HN
NH
CN
f1 (ppm)

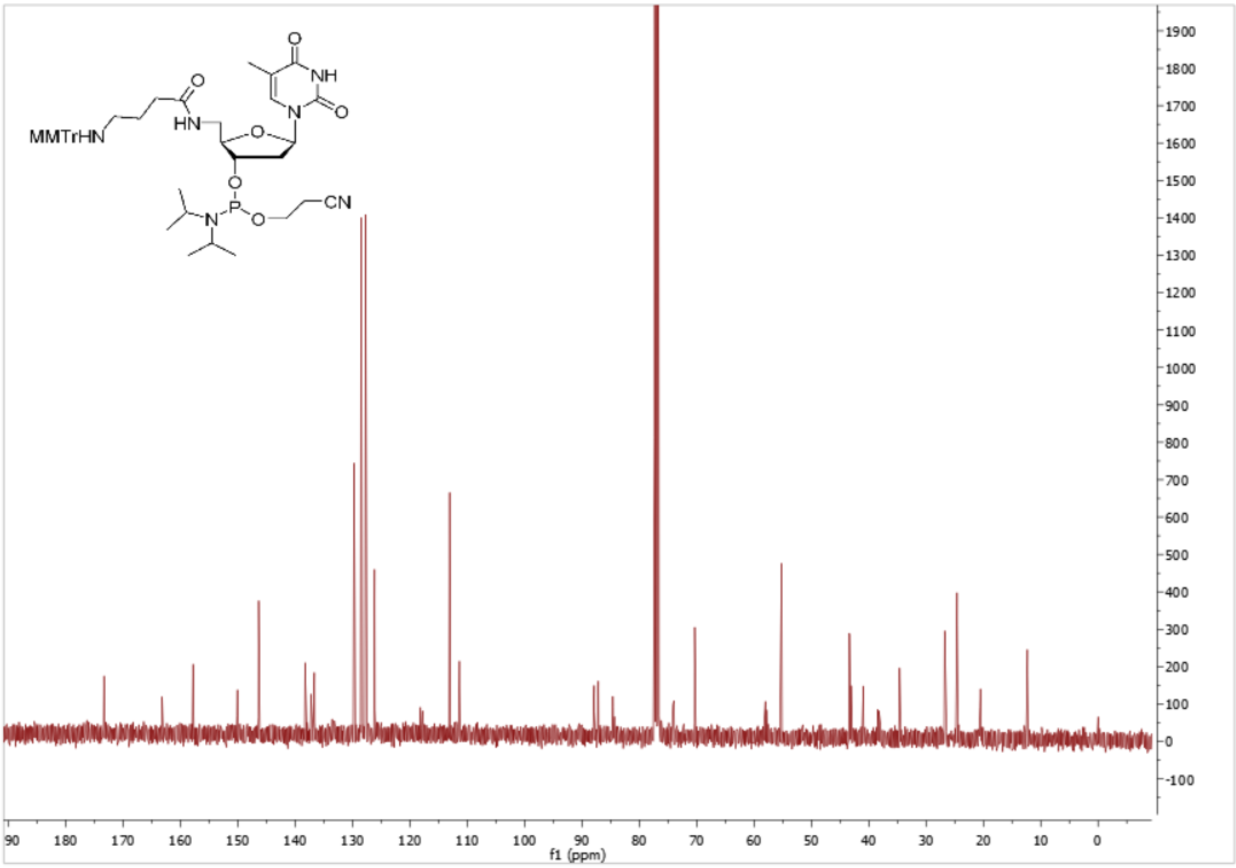

MMTrHN
HN
NH
CN
f1 (ppm)

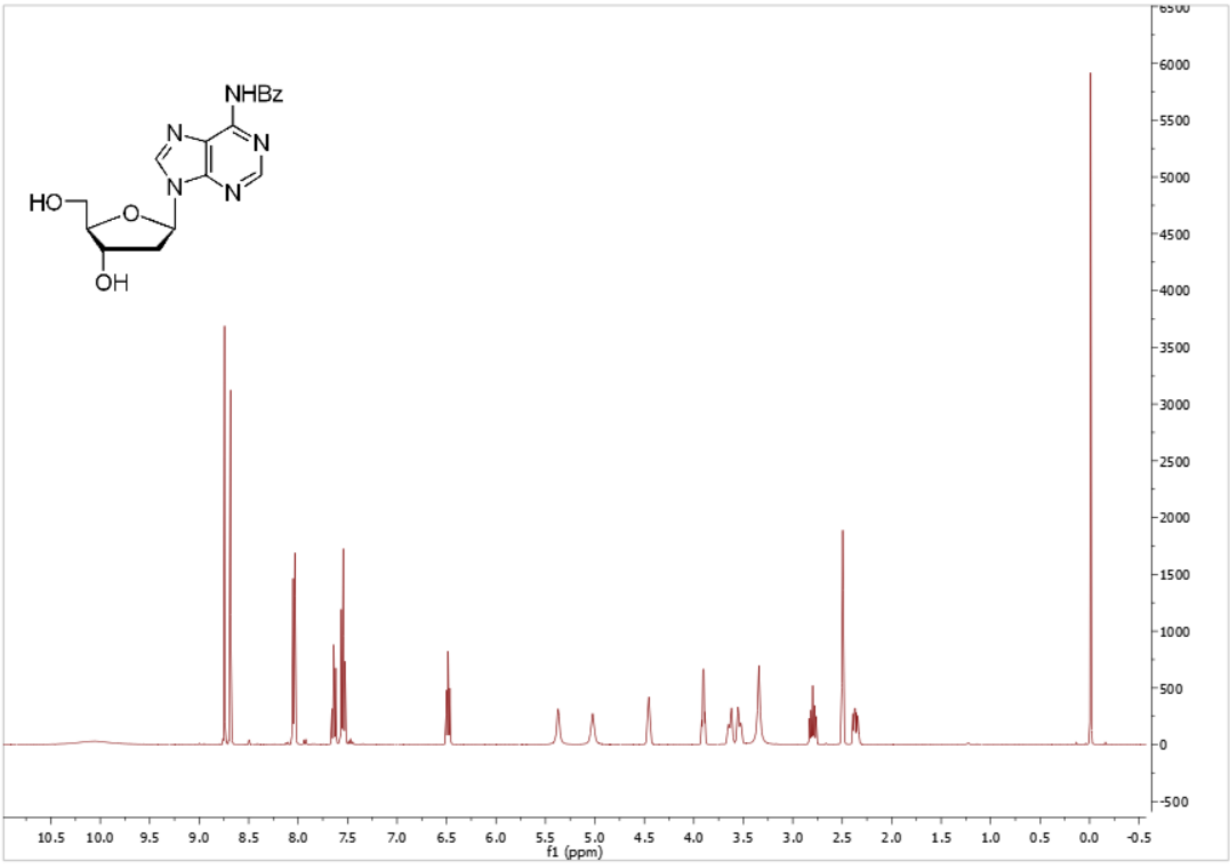
NHBz
N
N
N
N
HO
O
OH
f1 (ppm)

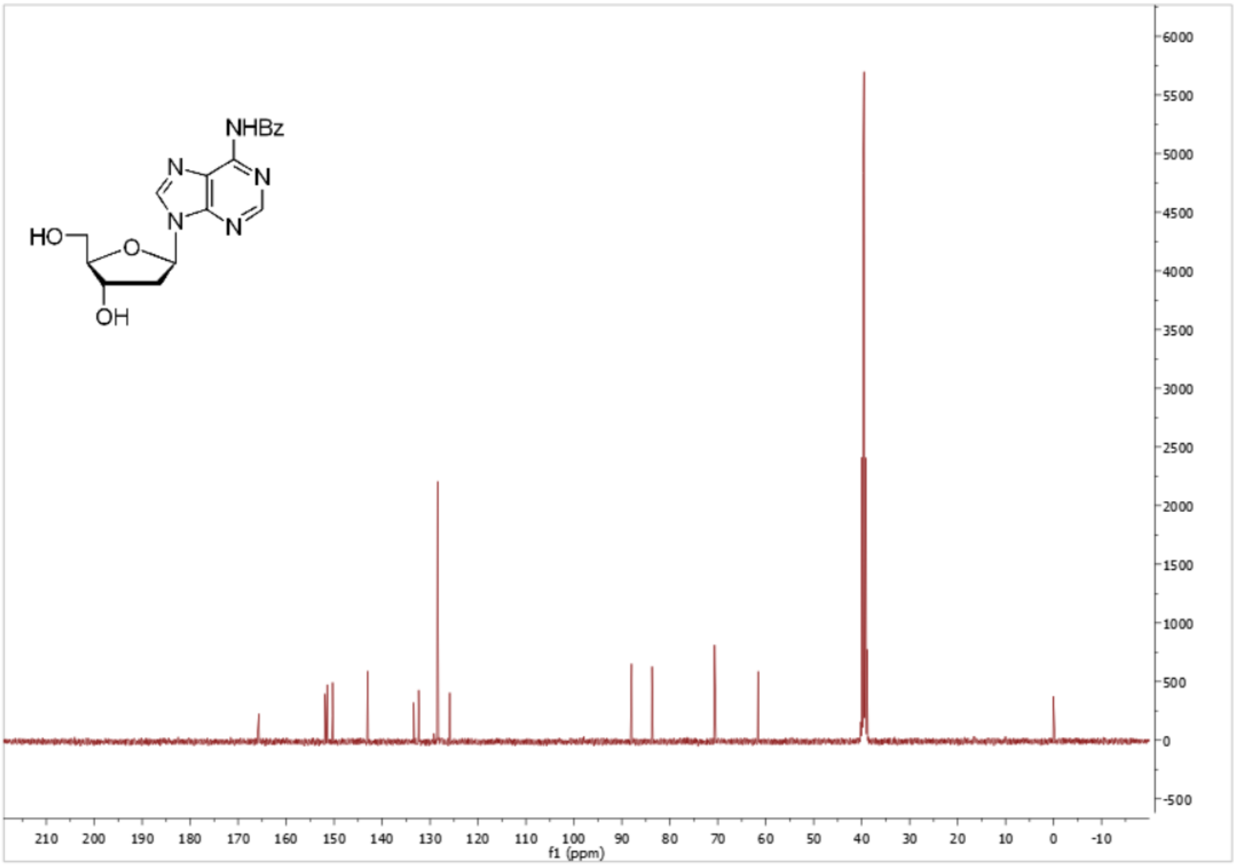
NHBz
N
N
N
N
HO
O
OH
f1 (ppm)

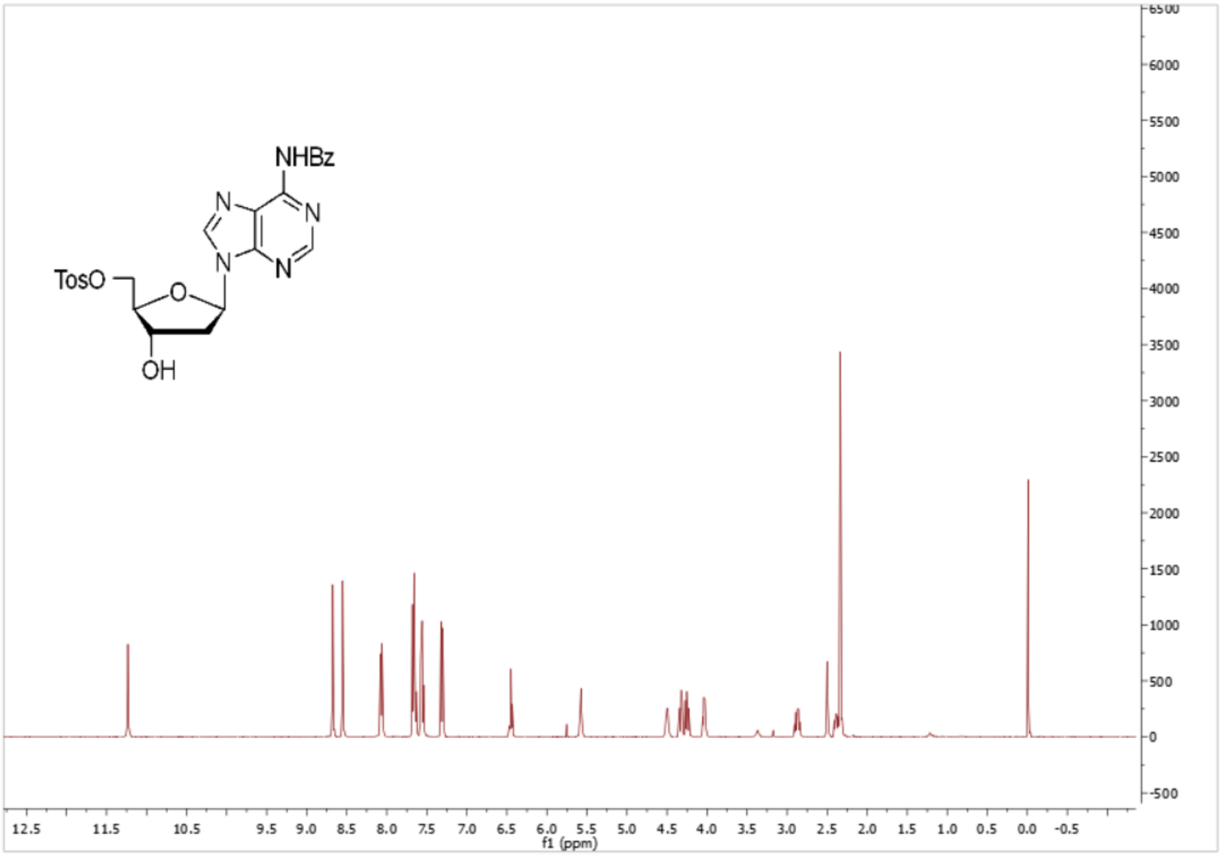
NHBz
N
N
N
N
TosO
O
OH
f1 (ppm)

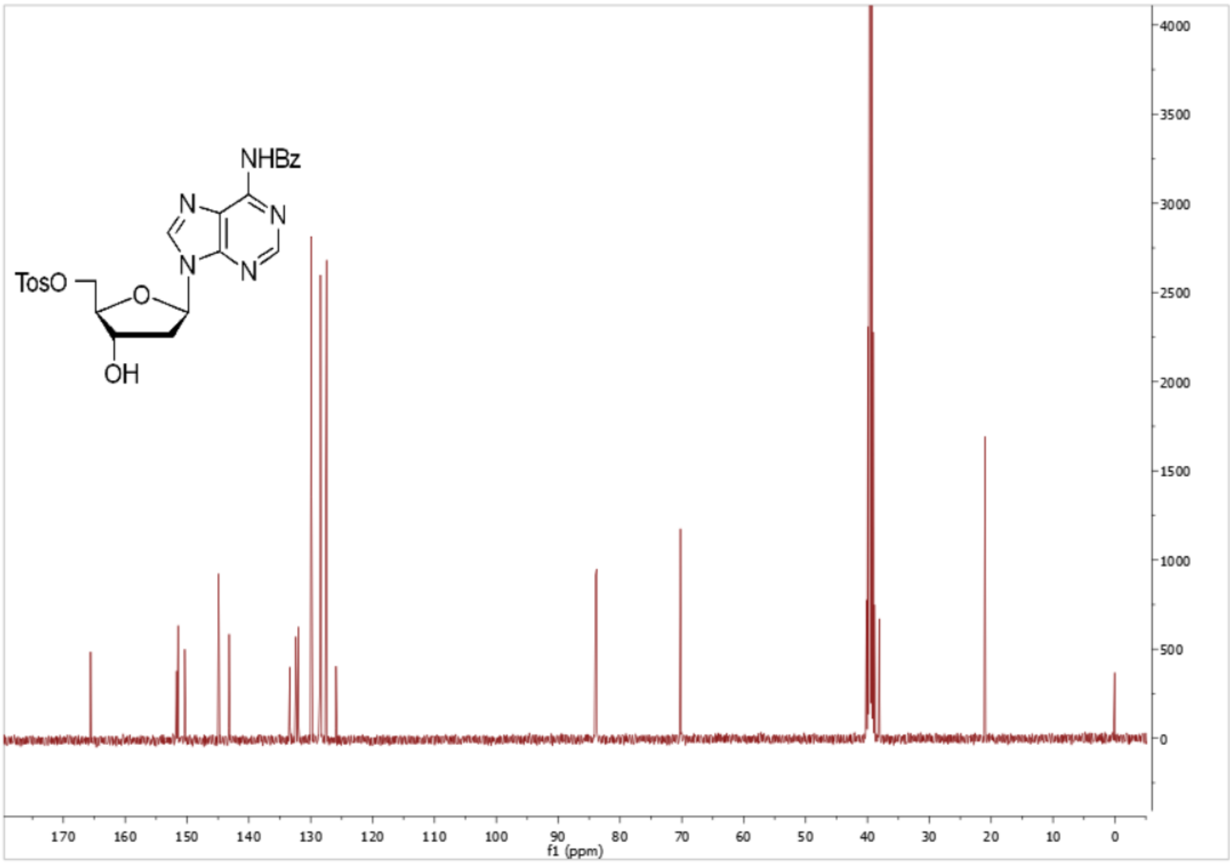
NHBz
N
N
N
N
TosO
O
OH
f1 (ppm)

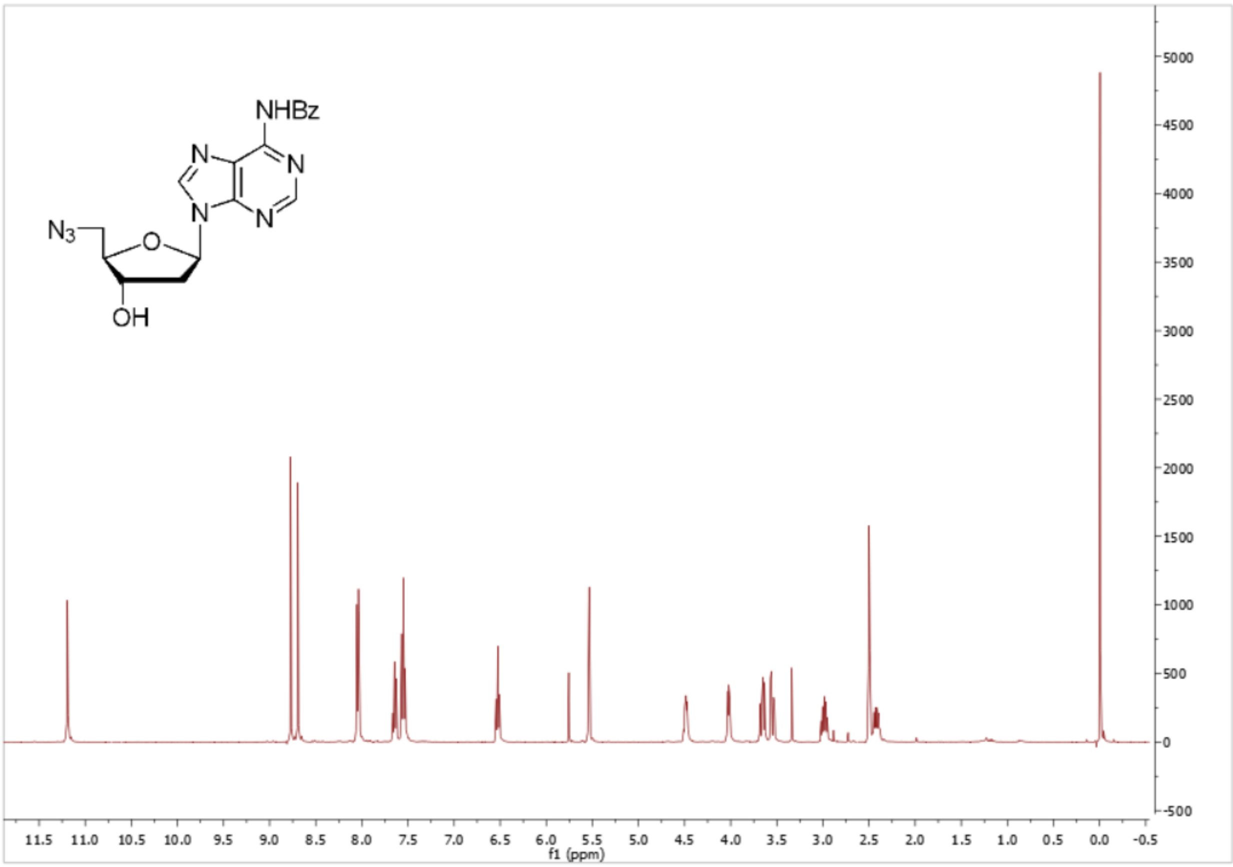
NHBz
N
N
N
N
N3
O
OH
5000
4500
4000
3500
3000
2500
2000
1500
1000
500
0
-500
11.5 11.0 10.5 10.0 9.5 9.0 8.5 8.0 7.5 7.0 6.5 6.0 5.5 5.0 4.5 4.0 3.5 3.0 2.5 2.0 1.5 1.0 0.5 0.0 -0.5
f1 (ppm)

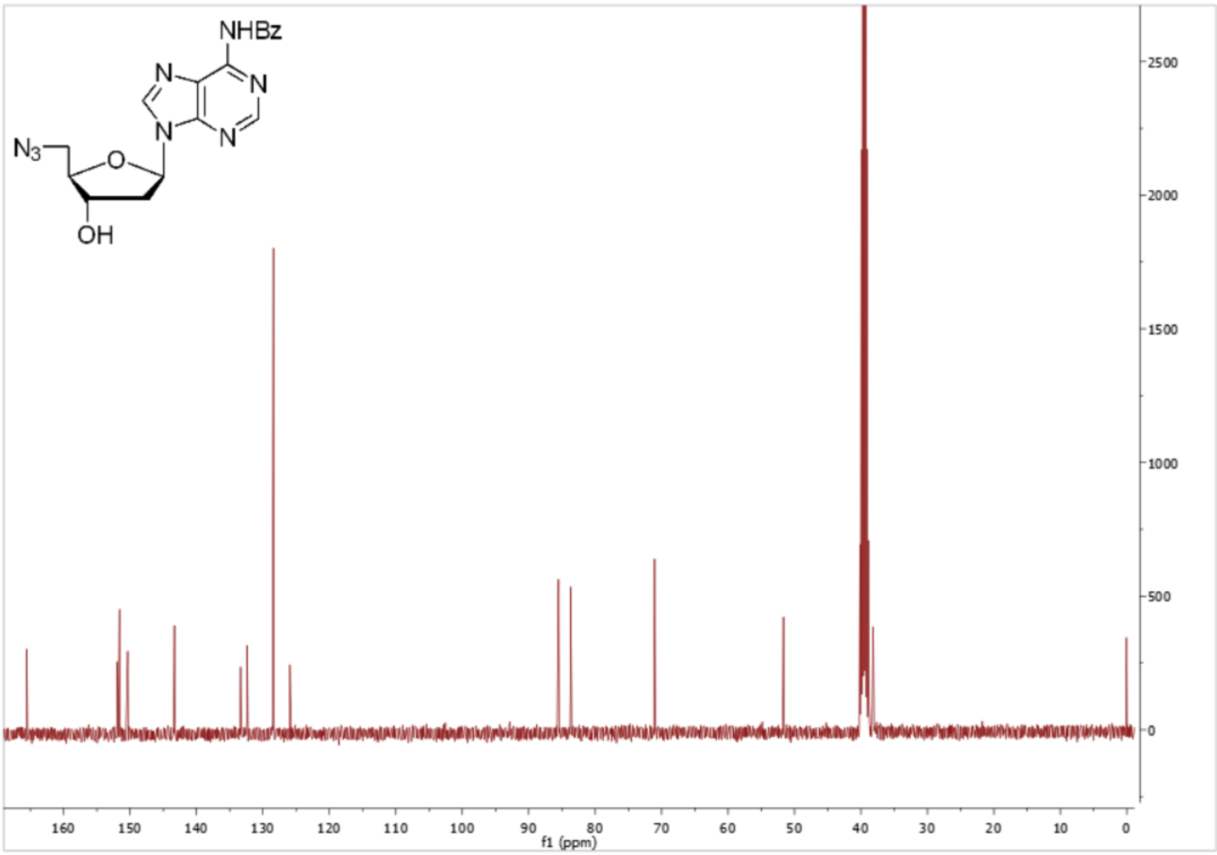
NHBz
N
N
N
N
N3
O
OH
2500
2000
1500
1000
500
0
160 150 140 130 120 110 100 90 80 70 60 50 40 30 20 10 0
f1 (ppm)

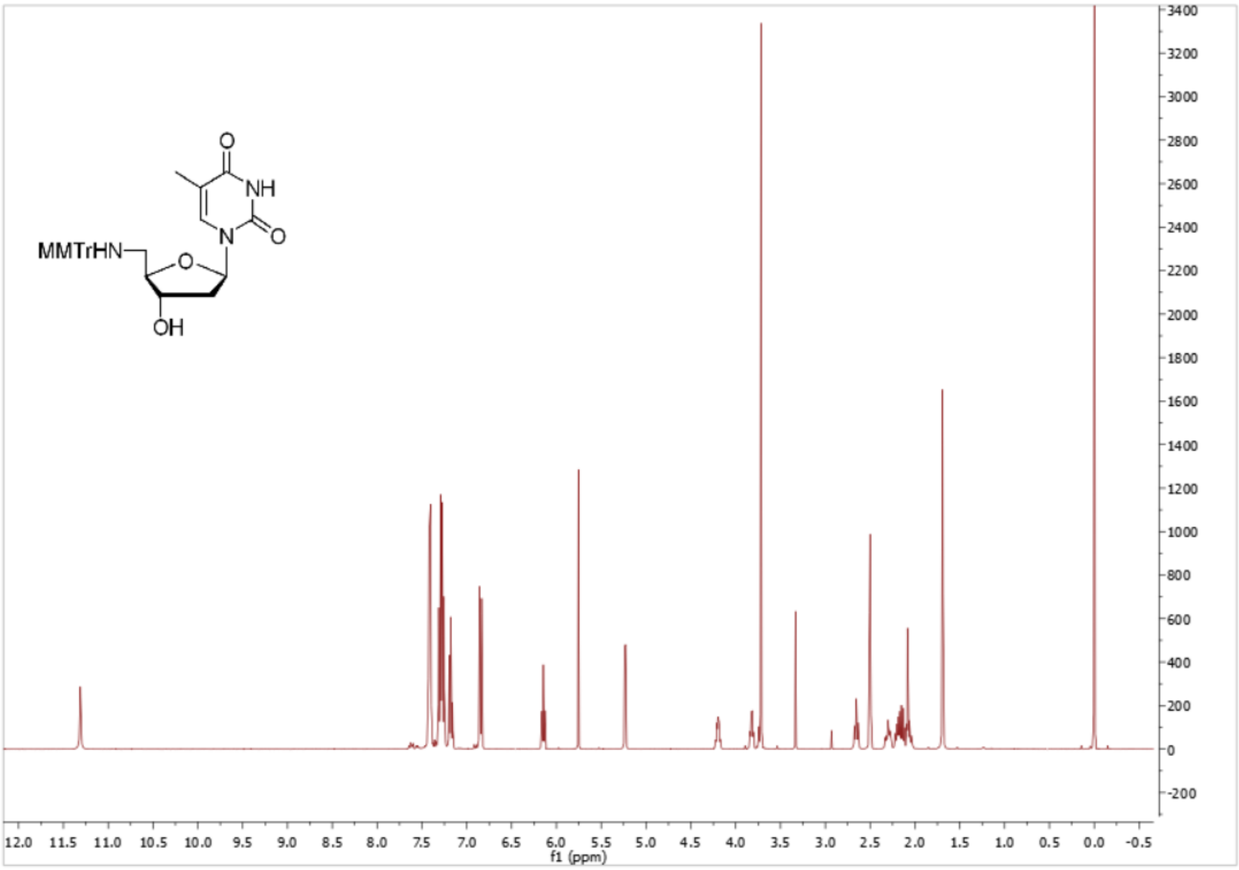
MMTrHN
OH
NH
O
N
f1 (ppm)

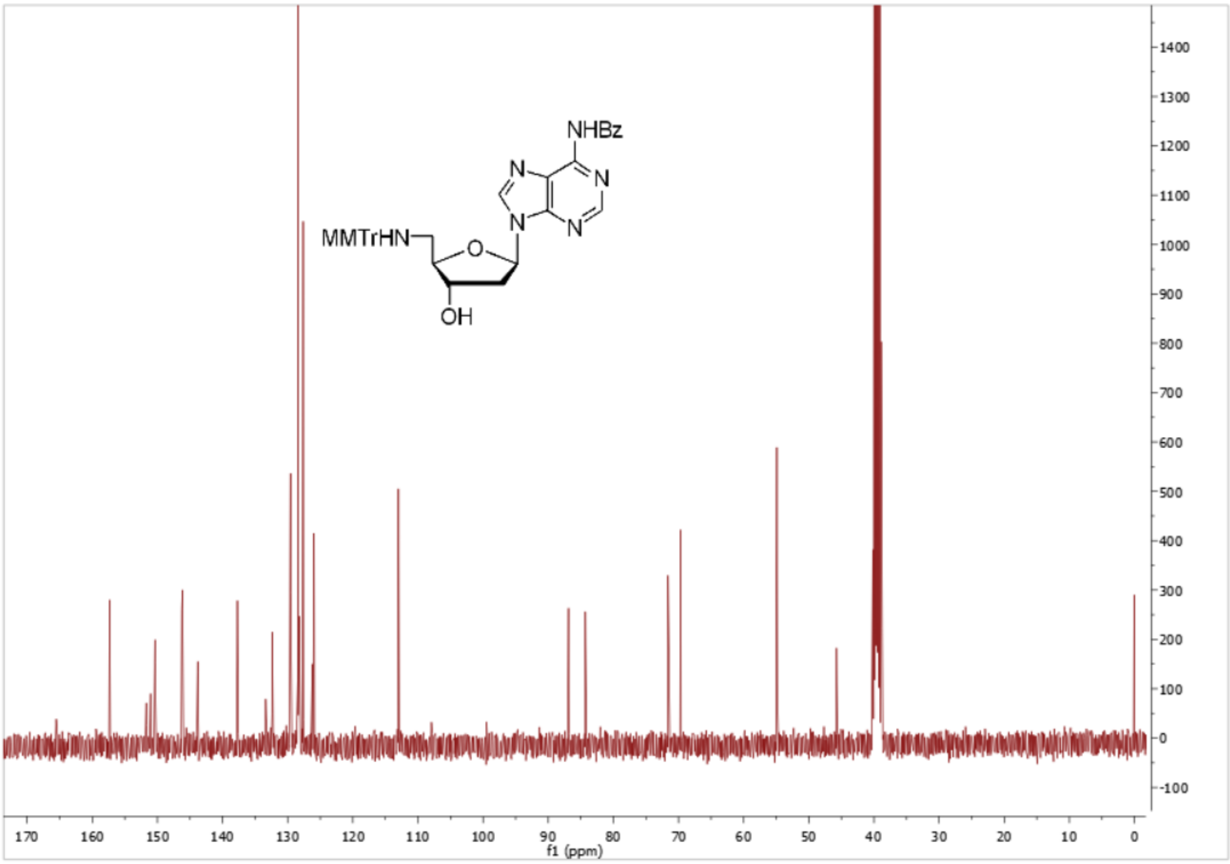
NHBz
N
MMTrHN
O
OH
f1 (ppm)

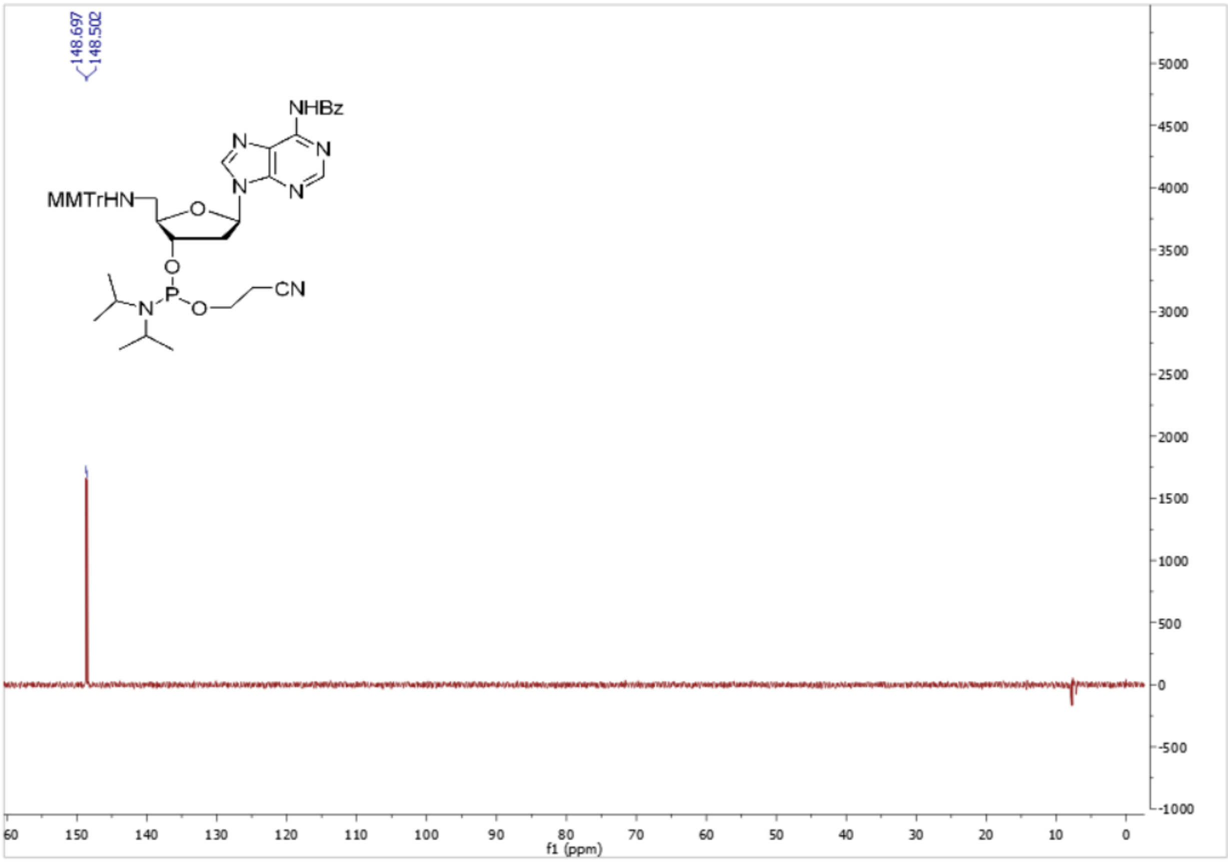
148.697
148.502
NHBz
MMTrHN
CN
f1 (ppm)

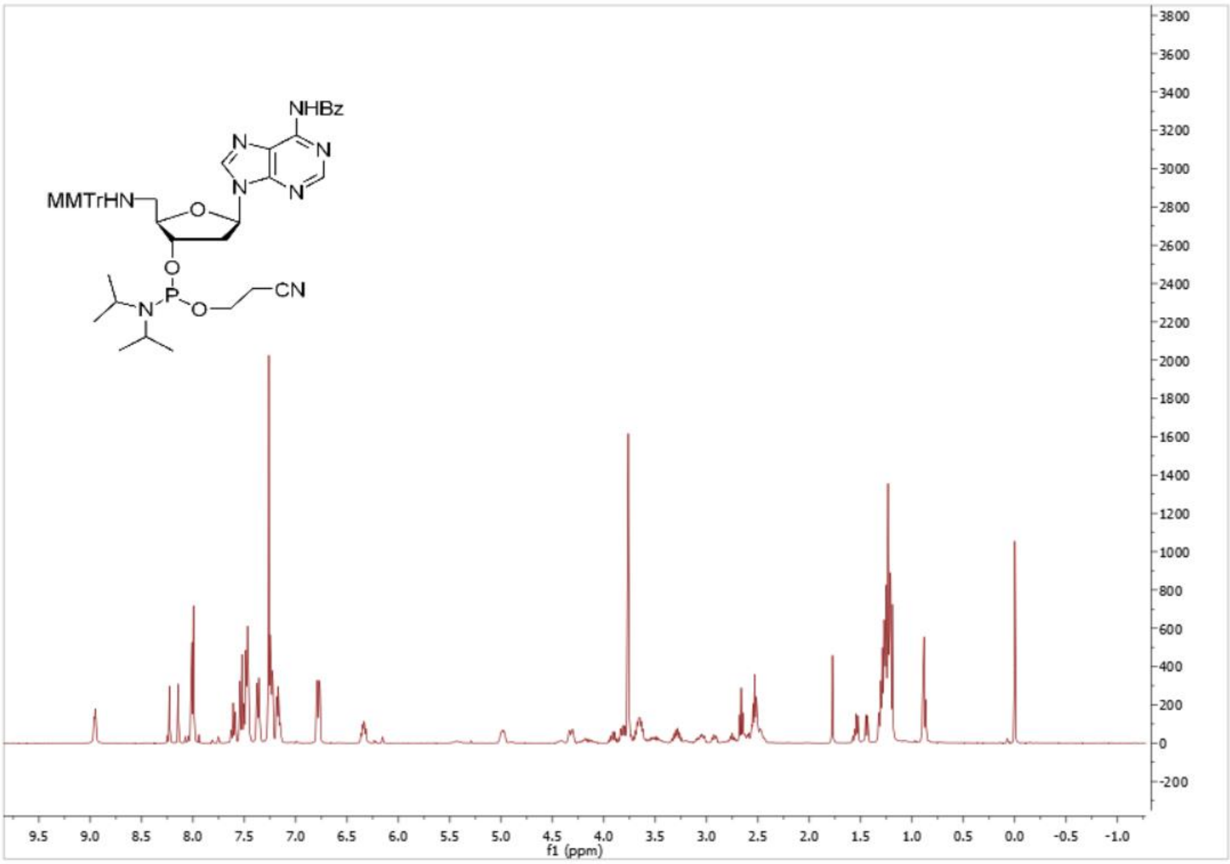
NHBz
MMTrHN
CN
f1 (ppm)

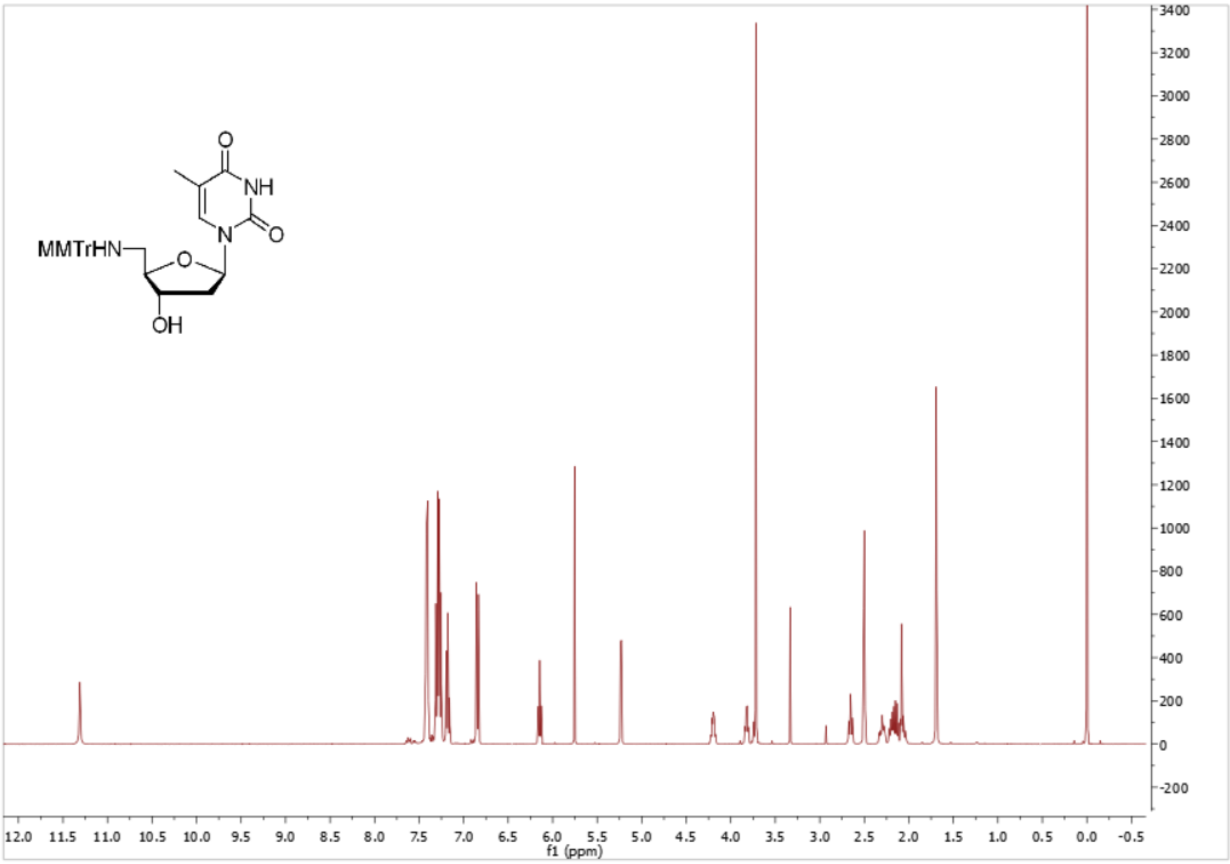
MMTrHN
O
NH
N
OH
f1 (ppm)

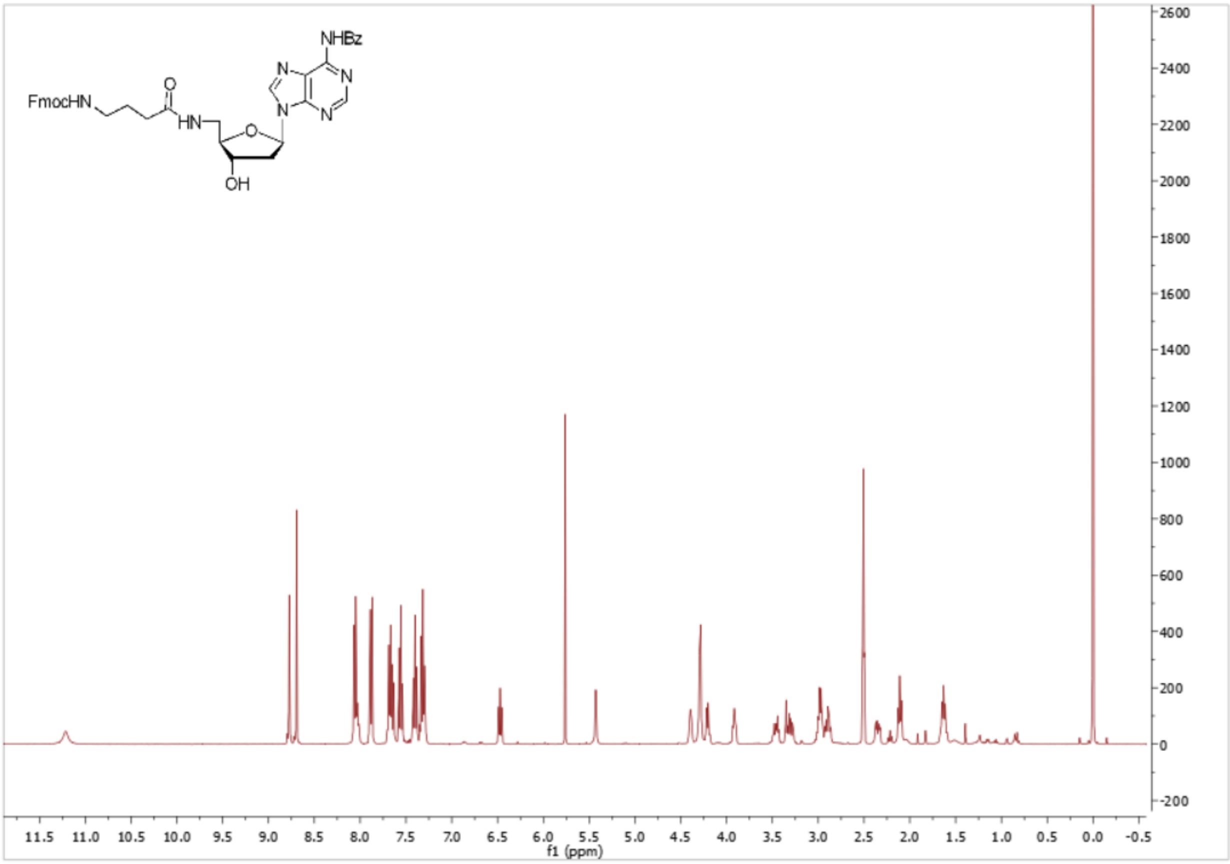
FmocHN
HN
NHBz
N
O
OH
f1 (ppm)

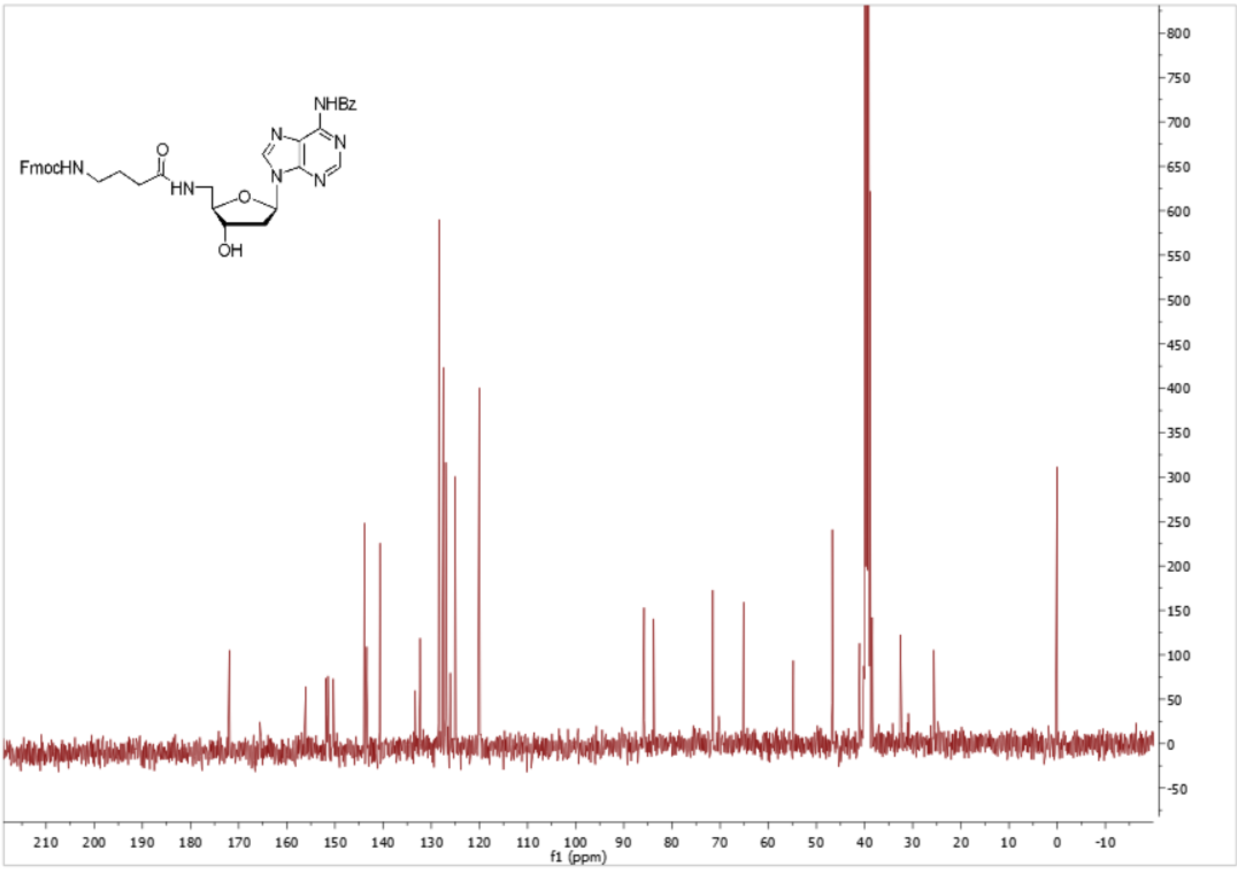
NHBz
N
N
N
N
FmocHN
O
HN
O
OH
f1 (ppm)

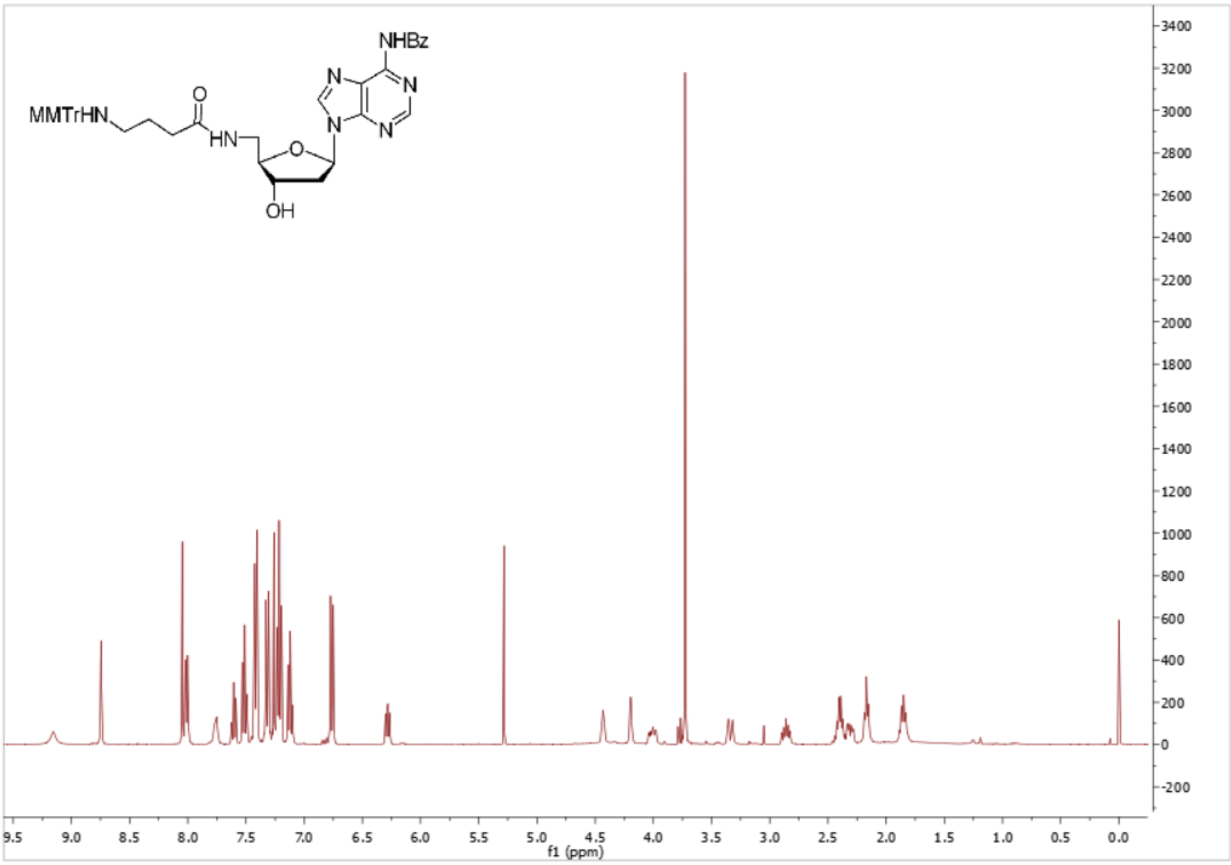
NHBz
N
N
N
N
MMTrHN
O
HN
O
OH
f1 (ppm)

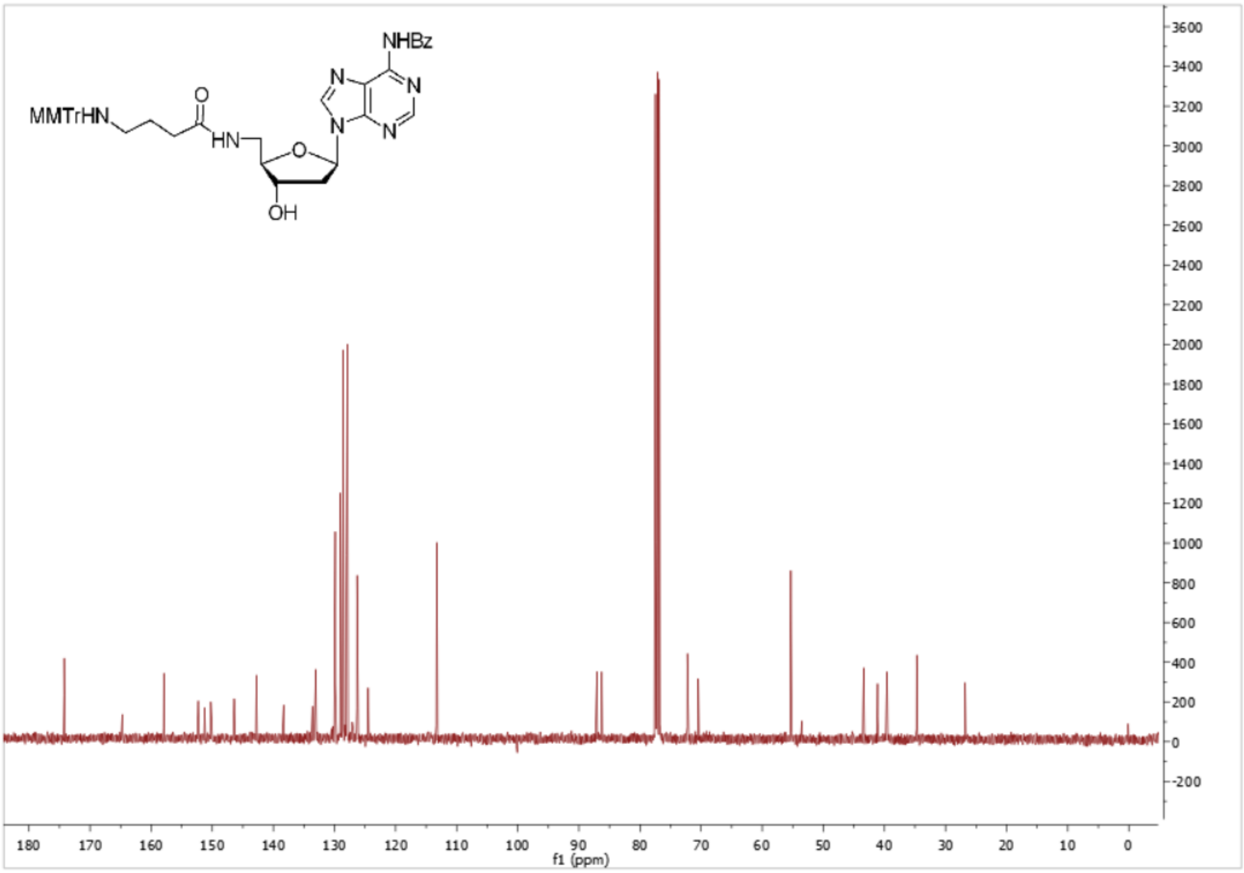
NHBz
MMTrHN
HN
OH
f1 (ppm)

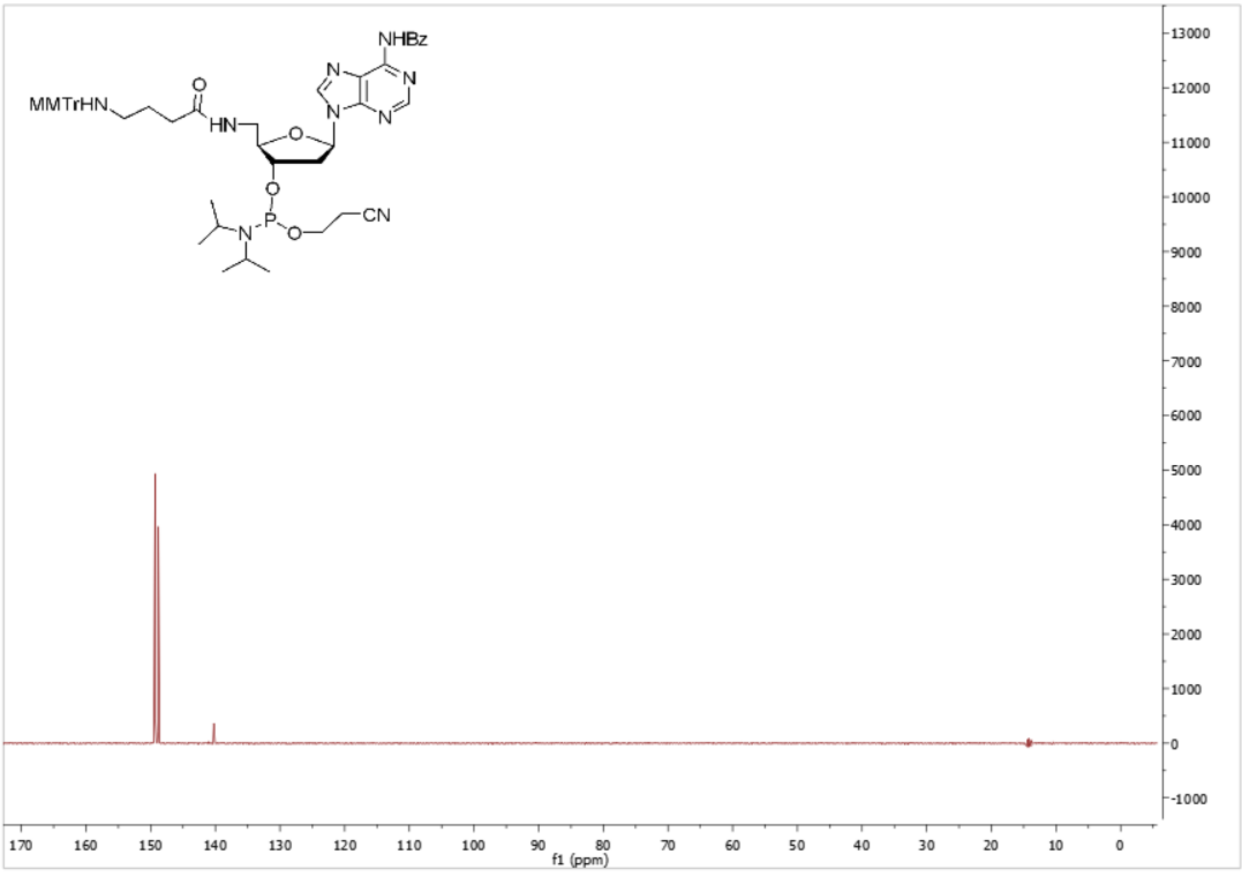
NHBz
MMTrHN
HN
CN
f1 (ppm)

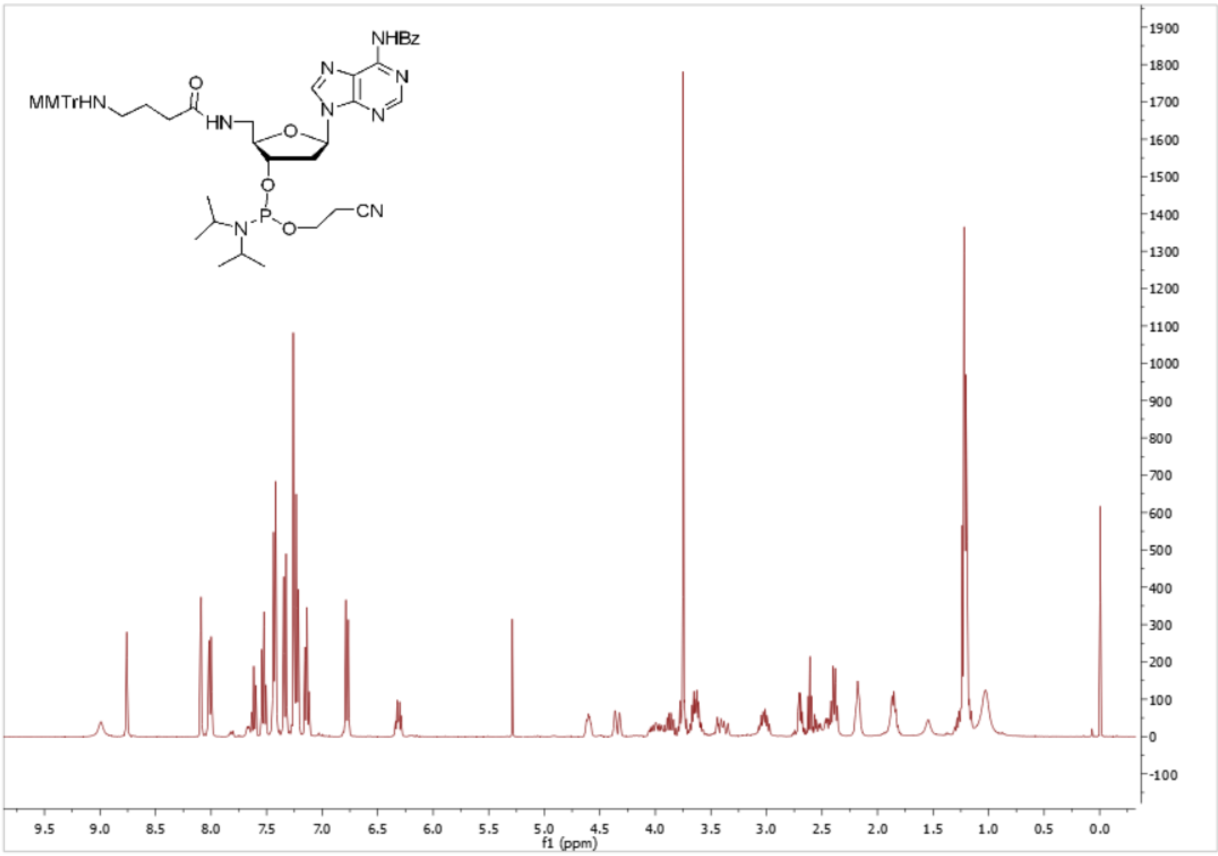
NHBz
MMTrHN
HN
O
N
CN
f1 (ppm)

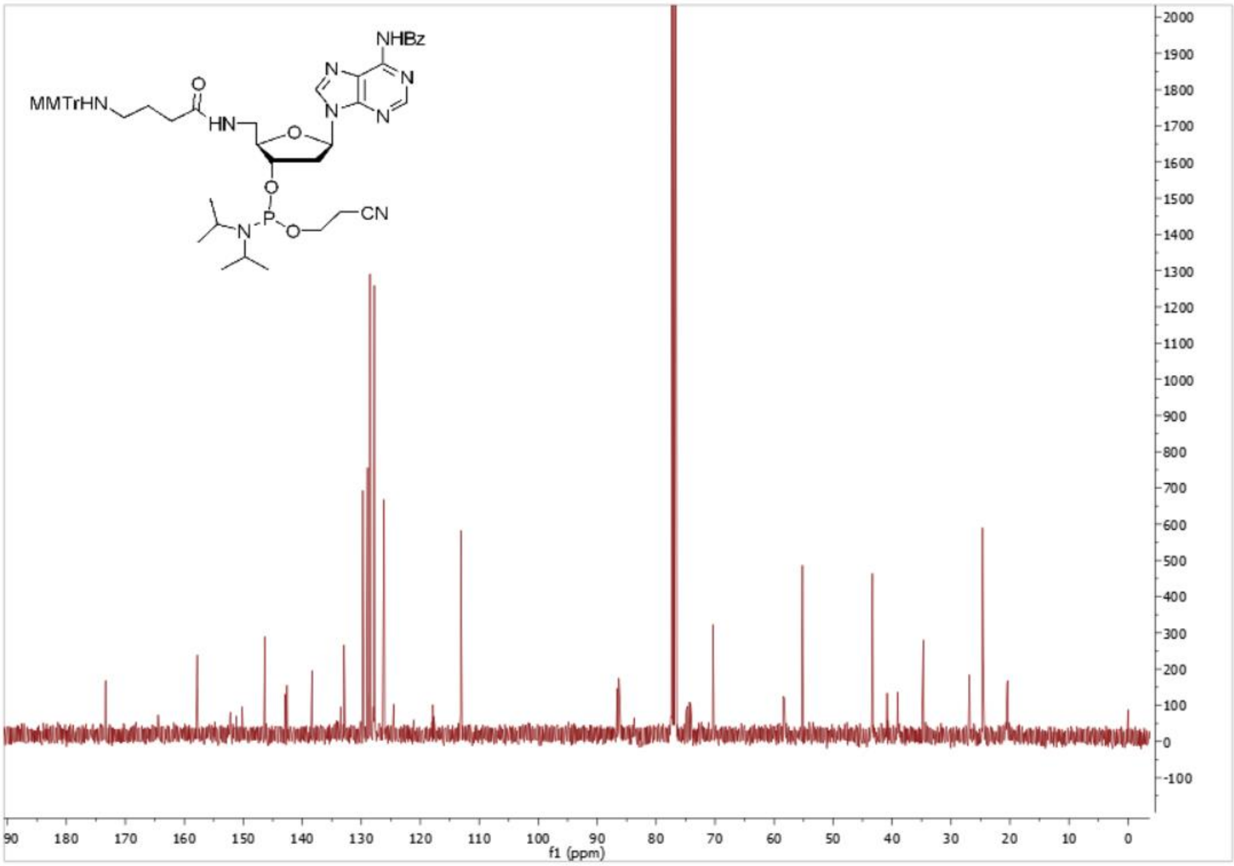
NHBz
MMTrHN
HN
O
N
CN
f1 (ppm)

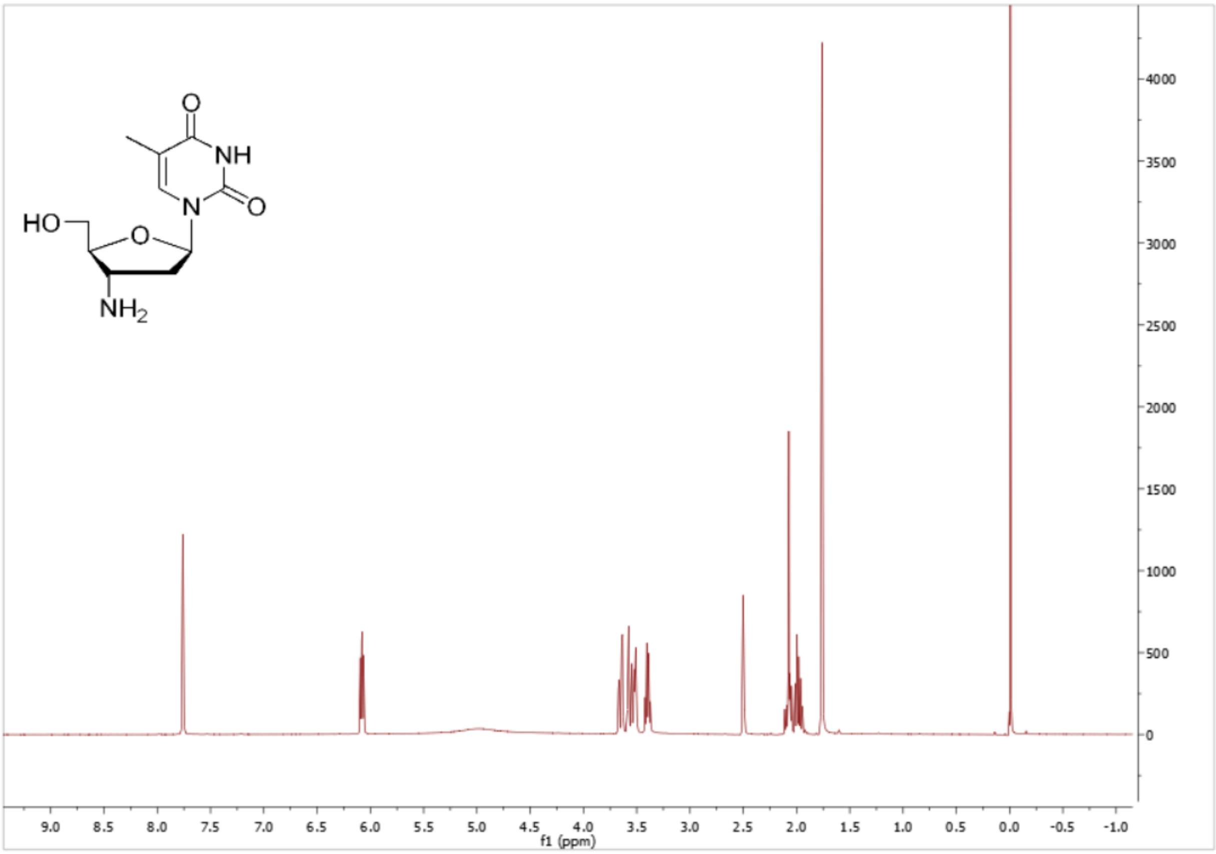
HO
O
NH
O
N
O
NH2
f1 (ppm)

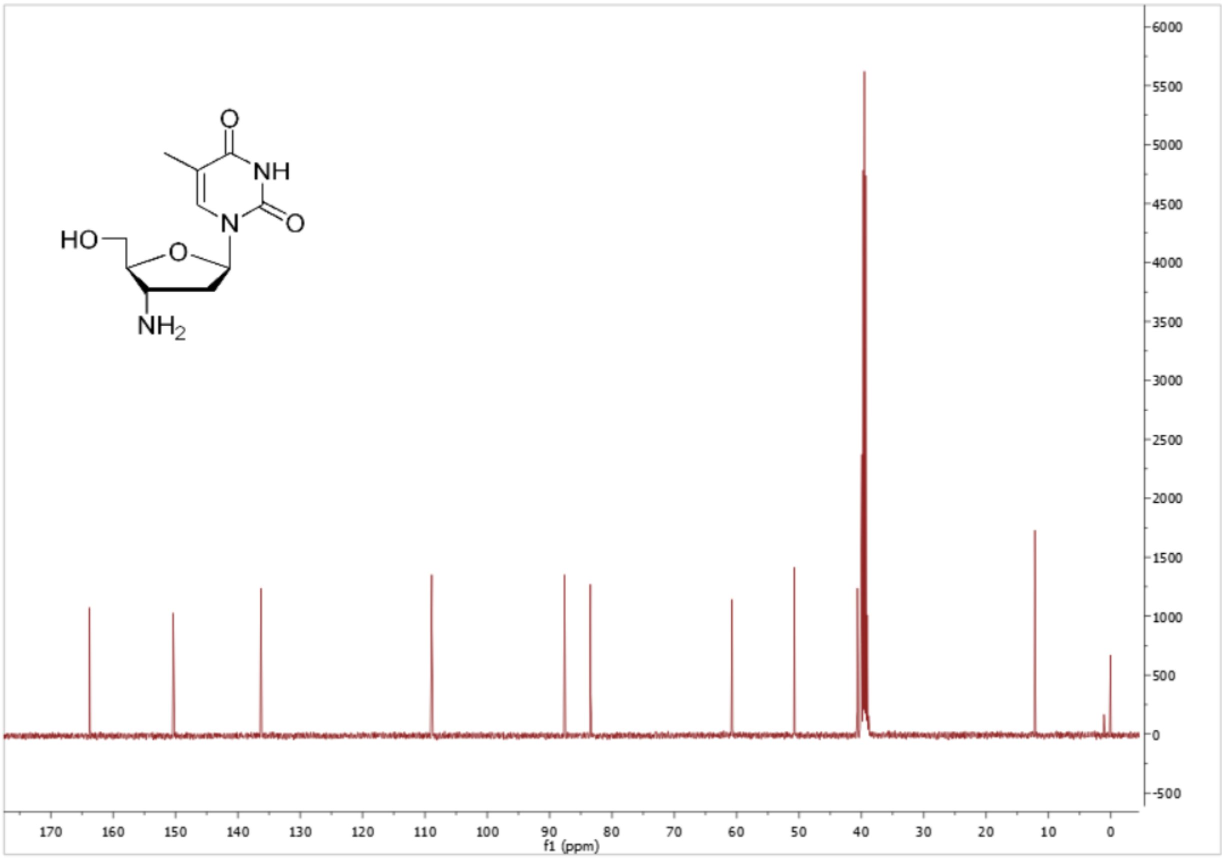
HO
O
NH
O
N
O
NH2
f1 (ppm)

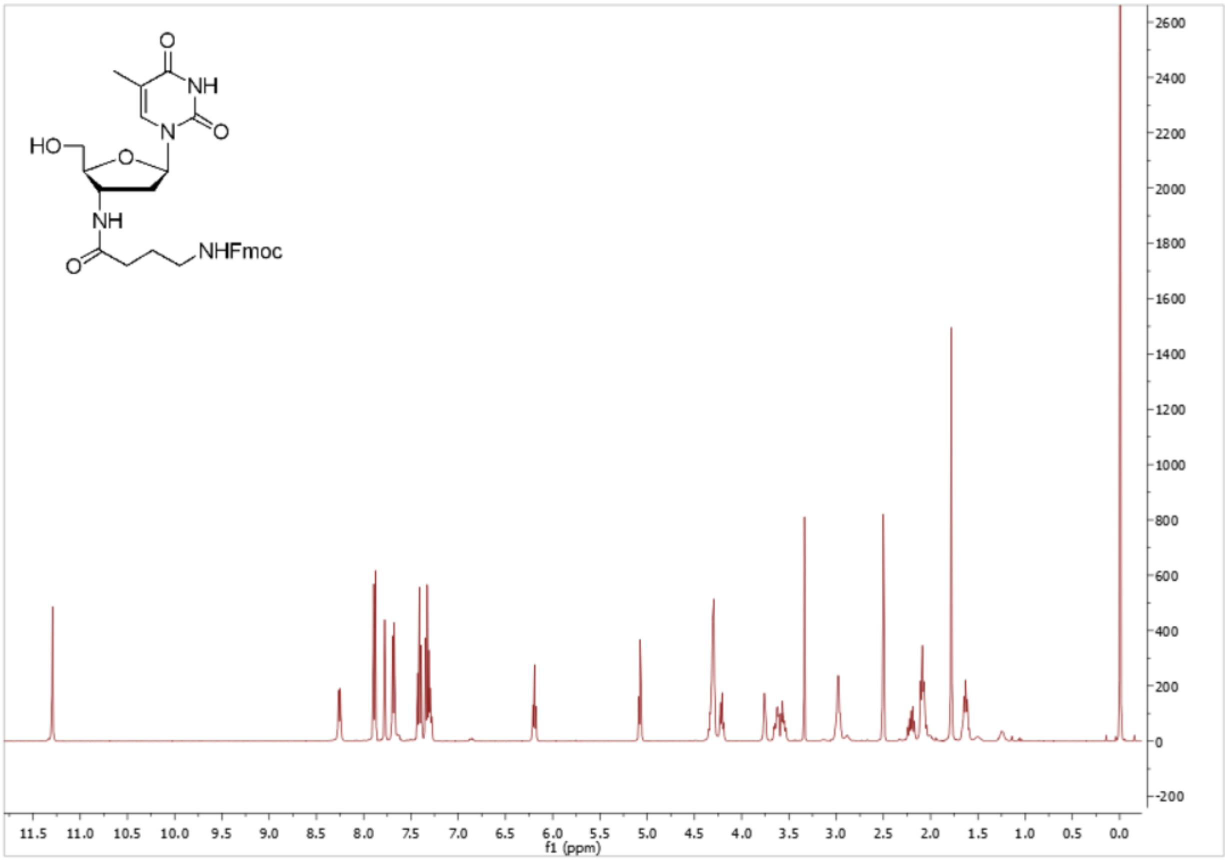

HO
O
N
NH
O
O
NH
O
NHFmoc
f1 (ppm)

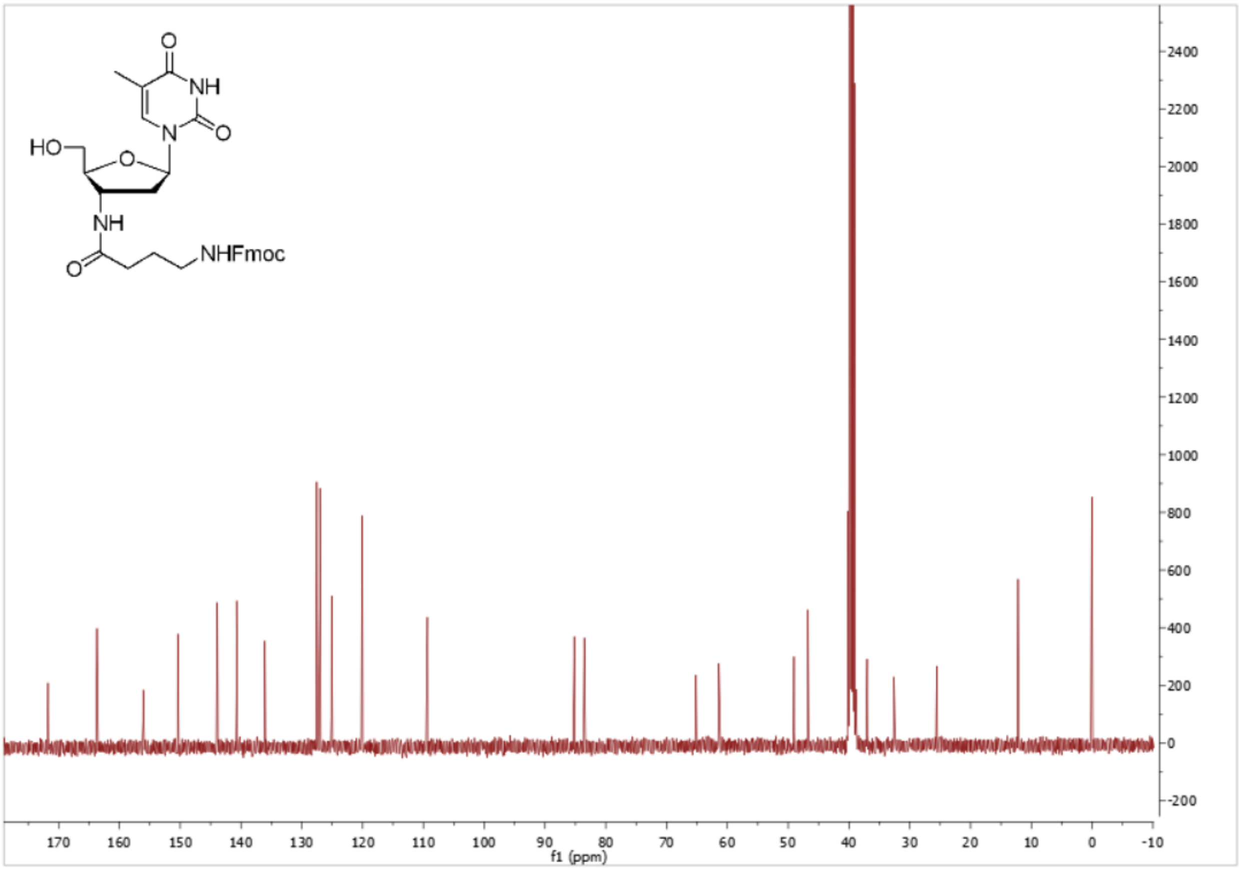

HO
O
N
NH
O
O
NH
O
NHFmoc
f1 (ppm)

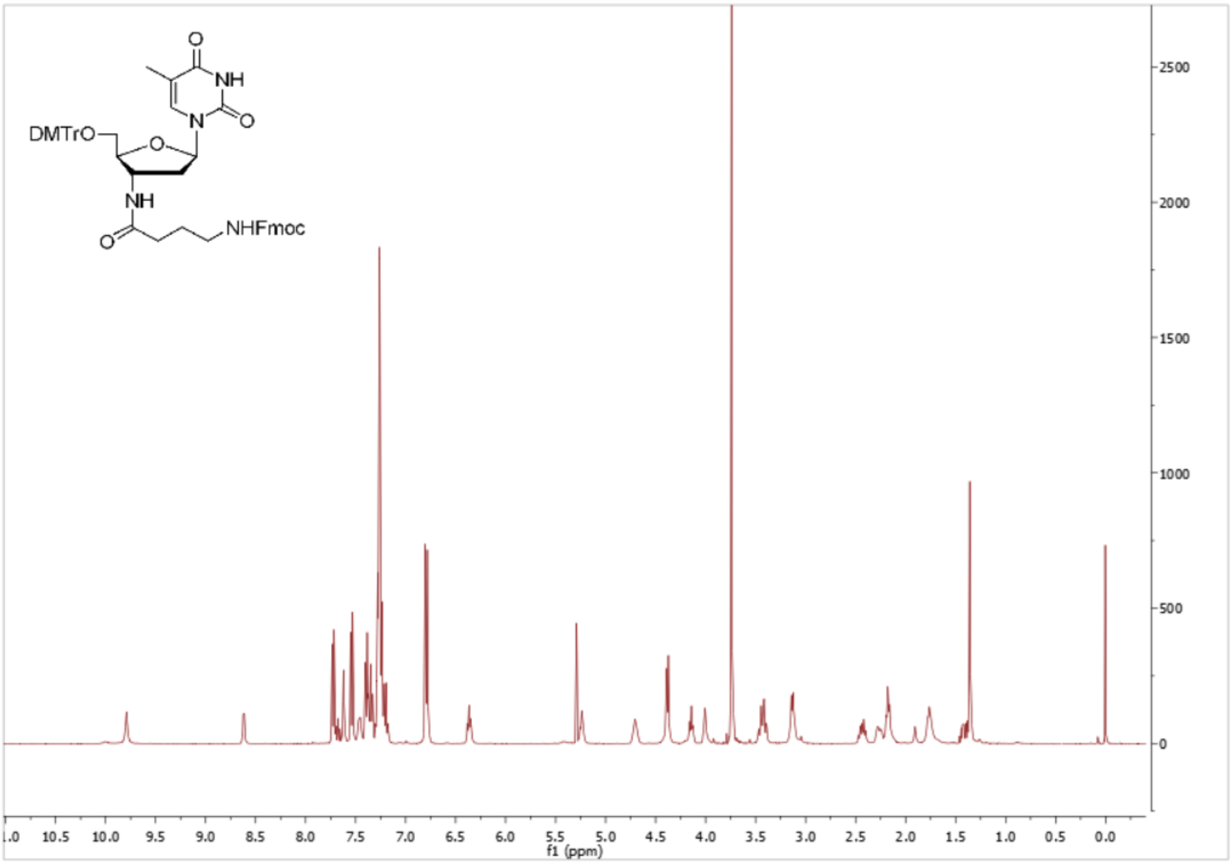
DMTrO
NH
NHFmoc
f1 (ppm)

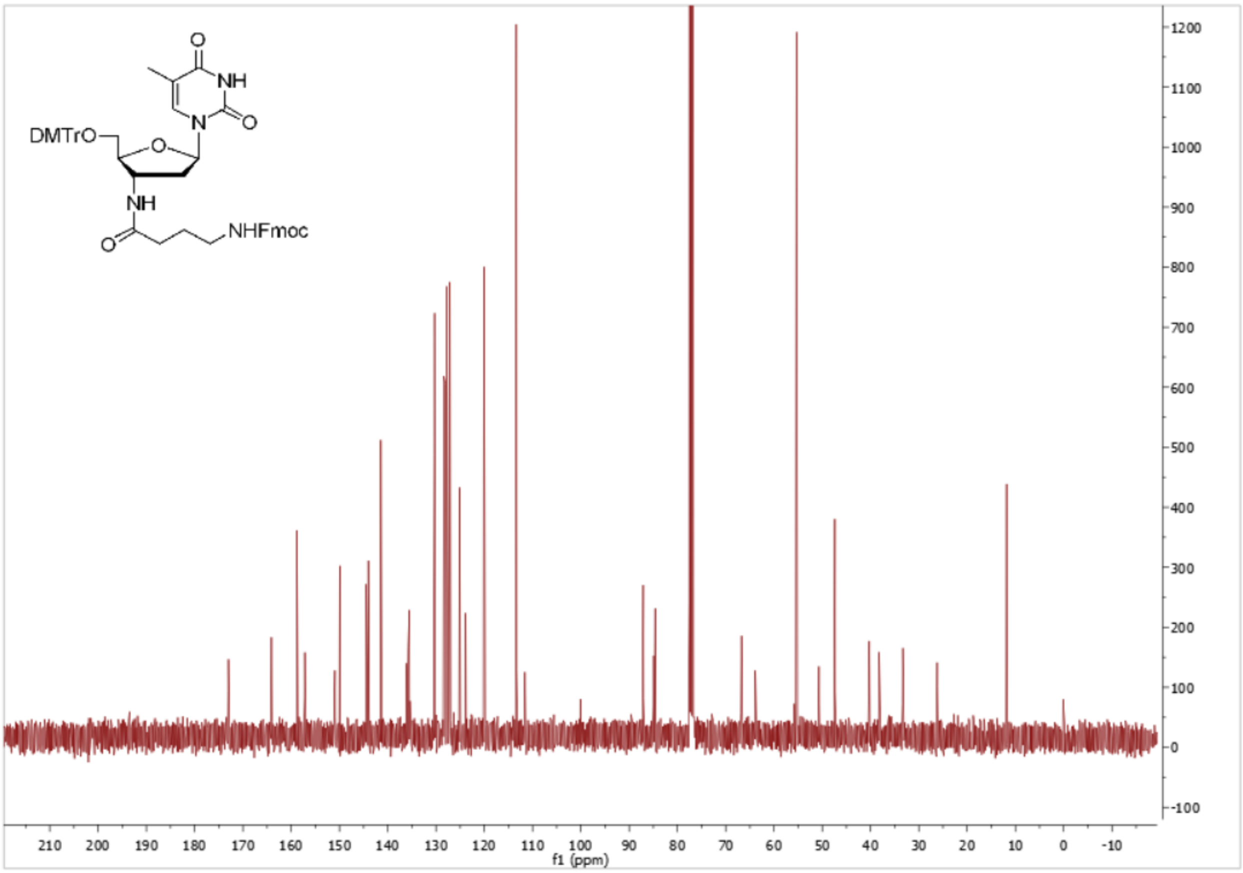
DMTrO
NH
NHFmoc
f1 (ppm)

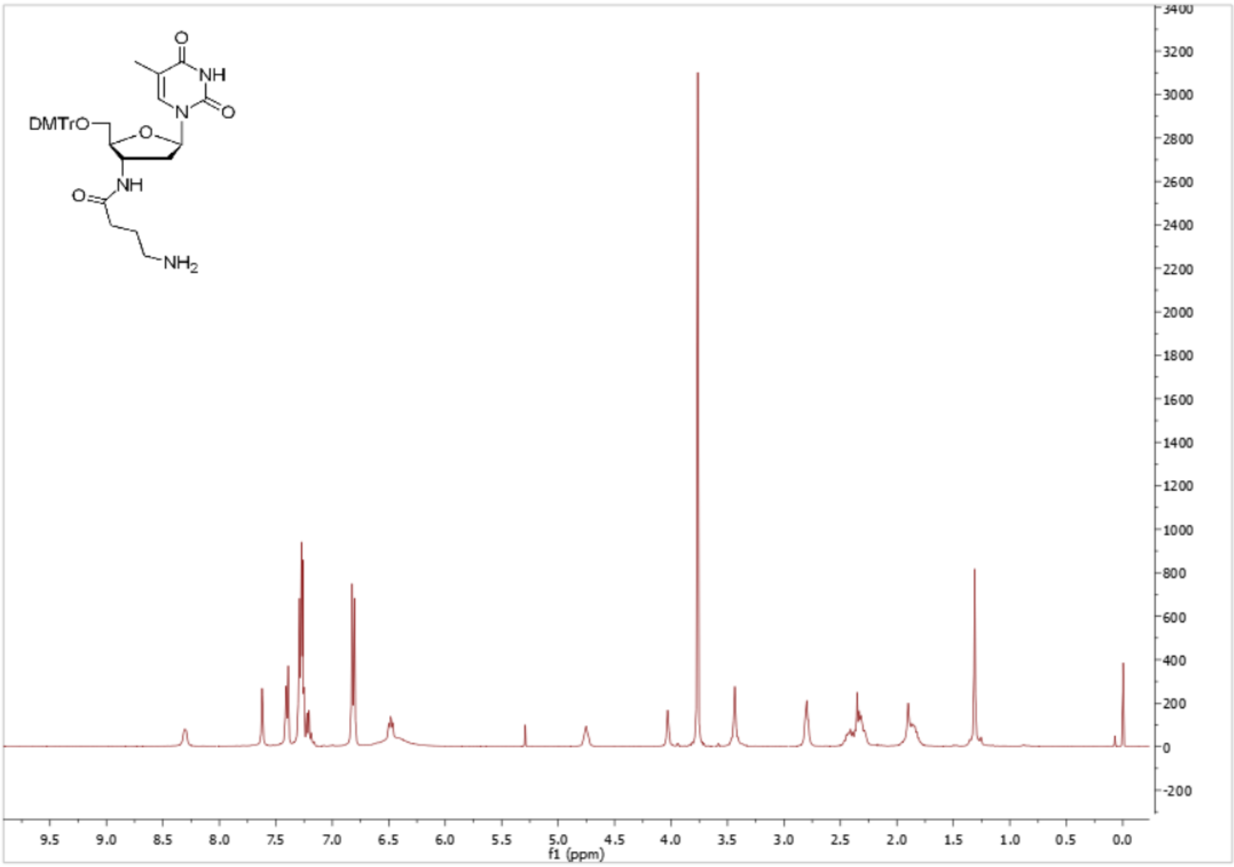
DMTrO
NH
NH
NH2
O
N
f1 (ppm)

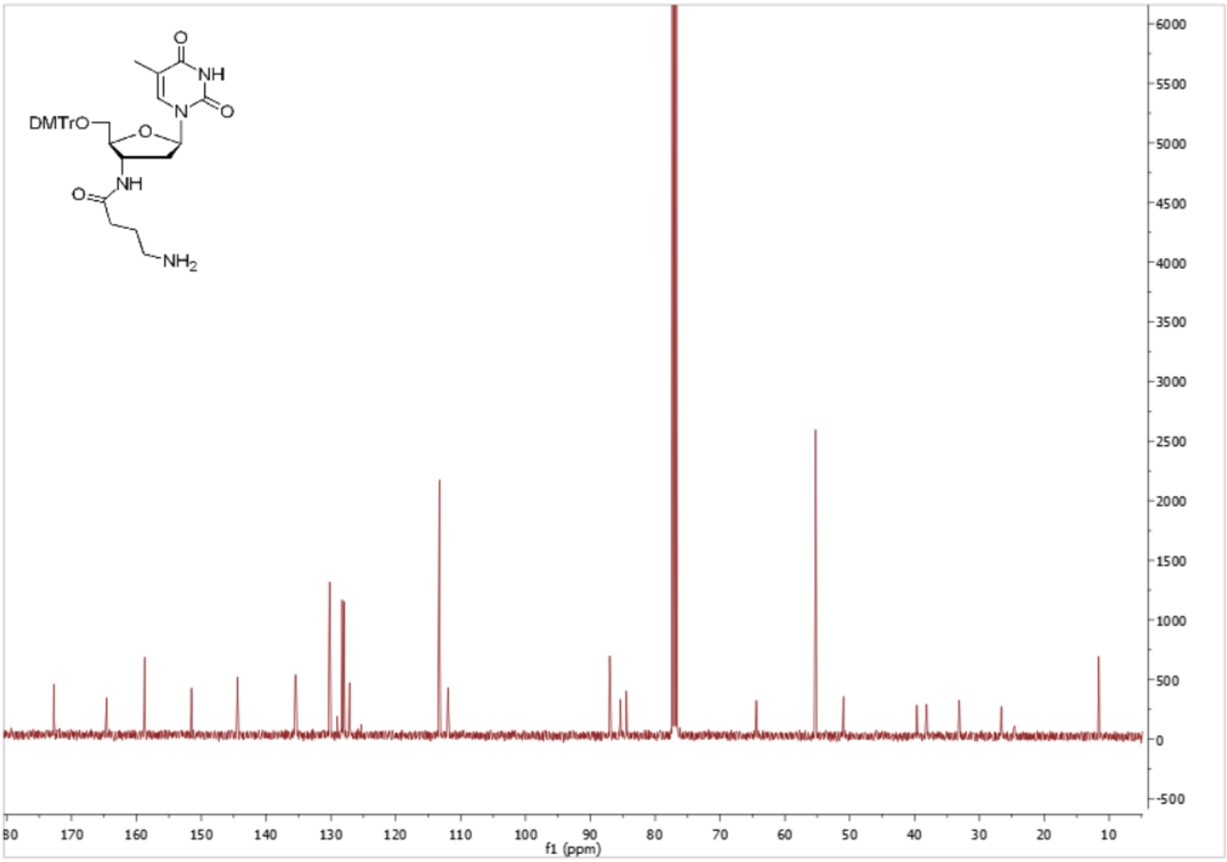
DMTrO
NH
NH
NH2
O
N
f1 (ppm)

References

1. Mag, M. & Engels, J. W. Synthesis and selective cleavage of oligodeoxyribonucleotides containing non-chiral internucleotide phosphoramidate linkages. *Nucleic Acids Res.*, **17,** 5973-5988 (1989).
2. Richardson, C. C. Phosphorylation of nucleic acid by an enzyme from T4 bacteriophage-infected Escherichia coli. *Proc. Natl. Acad. Sci. U. S. A.*, **54,** 158-165 (1965).
3. Mag, M. & Engels, J. W. Synthesis and selective cleavage of oligodeoxyribonucleotIdes containing non-chiral intemucieotIde phosphoramidate linkages. *Nucleic Acids Res.*, **17,** 5973-5988 (1989).
4. Zhu, X.-F., Williams Jr, H. J. & Ian Scott, A. An improved transient method for the synthesis of N-benzoylated nucleosides. *Synth. Commun.*, **33,** 1233-1243 (2003).
5. Lin, T. S. & Prusoff, W. H. Synthesis and biological activity of several amino analogues of thymidine. *J. Med. Chem.*, **21,** 109-112 (1978).
6. Motawia, M. S., Wengel, J., Abdel-Megid, A. E. S. & Pedersen, E. B. A Convenient Route to 3′-Amino-3′-deoxythymidine. *Synthesis*, **1989,** 384-387 (1989).
7. Lou, C. *et al.* Self-assembly of DNA–peptide supermolecules: Coiled-coil peptide structures templated by d-DNA and l-DNA triplexes exhibit chirality-independent but orientation-dependent stabilizing cooperativity. *Chem. Eur. J.*, **26,** 5676-5684 (2020).
8. Petoukhov, M. V. *et al.* New developments in the ATSAS program package for small-angle scattering data analysis. *J. Appl. Crystallogr.*, **45,** 342-350 (2012).
9. Lou, C. *et al.* Peptide-oligonucleotide conjugates as nanoscale building blocks for assembly of an artificial three-helix protein mimic. *Nat. Commun.*, **7,** 12294 (2016).
10. Uversky, V. N. *et al.* Prediction of the association state of insulin using spectral parameters. *J. Pharm. Sci.*, **92,** 847-858 (2003).
11. Ogihara, N. L., Weiss, M. S., Degrado, W. F. & Eisenberg, D. The crystal structure of the designed trimeric coiled coil coil-V(a)L(d): Implications for engineering crystals and supramolecular assemblies. *Protein Sci.*, **6,** 80-88 (1997).
12. Bowers, K. J. *et al.* Scalable Algorithms for Molecular Dynamics Simulations on Commodity Clusters. SC '06: Proceedings of the 2006 ACM/IEEE Conference on Supercomputing; 2006 11-17 Nov. 2006; 2006. p. 43-43.
13. Jorgensen, W. L. *et al.* Comparison of simple potential functions for simulating liquid water. *J. Chem. Phys.*, **79,** 926-935 (1983).
14. Jensen, K. P. & Jorgensen, W. L. Halide, ammonium, and alkali metal ion parameters for modeling aqueous solutions. *J. Chem. Theory Comput.*, **2,** 1499-1509 (2006).
15. Harder, E. *et al.* OPLS3: A Force Field Providing Broad Coverage of Drug-like Small Molecules and Proteins. *J. Chem. Theory Comput.*, **12,** 281-296 (2016).
16. Bowers, K. J. *et al.* Scalable algorithms for molecular dynamics simulations on commodity clusters. Proceedings of the 2006 ACM/IEEE conference on Supercomputing; 2006; 2006. p. 84–84.
17. Humphrey, W., Dalke, A. & Schulten, K. VMD: Visual molecular dynamics. *J. Mol. Graph.*, **14,** 33-38 (1996).
18. Schneidman-Duhovny, D., Hammel, M., Tainer, J. A. & Sali, A. Accurate SAXS Profile Computation and its Assessment by Contrast Variation Experiments. *Biophys. J.*, **105,** 962-974 (2013).
19. Schneidman-Duhovny, D., Hammel, M., Tainer, J. A. & Sali, A. FoXS, FoXSDock and MultiFoXS: Single-state and multi-state structural modeling of proteins and their complexes based on SAXS profiles. *Nucleic Acids Res.*, **44,** W424-W429 (2016).
