# Supplementary material for: Chirality transmission in macromolecular domains
Source: Nat Commun. 2022 Jan 10;13:76. doi: 10.1038/s41467-021-27708-4 (PMC8748818; doi:10.1038/s41467-021-27708-4)
Supplement: Supplementary file 2 — Description of Additional Supplementary Files [file 41467_2021_27708_MOESM2_ESM.docx]

**Description of Additional Supplementary Files:**

**Supplementary Movie 1:** The oligonucleotide domain is shown in yellow, ochre, and orange. The peptide domain is shown in blue, red, and grey. The linkers are shown in green. Solvent (water) is not shown for clarity.
